# Supplementary material for: Predicting resistance to chemotherapy using chromosomal instability signatures
Source: Nat Genet. 2025 Jun 23;57(7):1708–17. doi: 10.1038/s41588-025-02233-y (PMC12283407; doi:10.1038/s41588-025-02233-y)
Supplement: Supplementary file 1 — Supplementary Methods, Notes 1–3, Figs. 1–80 and References. [file 41588_2025_2233_MOESM1_ESM.pdf]

nature genetics

Supplementary information https://doi.org/10.1038/s41588-025-02233-y

Predicting resistance to chemotherapy using chromosomal instability signatures

In the format provided by the authors and unedited

Predicting resistance to chemotherapy using chromosomal instability signatures

- Supplementary Information -

Supplementary Notes **4**
Supplementary Note 1 - Tolerance to micronuclei and anthracycline resistance 4
Supplementary Note 2 - Accounting for platinum effects when predicting treatment resistance in relapsed ovarian cancer 7
Predicting resistance to taxane treatment in relapsed ovarian - OV04 7
Predicting resistance to anthracycline treatment in relapsed ovarian - OV04 7
Predicting resistance to taxane treatment in relapsed ovarian - TCGA 8
Predicting resistance to anthracycline treatment in relapsed ovarian - TCGA 8
Supplementary Note 3 - Assessing performance of HRDetect and Myriad myChoice 10
Supplementary Methods **12**
Data curation and annotation of sample cohorts 12
The Cambridge Translational Cancer Research Ovarian Study 04 (OV04) 12
Clinical data 12
Organoids and spheroids 13
Patient tissue samples 13
Plasma samples 14
The Cancer Genome Atlas collection 14
Treatment history information 14
Annotation of breast tumour subtypes 15
Annotation of BRCA mutation status 15
The Hartwig Medical Foundation dataset 15
Annotation of weighted Genome Instability Index (wGII) 15
Biomarker development and optimisation 16
Clinical classifier of platinum resistance 16
Clinical classifier of taxane resistance 16
Clinical classifier of anthracycline resistance 17
Doxorubicin treatment assays 17
Treatment of cell lines 17
Treatment of spheroids and organoids 17
Micronuclei counting 18
Estimating expected number of micronuclei 19
Optimal threshold selection in vitro 20
Gene enrichment analysis 20

Survival period calculation 21
Progression-free survival (PFS) 21
PFS for platinum treatment in OV04 21
PFS for anthracycline and taxane treatment in OV04 21
Time to Treatment Failure (TTF) 22
TCGA 22
HMF 22
Biomarker performance evaluation 22
Power Analysis 22
Phase II single-arm trial emulation 23
Phase III randomised controlled study emulation 24
Enrichment trial design emulation 24
Inverse Probability Weighting 25
Performance comparison with alternative metrics 26
HRDetect and Myriad myChoice classifier 26
Copy number signature activities 26
Copy number feature components 26
Supplementary Figures 27
Supplementary References 98

Supplementary Notes

Supplementary Note 1 - Tolerance to micronuclei and anthracycline resistance

Anthracyclines can cause DNA damage resulting in extrachromosomal DNA (ecDNA) encapsulated in micronuclei[1], and thus tumours resistant to anthracyclines may tolerate the ongoing formation of micronuclei. To identify such resistant tumours, we rely on three specific CIN signatures associated with high-level copy number changes of small segments (CX8, CX9 and CX13). Existing studies show that low doses of the anthracycline doxorubicin can induce micronuclei formation in cancer cell lines[2]. Therefore, to test if the presence of these amplification-related signatures was associated with any modulation in micronuclei formation rates, we treated a panel of four ovarian cancer cell lines with low dose doxorubicin and observed micronuclei induction rates using fluorescent imaging (**Supplementary Note Fig. 1a**). We performed shallow whole genome sequencing on the cell lines prior to treatment, computed CIN signatures and also estimated the expected number of induced micronuclei using a model of micronuclei induction and inheritance (see **Supplementary Methods**). Cell lines with high activity of amplification-related signatures (CX8, CX9 and CX13) showed fewer than expected micronuclei, whereas the cell line with no activity of amplification-related CIN signatures showed the expected number of micronuclei (**Supplementary Note Fig. 1b**). This suggests cells with amplification-related signatures have a reduction in DNA damage and potential genome stabilisation.

We then aimed to construct and optimise a signature-based biomarker for predicting resistance to anthracycline treatment *in vitro*. We treated a cohort of 23 ovarian cancer patient-derived models (8 organoids and 15 spheroids) with the anthracycline doxorubicin, measured response via IC50 to then classify models as resistant or sensitive based on the expected number of sensitive cases (see **Supplementary Methods**), and explored activity of amplification-related signatures computed from shallow whole-genome sequencing prior to treatment. We then used a grid search to explore a range of activity values for the three amplification-related signatures and determine the optimal activity threshold for maximising specificity. Maximising specificity facilitates identifying patients resistant to anthracyclines without preventing those who are sensitive from receiving the therapy. In this cohort, all sensitive models showed an activity of CX8, CX9 and CX13 lower than 0.01 (100% specificity), and 3 of the 14 resistant models showed activity lower than 0.01 (82% sensitivity). Therefore, thresholds of CX8>0.01, CX9>0.01 and CX13>0.01 were selected as optimal for identifying resistant models (**Fig. 1f**).

Tolerance to micronuclei formation is likely mediated by a switch from cGAS-STING to noncanonical NF-κB signalling, which in turn can activate homologous recombination[3]. Therefore, we sought evidence that tumours predicted to be resistant to doxorubicin treatment had undergone this switching mechanism. For TCGA ovarian cancers we applied our anthracycline classifier, splitting patients in two groups, predicted resistant or predicted sensitive. We then performed a differential expression analysis between the groups and

determined pathways that were either enriched in the predicted resistant group or suppressed (**Supplementary Note Fig. 1c**). We found evidence that pathways previously associated with cGAS-sting activation were repressed such as G2/M checkpoint and E2F targets[4]. The enriched pathways show evidence of chronic cGAS-STING activation via activation of pathways downstream of cGAS-STING such as interferon response genes, but also evidence of non-canonical NF-κB signalling via activation of NF-κB signalling pathways coupled with genes associated with epithelial-to-mesenchymal transition (EMT)[5].

Together, these data support a model of anthracycline resistance that is mediated by suppressed micronuclei induction and chronic cGAS-STING activation, leading to non-canonical NF-κB signalling mediated immune suppression and genome stabilisation. Importantly, these tumours which have acquired resistance can be identified using CIN signatures representing amplifications.

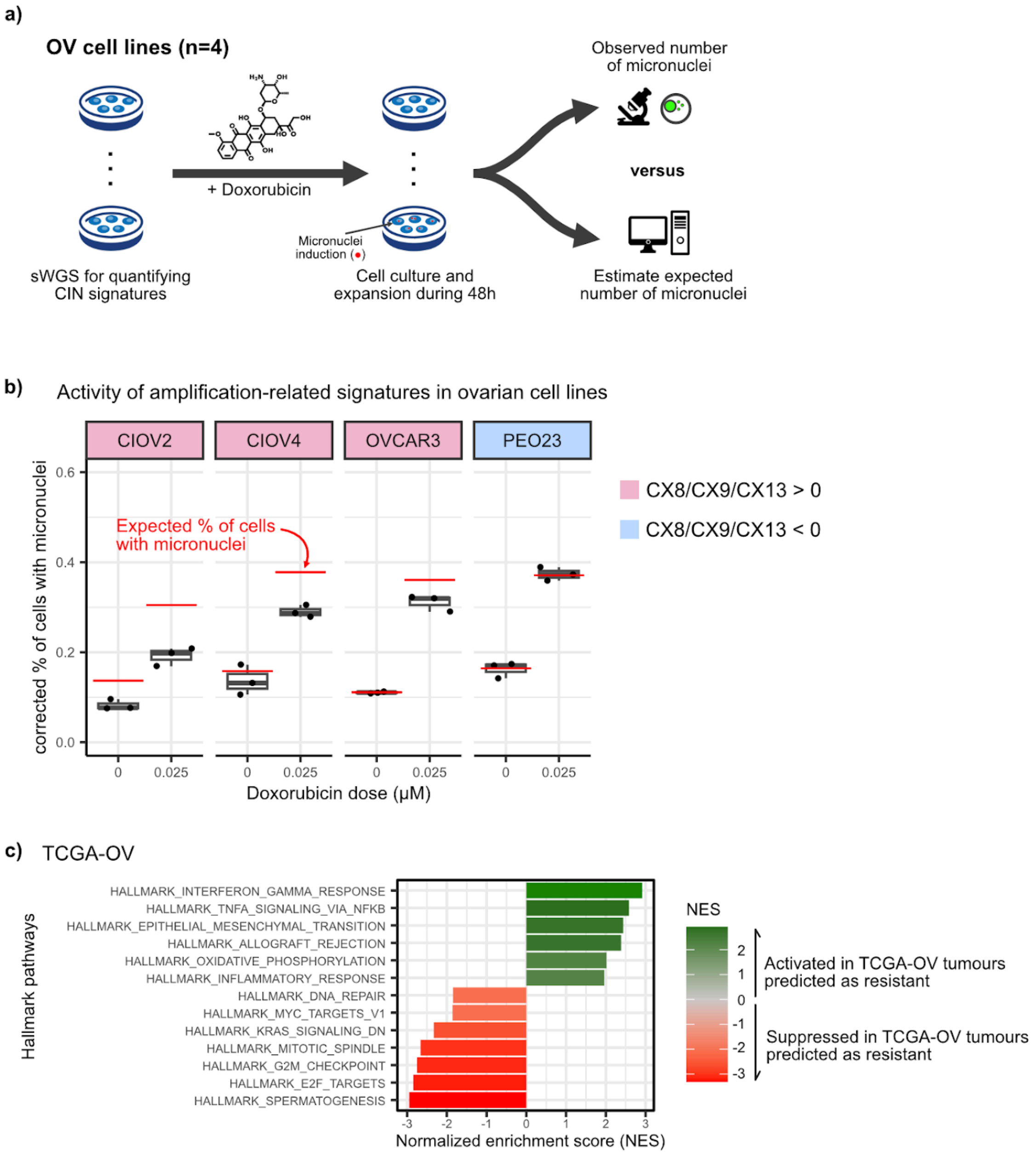

Supplementary Note Figure 1. Predicting doxorubicin response using copy number signatures linked to extrachromosomal DNA. a) Overview of experimental design for exploring the presence of amplification-related CIN signatures (CX8, CX9 and CX13) and micronuclei induction. **b)** Evaluation of micronuclei induction under doxorubicin treatment. Boxplots show the observed frequency of cells with micronuclei (y-axis) in the presence of 0 and 0.025μM of doxorubicin. Red line indicates the expected frequency of cells with micronuclei according to growth rates and micronuclei persistence across cell divisions. Boxes with centre line at the median, upper bound at 75th percentile, lower bound at 25th percentile, and whiskers at minimum and maximum values. Each dot represents one replicate. **c)** Gene set enrichment analysis results showing HALLMARK gene sets that are highly enriched in TCGA-OV tumours (n=382) predicted as resistant compared with those predicted as sensitive to doxorubicin. Only significant results after FDR correction are shown (q-value < 0.05).

Supplementary Note 2 - Accounting for platinum effects when predicting treatment resistance in relapsed ovarian cancer

Response to second-line treatment in relapsed ovarian cancer has been shown to be heavily influenced by the first-line platinum treatment-free interval[6]. As first-line treatment in our ovarian cohorts is platinum-based, the effect of this therapy can heavily influence PFS or TTF intervals for subsequent taxane or doxorubicin treatment, whether or not these were given in combination with platinum. At second-line, the effect of platinum-free treatment is likely strong enough to warrant including an interaction between first-line platinum PFS/TTF and the main predictor/experimental treatment. For subsequent lines, the patient is more likely to have become platinum resistant and thus the effect will be diminished and any effect of platinum sensitivity may not need to be accounted for using an interaction term. Avoiding the use of an interaction term is preferable to avoid any multiplicative effect of the resulting HR. When an interaction is to be included in the model, the HR can change over the different levels of first-line PFS/TTF. This can have a multiplicative effect on the final HR reported, potentially distorting interpretation. Therefore, to account for this and provide a more reasonable estimate of the HR, it is preferable to provide a point estimate for the HR, i.e. a HR estimated for a fixed first-line PFS or TTF. As we want to minimise the impact of platinum sensitivity on the HR we chose to report the HR computed at 6 months after first line treatment estimated using a restricted cubic splines approach[7]. This point represents the time at which a patient would be considered platinum-resistant and thus represents the point at which the effect of platinum is minimised.

Here, for each of the ovarian cohorts where we predicted taxane or anthracycline resistance, we report how we accounted for the effect of first-line platinum-based treatment.

Predicting resistance to taxane treatment in relapsed ovarian - OV04

For patients in the OV04 cohort treated with taxane-based therapy, the majority received their therapy at third-line or later (**Supplementary Note Fig. 2a**), with 90% treated with taxane as a single agent (**Extended Data Fig. 4**). Given the bias towards later lines and minimal effect from co-treatment with platinum, we did not use an interaction term in the Cox model. Rather, we elected to control for the treatment line under the assumption that lines closer to first-line might experience stronger effects compared to later lines. For completeness, we tested the model with an interaction, however, this did not have a significant impact.

Predicting resistance to anthracycline treatment in relapsed ovarian - OV04

For patients in the OV04 cohort treated with anthracycline-based therapy, most received anthracyclines at second-line (**Supplementary Note Fig. 2b**) in combination with platinum (**Extended Data Fig. 4**). The effect of combination treatment and proximity to first-line treatment means that PFS intervals in this context are likely to be heavily influenced by platinum. Thus in this case we chose to include an interaction between first-line PFS and the main predictor variable in our Cox model.

Predicting resistance to taxane treatment in relapsed ovarian - TCGA

Given the increased cohort size for TCGA, we could be more strict in our eligibility criteria to minimise the effect of platinum treatment. Therefore, we did not consider any patients that received taxane in combination with platinum. The majority of the remaining patients received taxane treatment second-line (**Supplementary Note Fig. 2c**). This proximity to first-line treatment means that TTF intervals in this context are likely to be influenced by platinum. Thus in this case we chose to include an interaction between first-line TTF and the treatment arm variable in our Cox model.

Predicting resistance to anthracycline treatment in relapsed ovarian - TCGA

Similar to taxane in TCGA above, we did not consider patients who received anthracyclines in combination with platinum. In this instance, the majority of patients received treatment in lines 2, 3, or 4 (**Supplementary Note Fig. 2d**). Given the proximity to first-line treatment effect is likely weaker, we elected not to control for this effect via an interaction. Rather, we classified patients as platinum resistant (≤6 months TTF) or platinum sensitive (>6 months TTF) and adjusted for this effect in the model as a covariate.

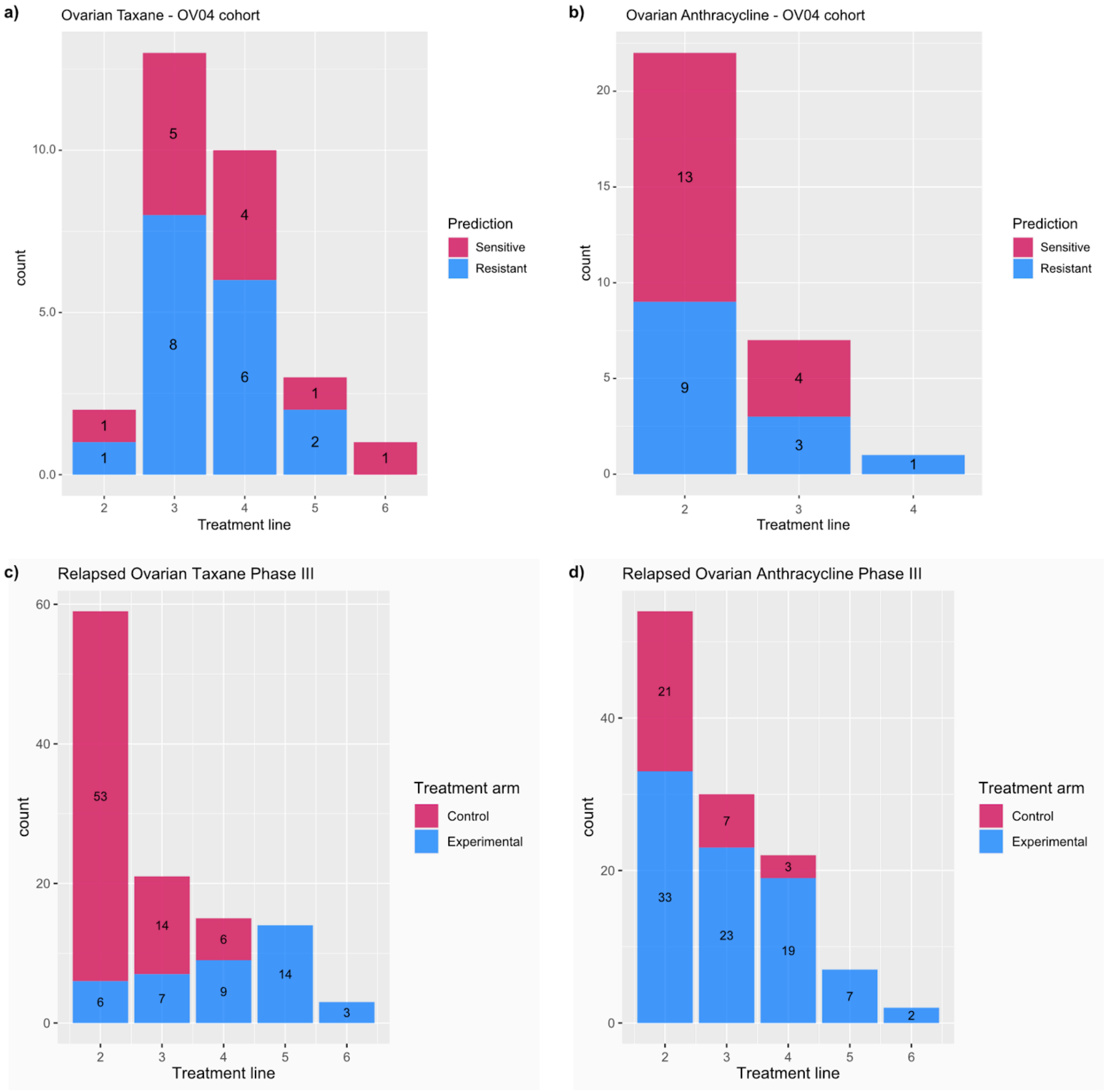

Supplementary Note Figure 2. Distribution of taxane-based and anthracycline-based treatment administration in ovarian cancer per treatment line. a) Taxane administration in the OV04 cohort. **b)** Anthracycline administration in the OV04 cohort. **c)** Administration of taxane in relapsed ovarian cancer from TCGA. **d)** Administration of anthracycline in relapsed ovarian cancer from TCGA.

Supplementary Note 3 - Assessing performance of HRDetect and Myriad myChoice

Here, we sought to evaluate the performance of two state-of-the-art HRD predictors, HRDetect and Myriad MyChoice in predicting resistance to taxane and platinum-based treatment. These HRD predictors incorporate additional data beyond copy number[8–10].

We extracted predictions from application of these two methods to ovarian and breast cancer TCGA cohorts: HRDetect annotations were taken from previous publications[8,9], and a threshold of 0.7 was used to identify positive samples[8]; Myriad myChoice annotations were based on HRD scores[10] which were taken from previous publications[11,12], and scores above or equal to 42 classified as positive.

We conducted a survival analysis comparing TTF between patients predicted as HRD-positive or negative using these methods (thus sensitive or resistant to taxanes and platinum) (**Supplementary Note Fig. 3**). Due to the limited number of samples with HRDetect and/or Myriad MyChoice information, we only were able to assess performance by a phase II single-arm trial emulation.

Out of the six emulations, only one showed a significant difference in TTF between predicted sensitive and resistant patients. HRDetect negative patients showed a significantly increased risk of treatment failure compared to sensitive platinum-based treatment in ovarian cancer (HR=3.87, p=0.01, 35/75 samples with available HRD scores; independent of tumour stage and age at diagnosis). The other results did not show predictive capacity (HRDetect in TCGA-OV taxane (n=6/42): HR=0.623 p=0.61; Myriad in TCGA-OV taxane (n=6/42): NA all are predicted positive; Myriad in TCGA-OV platin (n=35/75): HR=0.757 p=0.583; HRDetect in TCGA-BRCA taxane (n=8/75): HR=1.843, p=0.504; Myriad in TCGA-BRCA taxane (n=8/75): HR=1.843, p=0.504;). However, it is noted, in some instances these analyses were likely underpowered.

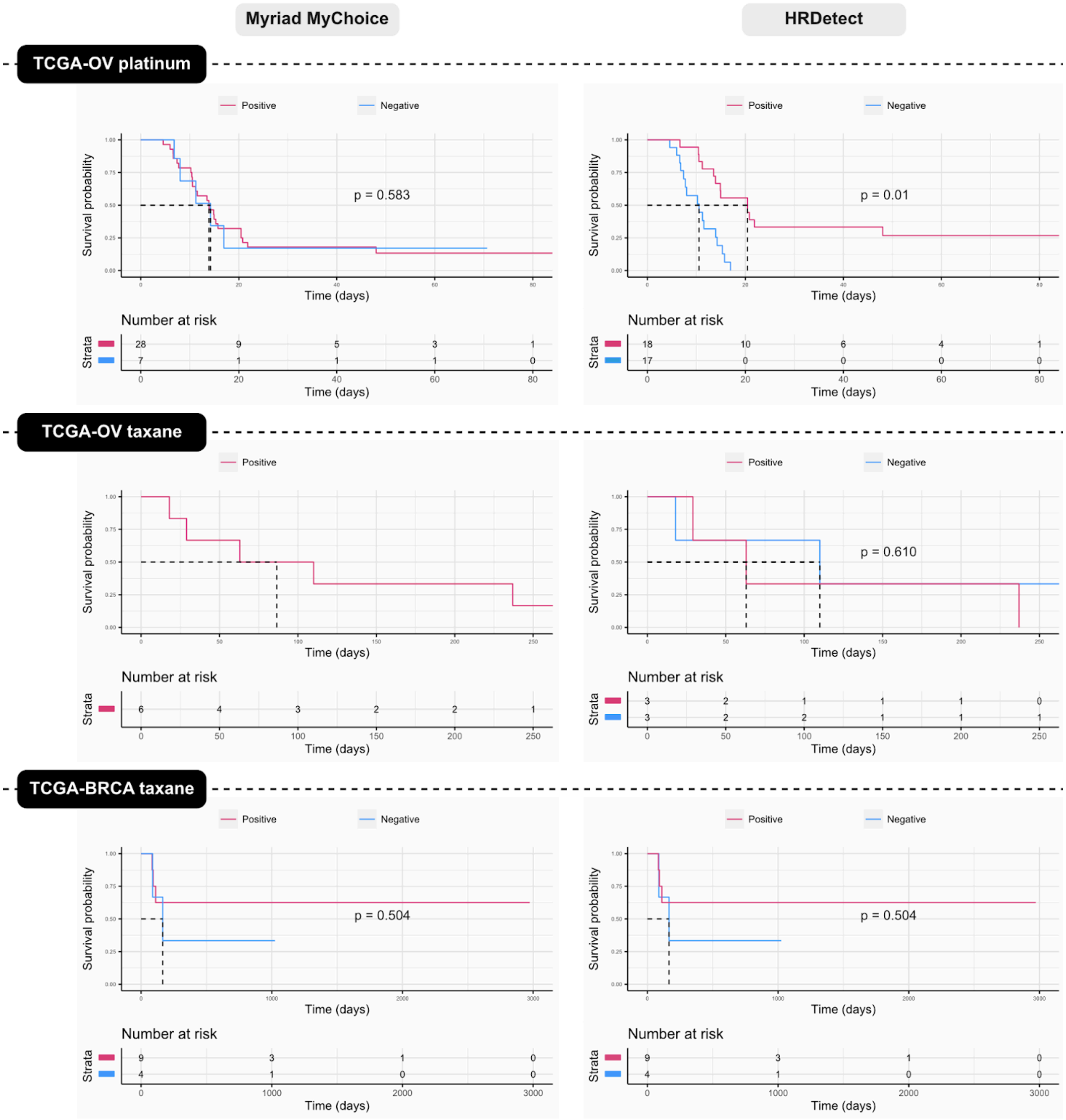

Supplementary Note Figure 3. Performance assessment of state-of-the-art HRD classifiers for predicting resistance to platinum and taxanes. Kaplan Meier curves for patients predicted to be HRD-positive (yellow) and HRD-negative (blue). HRD-positive patients are classified as sensitive, while HRD-negative patients are classified as resistant. TCGA-OV platinum: P-values from cox models corrected by tumour stage and age at diagnosis. TCGA-OV and TCGA-BRCA taxanes: P-values from univariate cox models.

Supplementary Methods

Data curation and annotation of sample cohorts

The Cambridge Translational Cancer Research Ovarian Study 04 (OV04)

Clinical data

To compile a real-world cohort from the OV04 study, we assessed patients enrolled in OV04 from 2010-2019 and identified 130 patients with available tumour samples who received platinum-based chemotherapy as a first-line therapy and/or anthracycline and/or taxane as a subsequent treatment. These patients underwent histopathological review, with 3 patients discarded for not showing signs of HGSOC pathology. Samples collected from these patients totalled 130 tumour samples and 29 cell free DNA (cfDNA) samples extracted from plasma. Following sample processing and quality control (see section “Sample and data processing”), we applied additional filtering steps based on the available clinical history data. Specifically, we excluded patients who lacked complete treatment data and ovarian cancer-related tumor marker CA125 (CA125) information, had gaps in CA125 data across different treatment lines, or did not show abnormal CA125 values during the first line of treatment. We also excluded patients with Stage I tumours as they were considered as low risk. A final cohort of 50 ovarian cancer patients was identified as suitable for retrospective assessment of treatment resistance prediction. Among them, 9 patients had high-quality plasma samples to assess prediction performance in liquid biopsies. In addition, 8 patients also had high-quality data from the Illumina TruSight Oncology 500 (TSO500) assay to assess feasibility of prediction performance using a regulatory approved assay (see **Extended Data Fig. 2-3** for sample workflow, **Supplementary Table 3** for clinical characteristics of the cohort, **Extended Data Fig. 4** for regimens of chemotherapy administration in the cohort, and **Supplementary Fig. 24-73** for treatment history of each patient).

Aside from these 50 patients, additional patient samples from the OV04 study were used to harvest spheroids from ascitic fluid, derive organoids and generate cell lines for the purpose of CIN signature biomarker discovery for anthracycline prediction[13]. A total of 8 organoids and 15 spheroids, and 4 ovarian cancer cell lines were available for downstream analyses (**Extended Data Fig. 1c**). Further characterisation of these collections of patient-derived spheroids and organoids can be found in parallel studies[13,14]. Two of the 4 ovarian cancer cell lines (CIOV2 and CIOV4) were derived in-house from OV04 patients[13,14], OVCAR3 was obtained from the American Type Culture Collection (catalogue number HTB-161), and PEO23 was a kind gift from Langdon lab.

A total of 45 patients treated with platinum first-line were used for the pilot study assessing platinum resistance prediction (**Extended Data Fig. 2a**).

For taxane resistance prediction, a subset of the 45 platinum-treated ovarian patients were used that had been treated with taxanes after first-line treatment. An additional 6 patients with missing data at first-line, but taxane treatment data after first-line were included. This resulted in a group of 29 patients, of which 4 also had plasma samples with a sufficiently high ctDNA fraction and 3 also had high-quality TSO500 data (**Extended Data Fig. 2b**).

For anthracycline resistance prediction, the clinical histories of the 45 platinum-treated patients were further inspected to identify those also given anthracycline as a subsequent treatment. Patients with 3 or fewer cycles of anthracycline were excluded as they did not receive sufficient treatment to reliably impact clinical outcomes. Additionally, patients who received anthracycline in combination with platinum-based chemotherapy at first-line were removed, resulting in 36 out of the 45 patients suitable for the retrospective validation of anthracycline prediction. Among them, plasma samples with high ctDNA fraction were available for 5 patients, while high-quality TSO500 data was available for 3 patients (**Extended Data Fig. 2c**).

Organoids and spheroids

Samples showing greater than 20% standard deviation across more than 3 dose concentrations were removed from downstream analysis (see section “Clinical classifier of anthracycline resistance” in **Supplementary Methods** for further details of doxorubicin treatment assays).

Patient tissue samples

Copy number from sWGS: Tissue samples with absolute copy number profiles were evaluated using a manual curation where samples were rated using a 1-3 star system, following the criteria from our previous work[15]. This star rating system is designed to evaluate the copy number fits for their ability to produce reliable CIN signature activities. The description of the different star ratings is as follows:

- 1-star: samples show some observed copy number change, but not enough to provide a reliable CIN signature activity. They can be used in some analysis if the size of the cohort outweighs the lower reliability of the signatures.
- 2-star: samples have clear copy number fits, but can be underpowered, or are too noisy to be 3 star.
- 3-star: samples have ideal copy number fits, minimal noise, and are not underpowered.

Following absolute copy number fitting, samples were rated using this star system. Overall 33 samples were identified as 1-star and were subsequently excluded from downstream analysis. These 1-star samples showed noisy copy number profiles and were considered likely to have incorrect segments and missing calls. In general, only 2 and 3-star copy number profiles are usable in downstream analysis, resulting in 102 out of 135 primary samples (75.56%) being usable for signature analysis. Overall this is in line with typical sequencing success rates for archival material[16].

Copy number from TSO500: Tissue samples with less than 40% purity were excluded for downstream analysis.

Plasma samples

ctDNA Frequency: Samples were curated according to their copy number profiles, where samples with low levels of circulating tumour DNA displayed insufficient copy number alterations. These samples were excluded from the analysis as the estimation of the copy number signatures is not accurate. This resulted in 9 out of 29 (31%) being usable for signature analysis.

The Cancer Genome Atlas collection

Manually curated clinical data was available for the majority of TCGA ovarian cancer patients (TCGA-OV)[17]. This clinical dataset focused specifically on high-risk HGSOC (Grade 3, Stage III/IV). For the remaining TCGA cancer types, we curated clinical datasets using data from the data portal of the Genomic Data Commons (GDC)[18], following the approach used for ovarian cancer[17].

Each patient's clinical history was downloaded from GDC in the form of an XML, from which we collected overall survival, date of last follow-up, tumour stage, tumour grade, age at diagnosis, tissue source site, the number of cycles of administered treatment and the dates of tumour events. Following this, the treatment history was reconstructed. Clinical characteristics of each cohort can be found in **Supplementary Table 5**.

Treatment history information

Each individual treatment is listed separately in the XML, usually with the start and end treatment dates. We also extracted (if available) the number of treatment cycles. To address data inconsistencies, therapy names for platins, taxanes, anthracyclines, and other common treatments were standardised.

Treatments were sorted by start date, and overlapping treatments were merged into a single treatment line. The start and end dates for this new combined treatment line were the earliest of the individual treatment start dates, and the latest of the end dates. The cycle count was set to the minimum among merged treatments for conservative filtering.

In some cases, the XMLs also contained 'new tumour events' like distant metastases and local recurrences, which were extracted for time to treatment failure (TTF) calculation. This process resulted in a total of 7,105 clinically-annotated tumours from the TCGA cohort.

To create a cohort for a given cancer type and chemotherapy of interest (platinum/taxane/anthracycline), we first selected TCGA patients of the specific cancer type. We then excluded treatment lines without calculable TTF and Stage I patients. For breast cancer patients, patients who received anti-HER2 treatment were also excluded, as these therapies significantly improve survival time compared to other treatments, and therefore could overshadow biomarker efficacy. HER2 inhibitors were also omitted from the control arm due to their superior performance[19–24].

Annotation of breast tumour subtypes

The TCGA breast cancer (TCGA-BRCA) cohort underwent annotation for triple-negative status, estrogen receptor (ER) and progesterone receptor (PR) expression, and human epidermal growth factor receptor (HER2) amplification. Triple-negative status was annotated using three distinct datasets incorporating RNA and microRNA expression data, histologic analysis, mutation, copy number, epigenetic, proteomic, and phospho-proteomic information[25–28]. Tumours were classified as triple-negative breast cancers (TNBC) only if all three datasets agreed on this annotation. ER, PR, and HER2 status were annotated using two additional datasets[25,26], requiring agreement between both for a positive classification.

Annotation of BRCA mutation status

We used data collated in our previous work[29] to annotate TCGA samples as *BRCA1/2* mutants based on germline and somatic loss-of-function mutations, loss of heterozygosity (LOH), and promoter hypermethylation status.

The Hartwig Medical Foundation dataset

All patients in the HMF dataset were enrolled at the time they presented their first metastatic tumour. Clinical data provided was organised into 4 tables with the following information: pre-biopsy treatment, post-biopsy treatment, treatment responses, and patient metadata. Pre-biopsy treatment table covers the patients' clinical history prior to their enrollment into the HMF dataset, which is acknowledged to be less accurate and comprehensive than the post-biopsy treatment table. Pre-biopsy treatments are grouped at patient level, whereas post-biopsy treatments are grouped at biopsy level. These two tables provide information on the therapies administered, and the start and end treatment dates. The table with treatment response information includes the dates of target lesions evaluations based on response evaluation criteria in solid tumours (RECIST) criteria, while the metadata table includes date of death, cancer type and subtype for each HMF patient.

Patients without post-biopsy treatment were excluded. Pre- and post-biopsy treatment tables were merged, linking treatments to the most recent biopsy ID for accurate signature activities quantification and subsequent survival analysis. Treatments with missing or incomplete data were then removed. Pre- and post-biopsy treatments were combined into treatment lines based on overlapping dates. Treatments were combined if the start date of the second treatment was lower than the end date of the first treatment. However, one-day treatments were merged into the same line if they occurred on the same day that the previous treatment ended. This process resulted in a total of 7,437 clinically-annotated tumours from the HMF cohort (**Supplementary Table 5**).

Annotation of weighted Genome Instability Index (wGII)

wGII (a measure of aneuploidy) was computed for each tumour sample as the percentage of the genome altered by copy number events:

$$wGII = \left(\sum_{i=1}^{N} \frac{L_i}{3e9} \right) \times 100$$

where:

L_i is the length of the non-diploid segment i

N is the total number of non-diploid segments in the sample

$3e9$ is the approximate length of the human genome

Biomarker development and optimisation

Clinical classifier of platinum resistance

Platinum resistance prediction was performed using a classifier based on the CX3 and CX2 signatures, which was prototyped in Drews *et al*[29] where, after scaling signature activity levels to a reference cohort, a sample is predicted to be platinum resistant when the CX2 activity is greater than CX3 activity, otherwise sensitive. In addition, samples without detectable CIN are considered as resistant. In this original classifier, 36 germline *BRCA1* mutant samples of the TCGA-OV cohort that had an additional loss of heterozygosity at the *BRCA1* locus as reference cohort were used for the scaling procedure. Consequently, when applying this classifier to new samples, their CX2 and CX3 signature activities were assessed relative to this reference cohort. While this method was effective for ovarian and breast cancer samples, it was not suitable for oesophageal samples[29]. Here, we extended the clinical classifier for platinum resistance across multiple cancer types by using 375 TCGA samples with germline and/or somatic mutations in *BRCA1/2* as the reference cohort. This pan-cancer scaling and centering procedure improved prediction results for oesophageal cancer compared to the original method (**Fig. 1b**). Both approaches showed similar classifier performance for ovarian and breast cancers.

Clinical classifier of taxane resistance

By correlating signature activities, gene essentiality scores, and sensitivity to drug perturbation across 297 cancer cell lines from the DepMap project[30], we previously identified impaired homologous recombination (IHR) signatures CX3 and CX5 were significantly correlated with resistance to paclitaxel[29]. To build a clinical classifier, a linear model for predicting paclitaxel resistance including both signatures was initially generated, observing that CX3 and CX5 had an independent effect on the area under dose response curve (AUC) value for paclitaxel. Given that CX5 presented a higher correlation coefficient than CX3, we finally selected CX5 for building a binary classifier to predict patients as sensitive or resistant to paclitaxel (**Supplementary Table 1**). To construct a pan-cancer clinical classifier of taxane resistance, we used 287 cancer cell lines treated with paclitaxel[31]. Using the entire TCGA cohort as reference, we first centred the mean and scaled the variance of the CX5 signature activities across cell lines. We then identified the most effective threshold for optimising the prediction of paclitaxel response by comparing paclitaxel response (AUC values) between cell lines predicted as sensitive or resistant using a t-test. After exploring values from -0.1 to 0.1 in increments of 0.01,

we selected 0 as the optimal threshold of CX5 signature activity to classify a tumour sample as resistant. We selected this optimal threshold because: 1) significant differences in AUC values at significant level of 10% were observed between cells predicted as resistant and sensitive (t-test p-value = 0.062; **Supplementary Fig. 1**); 2) the mean AUC values of resistant cells at different activity values stabilised; 3) the rate of cells predicted as resistant aligned with the expected resistant rate in the literature[32]; and 4) this threshold represents the limit for positive signature activities at a cohort level. The application of this classifier to a new tumour sample implies first a scaling and centering of its CX5 activity relative to the TCGA cohort, and second to classify as resistant if CX5 activity is lower than the optimal threshold. As with the platinum biomarker, samples without CIN are also considered resistant.

Clinical classifier of anthracycline resistance

In our endeavour to use CIN signatures as biomarkers for predicting doxorubicin resistance, we initially aimed at using genomic and drug response data obtained from the 297 cancer cell lines that were used to identify CX5 as a biomarker for taxane prediction (see section "Clinical classifier of taxane resistance"). However, we encountered limitations in the range of AUC values (0.58 ± 0.08) for doxorubicin response across the cell lines, which impeded the possibility of achieving accurate correlations. Furthermore, there was a lack of sufficient cell lines exhibiting AUC values close to 0, and therefore sensitive to this treatment. For this reason, we designed *in vitro* experiments to identify and validate a signature-base anthracycline resistance predictor (see **Supplementary Note 1**).

Doxorubicin treatment assays

Treatment of cell lines

Cancer cell lines were initially exposed to four doxorubicin concentrations (0, 0.1, 0.5 and 1µM) for 5 days. Since cell growth rates significantly decayed after one day of exposure to all concentrations tested (**Supplementary Fig. 79**), we then performed cell viability assays by treating cells with 0.025µM and 0.05µM for 48 hours. Two cell lines showed 100% confluence after 48 hours of being exposed to low doses of doxorubicin and therefore were discarded from further analyses due to signal saturation (**Supplementary Fig. 80**). All the remaining cell lines (OVCAR3, PEO23, CIOV2 and CIOV4) showed stable growth rates in the presence of 0.025µM of doxorubicin, and were therefore used for evaluating micronuclei formation and tolerance. For doxorubicin treatment and micronuclei formation experiments, optical 94-well plates (CellCarrier-96 Ultra, Perkin Elmer) were coated with sterile Poly-L-Lysine (P4832, Sigma), and cells were seeded using previously estimated seeding densities until 50% confluency was reached. Subsequently, cells were treated with 0.025, 0.05 and 0.1µM of doxorubicin or control for 5 days. All treatment experiments were performed in triplicates.

Treatment of spheroids and organoids

For organoid and spheroid samples doxorubicin treatment was administered following the method in Martins *et al*[14]. Briefly, an 8-point half-log dilution series of doxorubicin starting at 30µM was dispensed into 384 well plates using an Echo® 550 acoustic liquid handler

instrument (Labcyte) and kept at -20°C until used. Organoid plates were spun down and 50 µl of suspension added per well using a Multidrop™ Combi Reagent Dispenser (Thermo-Fisher). Following 5 days of drug incubation cell viability was assayed using 30µl of CellTiter-Glo® (Promega). Screens were performed in technical triplicate. An untreated control was used to normalise response values to be equivalent to the percentage of viable cells remaining. To assist with dose response curve fitting, dummy values were added for each sample below and above the minimum and maximum dose ranges at 100% viable cells with a dose of 1e-3µM and 0% viable cells at a dose of 300µM. A 4 parameter log-logistic model was used to fit dose response curves, which includes the IC50 parameter used here. Fitting was performed using the *drm* function in the *drc* package in R[33]. For spheroid samples, the IC50 values were scaled by the inverse of the tumour purity to account for increased cell viability due to normal cell contamination.

We then determined an IC50 threshold which divided the samples into those considered sensitive or resistant based on the clinical characteristics of the patients. As our *in vitro* drug screen is analogous to patients being treated with doxorubicin as a monotherapy following first line treatment with platinum based chemotherapy, we estimated the expected number of sensitive samples based on response observed in clinical trials. Patients resistant to platinum chemotherapy are expected to have an 18% response rate to doxorubicin monotherapy[34–40], and sensitive patients a 28% response rate[41]. Patients who had relapsed disease less than 6 months after first-line platinum-based chemotherapy were considered resistant and greater than 6 months sensitive (**Supplementary Table 2**). These data allowed us to estimate the expected number of doxorubicin sensitive organoids to be approximately 2 (5 x 0.28 + 5 x 0.18 = 2.3) and the sensitive spheroids to be 4 (8 x 0.28 + 7 x 0.18 = 3.5). Samples were ranked based on their IC50 and a threshold set yielding the expected number of sensitive samples in each case.

Micronuclei counting

Micronuclei counts were estimated using fluorescent imaging as previously described[42]. In brief, following doxorubicin treatment, cells were fixed in 100% ice-cold methanol for 5 mins at -20°C, washed three times with 1xPBS and permeabilised with 1% TritonX100 + 0.5% NP40 in 1xPBS for 5 mins at room temperature, before blocking for 1 hour with 5% BSA in 1xPBS at room temperature. To allow reliable micronuclei identification at single cell level, cells were stained with Hoechst (1µg/ml; Hoechst 33342), phospho-histo H3 (pHH3; 1µg/ml; 06-570 Merck), and cytokeratin 7 conjugated to fluorophore 488 (1µg/ml; ab208273 Abcam). Primary non-conjugated antibodies were diluted in blocking buffer, added to the cell lines and incubated overnight at 4°C. Cells were washed three times with 0.1% Tween 20 in PBS, and secondary antibody (Alexa Fluor 555, 1µg/ml; A-21429 Invitrogen) diluted in blocking buffer was added to the cells for 1 hour at 37°C. Hoechst 33342 was diluted to 1 µg/ml in MilliQ water, added to the cells and incubated for 15-30 mins at room temperature. Cells were washed and stored in PBS.

Stained cell lines were imaged in filtered 1xPBS as mounting medium (200 µl/well) with a confocal 40x 1.1NA water objective using the Operetta CLS™ high-content analysis system. Images from ten independent non-overlapping imaging fields were acquired from each well (three wells per cell line and treatment group), with Z-stack images being collected at a step

size of 0.5 μm (28 planes in total) across all imaging channels (i.e. 405 nm, 488 nm and 555 nm wavelengths).

Image analysis was performed using the Harmony 4.9 software. Images were reconstructed as maximum projections using basic brightfield correction, and analysed by performing nuclei segmentation, removing border object to only include whole cells, identifying cytoplasm and regions of interest using cytokeratin stains and performing spot counting and micronuclei identification using the Harmony 4.9 Micronucleus Analysis RMS module. Mitotic cells identified via pHH3 staining were excluded from micronuclei counting analysis as condensed chromosomes were commonly misidentified as micronuclei. In addition pHH3 stains were used to estimate the mitotic index of the treated and stained cell lines. The fraction of cells with micronuclei in cell lines after being exposed to doxorubicin was computed by taking into account the number of mitotic cells which have doubled their genetic material. We then compared the observed and the expected fraction of micronucleated cells in each cell line in order to evaluate if the cell lines with amplification-related signatures (CX8/CX9/CX13) have a lower fraction of micronucleated cells than expected as a sign of doxorubicin resistance.

Estimating expected number of micronuclei

To perform statistical analyses, we then modelled the expected fraction of cells with micronuclei across divisions in culture under the presence or absence of doxorubicin. The model considered the fraction of dividing cells in each cell line after 48 hours in culture with or without 0.025μM of doxorubicin (f), the fraction of induced micronuclei persisting across cell divisions (p=75%[2]), and the fraction of cells hit by doxorubicin (d=60%[43]) or experiencing damage under normal culture conditions (d=25%[43]).

This approach for estimating the fraction of micronucleated cells was based on a previous study that monitored micronucleus-containing cells through time-lapse imaging[2]. This study observed an increase in the number of micronuclei per cell after the first division, indicating that some cells contained more than one micronuclei, ideally ensuring that each daughter cell possessed at least one micronucleus. However, subsequent generations exhibited a decrease in the number of micronuclei per cell, suggesting that some cells lost or reintegrated their micronuclei. This phenomenon of micronuclei persistence occurred in approximately 75% of cases.

Furthermore, we also considered cell viability in the model. Micronuclei persisted over time and generations in non-dividing cells, while the number of micronucleated cells was expected to reduce by 50% with each cell division. In the first division, where there was no degradation, reincorporation, or extrusion of micronuclei, the estimated rate of cells taking up doxorubicin (and consequently being damaged by the drug) was approximately 60%[43]. Under control conditions, the rate of cells accumulating damage was approximately 25%[43]. Consequently, the formation of micronuclei would only be induced in this fraction of cells.

Taking altogether, we estimated the number of micronucleated cells after the first division (MN_t) as follows:

$$MNt = (1 - f) \times MNi + d \times f \times \left(\frac{MNi}{2} + MNi + \frac{1 - MNi}{2}\right)$$

where MN_i is the number of micronuclei observed at time 0 in culture. In case the fraction of dividing cells (*f*) was higher than 1, we corrected the number of micronucleated cells across subsequent divisions as follows:

$$MNt = (1 - f) \times MNt + p \times f \times \frac{MNt}{2}$$

The fraction of dividing cells was corrected across divisions by subtracting 1.

Optimal threshold selection *in vitro*

To identify the most effective threshold for optimising the prediction of anthracycline response, we used drug response data obtained from a total of 23 patient-derived ovarian cancer models (15 spheroids and 8 organoids). We conducted a grid search, varying the activities from 0.005 to 0.015 in increments of 0.001, exploring all possible combinations of the three amplification-related CIN signatures (CX8, CX9 and CX13). Performance of each combination of activities was assessed using specificity and sensitivity for predicting resistance to anthracycline. Sensitivity was considered as the proportion of sensitive samples correctly identified and specificity as the proportion of resistant samples correctly identified. We then selected combinations that achieved both 100% specificity and the highest sensitivity. Subsequently, we applied these selected activity combinations to the tumours data of the OV04 cohort to determine the optimal combination for predicting anthracycline resistance. Thresholds of CX8>0.01, CX9>0.009 and CX13>0.009 showed optimal classification of the patient-derived models and tumour samples. Therefore, samples with an activity level greater than 0.01 in CX8, or greater than 0.09 in CX9 or CX13 were classified as anthracycline resistant, while samples falling below this threshold were predicted to be sensitive to anthracycline. In this case, patients without detectable CIN were classified as sensitive.

Gene enrichment analysis

To further explore the mechanism driving micronuclei tolerance in resistant tumours, we performed a differential expression analysis between ovarian TCGA tumours predicted as resistant or sensitive to anthracycline based on our clinical classifier by using the *DESeq2* package in R[44]. Genes with low overall expression counts (sum counts across all samples < 10) were excluded. Genes that exhibited significant expression differences across the two groups were ranked based on their significance and fold changes to facilitate. For this analysis, we selected “h.all.v7.1.symbols.gmt” as the reference set, the number of permutations was set to 10,000, and a minimum of 15 genes was required for each gene set. Gene sets with an FDR-adjusted p-value below 0.05 were considered for further analyses, and the Normalised Enrichment Score (NES) was used to indicate the level of enrichment within the gene sets.

Survival period calculation

Progression-free survival (PFS)

PFS for platinum treatment in OV04

PFS was calculated following the CA125 definitions of progression in first-line therapy agreed by the Gynecologic Cancer InterGroup (GCIG) in November 2005. Patients were sorted into three separate categories based on their CA125 levels during first-line platinum treatment following National Institute for Health and Care Excellence (NICE) guidance: Category A, patients with abnormal initial readings that normalised during treatment; Category B, patients with abnormal initial readings that never normalised; and Category C, patients with normal initial readings. In these definitions, the normal range is 0-35 CA125, and the abnormal range is 35+. These categories then defined the patient-specific CA125 progression threshold. In categories A and C the progression threshold was twice the normal limit (70), and in category B the threshold was twice the value of the lowest CA125 reading in the treatment line. In all cases, progression was only confirmed if there was a second reading above the threshold that occurred at least one week after the first reading. To avoid false early progressions, CA125 readings that occurred while treatment was being actively administered were not considered for progression. If there were no readings that met the criteria for progression within the treatment line, the progression date was set as the beginning of the next line. PFS was then defined as the number of days from the date of diagnosis to the date of progression.

PFS for anthracycline and taxane treatment in OV04

The start of the progression-free survival interval was considered as the date of the first treatment cycle. If the patient responded to treatment, progression was calculated using the same criteria as for platinum, but was allowed to occur during treatment, starting after the 3rd cycle, due to limited post-treatment readings. For non-responding patients, the progression date was set as the start of the following treatment line. Response was identified according to CA125 levels, following amended GCIG guidelines. A response was considered if there was at least a 50% reduction in CA125 levels from a pretreatment reading that was at least twice the upper normal limit, which may be taken within 14 days before the first chemotherapy cycle. This 50% reduction had to be maintained for at least 28 days, with subsequent readings either lower than the previous or no more than 10% higher. However, these guidelines were relaxed to account for the occasionally infrequent timing of CA125 readings within the cohort. We allowed the pretreatment reading to be taken slightly earlier than 14 days before the first treatment, or slightly after, to account for the lag between therapy and changes in CA125 levels. If a patient showed a drop below 50% but no follow-up reading for at least 2 months to confirm the decrease, they were still considered a responder. All patients meeting these criteria were reviewed by a clinician to validate our approach. If the criteria were not met before the next treatment cycle, the patient was deemed non-responder. Finally, patients without a pretreatment CA125 value to calculate response were considered responders. After the progression date was calculated, progression-free survival (PFS) was then calculated as the number of days from treatment start to progression.

Time to Treatment Failure (TTF)

Due to limited information regarding cancer progression in the TCGA and HMF clinical data, TTF was used as a proxy for PFS.

TCGA

TTF was calculated as the time from treatment initiation to the initiation of the subsequent treatment, unless disease progression (i.e. metastasis/relapse) was reported. For adjuvant treatments, treatment lines were manually combined. The TTF data for the TCGA-OV cohort was previously curated[17]. We followed the same approach to calculate TTF in the other tumour types. For the final treatment lines, TTF cannot be calculated using a subsequent treatment, rather we used the date of the next tumour event. If no new event occurred, TTF was based on death date or last follow-up, or ultimately we used the treatment length. Finally, treatment lines were censored if they were calculated from the last follow-up or treatment end date.

HMF

TTF was calculated as the time between the start of one treatment line and its subsequent line. For the final treatment line, TTF was based on the death date; if unavailable, RECIST response data was used. If a response date occurred after treatment started, the latest response date was chosen, regardless of progression, stability, or other outcomes. In this case the RECIST data was not used to calculate response, instead being used as a proxy for the date of last follow up. If no death or response date was available, TTF was calculated based on the treatment line length. Treatment lines were censored if calculated using RECIST data or treatment duration. Note: Due to insufficient RECIST response data, we could not compute PFS data for all patients and/or treatment lines. Additionally, the long follow-up between CT/MRI scans introduced noise, so we decided to use RECIST-based data only to calculate TTF for the final treatment line.

Biomarker performance evaluation

Power Analysis

All possible tumour type and chemotherapy combinations were subjected to power analysis following the Consolidated Standards of Reporting Trials (CONSORT) statement[45]. Only cohorts predicted to be sufficiently powered were considered for clinical assessment of our biomarkers.

The use of retrospective real-world data prevented us from conducting a traditional power calculation approach, as we lacked access to information such as the length of the accrual period, length of follow-up, and dropout rate. Therefore, we used the resulting hazard ratios obtained in our proof-of-concept study in the OV04 cohort to guide the power analysis in the pan-cancer primary and metastatic cohorts. To identify the required cohort size to have sufficient power, we adapted a previous approach[46] as follows:

$$D = (Z_{1-\alpha} + Z_{1-\beta})^2 / (P(1 - R^2)\sigma^2 B^2)$$

where:

D is the required number of patients

$Z_{1-\alpha}$ and $Z_{1-\beta}$ are the standard normal deviates at the desired significance level α and power $1 - \beta$.

P is the proportion of patients that recorded an event within the recorded clinical history

R^2 is the squared multiple-correlation coefficient between the predictor and the remaining covariates.

σ^2 is the variance of the predictor in terms of the number of patients predicted resistant or sensitive

B is the log hazards ratio of survival between the predicted resistant and predicted sensitive patients, also known as the effect size.

In all cases, we set α to be 0.05 for a 1-tailed analysis, and the power as 0.8 following standard clinical trial practice. Similarly, we set R^2 at 0.05. Finally, the log hazards ratio *B* was taken from the results of the ovarian pilot study. Given these parameters, we determined the required number of patients for the different available tumour-type primary and metastatic cohorts (**Extended Data Tables 1-2, Supplementary Table 4**).

Phase II single-arm trial emulation

We retrospectively emulated phase II single-arm biomarker studies using the design outlined in **Extended Fig. 5a**, where patients who receive the same treatment are stratified into biomarker positive and negative groups[47].

Cox proportional hazards models were used to compare the PFS between ovarian cancer patients classified as resistant or sensitive included in the OV04 study (**Fig. 2**). Models were corrected for tumour stage, age at diagnosis, treatment line (for taxane), general aneuploidy (via wGII) and whether or not the patient received maintenance therapy (during first-line treatment, or prior to treatment with taxane or anthracycline). Maintenance therapies were defined as either niraparib, olaparib, bevacizumab or letrozole. For taxane and anthracycline treatments, survival analyses were also controlled by the sensitivity to 1st-line platinum treatment (see further details in **Supplementary Note 2**). Swimmer plots illustrated periods on treatment, follow-up observations, and PFS in one glance (**Supplementary Fig. 2-4**). Wilcoxon rank sum test was used to compare time on therapy between non-censored patients predicted to be resistant or sensitive.

Phase II emulation was also performed for primary ovarian cancer treated with platinum and sarcoma treated with anthracyclines. These cohorts had sufficient sample sizes for emulating Phase II single-arm trials, but not for Phase III randomised controlled study emulations (**Extended Data Table 2**). Cox proportional hazard models were used to compare the TTF

between patients predicted as resistant or sensitive to the chemotherapy of interest. In ovarian cancer, a stratified Cox proportional hazard model was applied to account for different baseline hazards across age at diagnosis groups (<65 or ≥65 years). Survival analysis was corrected by tumour stage (III, IV). In the case of sarcoma, the model was controlled for the presence of isophosphamide as a co-therapy. Tumour stage annotation was not available for this cohort, so it was not included in the analysis.

Phase III randomised controlled study emulation

We conducted a survival analysis that replicated a biomarker-stratified Phase III randomised controlled study design in all tumour-specific cohorts defined as powered (**Extended Fig. 5b-c, Extended Data Table 1**). This trial design, also known as a marker-by-treatment interaction design, involves dividing the patient population based on biomarker status and evaluating whether the treatment effect in one group differs significantly from the treatment effect in the other group[48].

Initially, we applied our signature-based classifiers to identify resistant and sensitive patients for a given chemotherapy. Subsequently, patients belonging to the predicted resistant and sensitive groups were retrospectively assigned to either the experimental arm (receiving the chemotherapy of interest) or the control arm (receiving alternate standard-of-care therapies). Inclusion and exclusion criteria for each arm is described in the section “Data curation and annotation of sample cohorts”.

Cox proportional hazards models were used to compare the survival period between the experimental and the control arms in both the predicted resistant and predicted sensitive groups. TTF was used as the primary endpoint. When a covariate did not satisfy the proportional hazards assumption, we applied a stratified Cox model assuming different baseline hazards for each strata. In cases where interaction terms were introduced as covariates, the resulting hazard ratio was estimated using the *intEST* function from the *interactionRCS* package in R[7]. Inverse probability weighting was applied to account for treatment selection bias due to the treatment/biopsy year across patients (see section “Inverse probability weighting”). When powered, we also performed survival analysis limiting to single-agent administration of the chemotherapy.

Enrichment trial design emulation

In the case when only the biomarker-positive arm of the phase III RCT designs was sufficiently powered to perform survival analysis, we instead replicated an enrichment trial design. This emulation follows the phase III RCT design closely, dividing the patient population according to biomarker status and evaluating whether there is a significant difference in survival times between the patients who received the chemotherapy and the patients who received an alternate standard of care, only in the patient subpopulation who were biomarker-positive[49]. The patients who were predicted to be sensitive to the chemotherapy of interest were not used for survival analysis. As with the Phase III RCT, the enrichment trial design was only used for

cohorts with sufficient sample size in the predicted resistant subpopulation (**Extended Data Table 1**).

Cox proportional hazards models were used to compare the survival period between the experimental and the control arms in the predicted resistant group. TTF was used as the primary endpoint. Inverse probability weighting was applied to account for treatment selection bias due to the treatment/biopsy year across patients (see section “Inverse probability weighting”).

Inverse Probability Weighting

Treatment selection may be influenced by patient characteristics and clinical factors[50]. To effectively emulate the randomisation process in the phase III RCT and enrichment trial survival analyses, we applied inverse probability weighting to address systemic biases that might have influenced treatment selection[51].

First, we explored the available covariates in the TCGA and HMF datasets to identify those that may have a significant effect on treatment assignments. We identified the year of treatment/biopsy as the clinical covariate most likely to impact whether a patient received the chemotherapy of interest or an alternative standard of care (range of treatment years in TCGA: 1992-2009; range of biopsy years in Hartwig: 2012-2020). This was based on the fact that treatment decisions are guided by clinical guidelines, which evolve over time. While age at diagnosis and treatment line were also considered as potential factors influencing treatment selection, we reasoned that these variables might have a stronger direct impact on survival outcomes and were instead included as covariates in the Cox proportional hazards models. We also evaluated whether geography, as well as patient race, could contribute to treatment selection bias. In the TCGA cohorts, the majority of patients were from North America, with over 95% enrolled in the USA. Similarly, all patients in the HMF cohorts were recruited from the Netherlands. Based on these observations, we concluded that geography was unlikely to influence treatment selection significantly. Regarding reported patient race, we determined that the impact of this bias would be minimal, as the patient demographics from TCGA only included 4-12% non-white individuals, split over multiple groups. Consequently, we decided to not include race as a variable in the probability weighting.

To implement the weighting in the survival analyses, we fitted a logistic regression model to examine the association between the treatment/biopsy year and the treatment arm. The model predicted the probability of each patient belonging to the experimental arm, and thus estimated propensity scores. These scores were converted into inverse probability weights using average treatment effect for the treated (ATT) as the estimand. This process was performed using the *WeightIt* package in R[52]. The resulting weights were then incorporated into the Cox proportional hazards models through the *weights* parameter in the *coxph* function.

Performance comparison with alternative metrics

HRDetect and Myriad myChoice classifier

We compared survival periods between samples classified as positive or negative by two different homologous recombination deficiency (HRD) predictions, using TTF as an endpoint (see **Supplementary Note 3** for further details).

Copy number signature activities

We tested the individual performance capacity of each of the 17 CIN signatures[29] by applying a univariate cox proportional hazards model to predict survival periods based on signature activities. No CIN signature activity showed significant association with PFS after multiple testing correction in the OV04 cohort for any of the three chemotherapies tested (**Supplementary Table 6**).

Copy number feature components

We also tested the performance capacity of the different copy number feature components defining the encoding space of our compendium signatures[29] by applying a univariate cox proportional hazards model to predict survival periods based on the sum-of-posteriors per feature component. No copy number feature components showed significant association with PFS after multiple testing correction in the OV04 cohort for any of the three chemotherapies tested (**Supplementary Table 6**).

Supplementary Figures

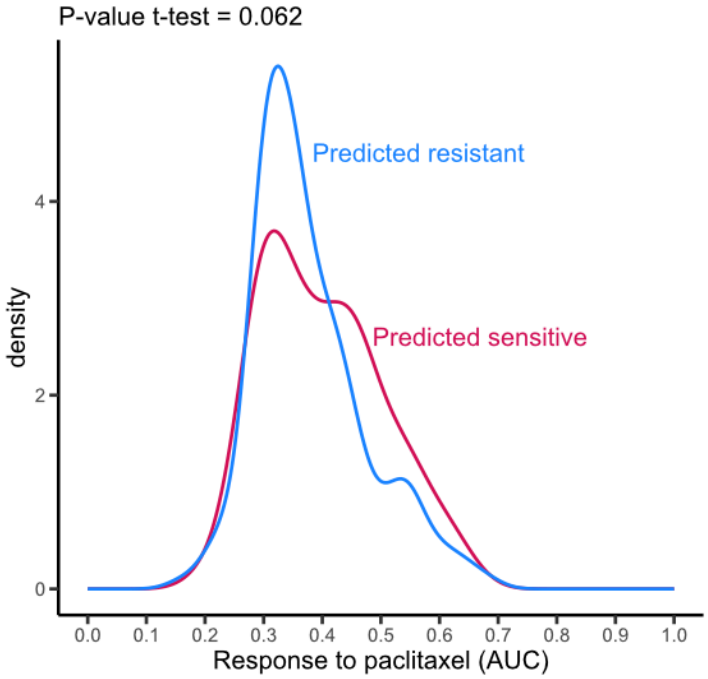

Supplementary Figure 1. Drug response in cancer cell lines predicted as sensitive and resistant to paclitaxel. Density plot showing distribution of AUC values in cancer cell lines with chromosomal instability and response data to paclitaxel in the DepMap project (n=287). Activity levels of CX5 were used to classify samples as resistant or sensitive based on the optimal threshold trained in the cohort. P-value t-test denotes significant differences at a significant level of 10% in paclitaxel response between sensitive and resistant cell lines.

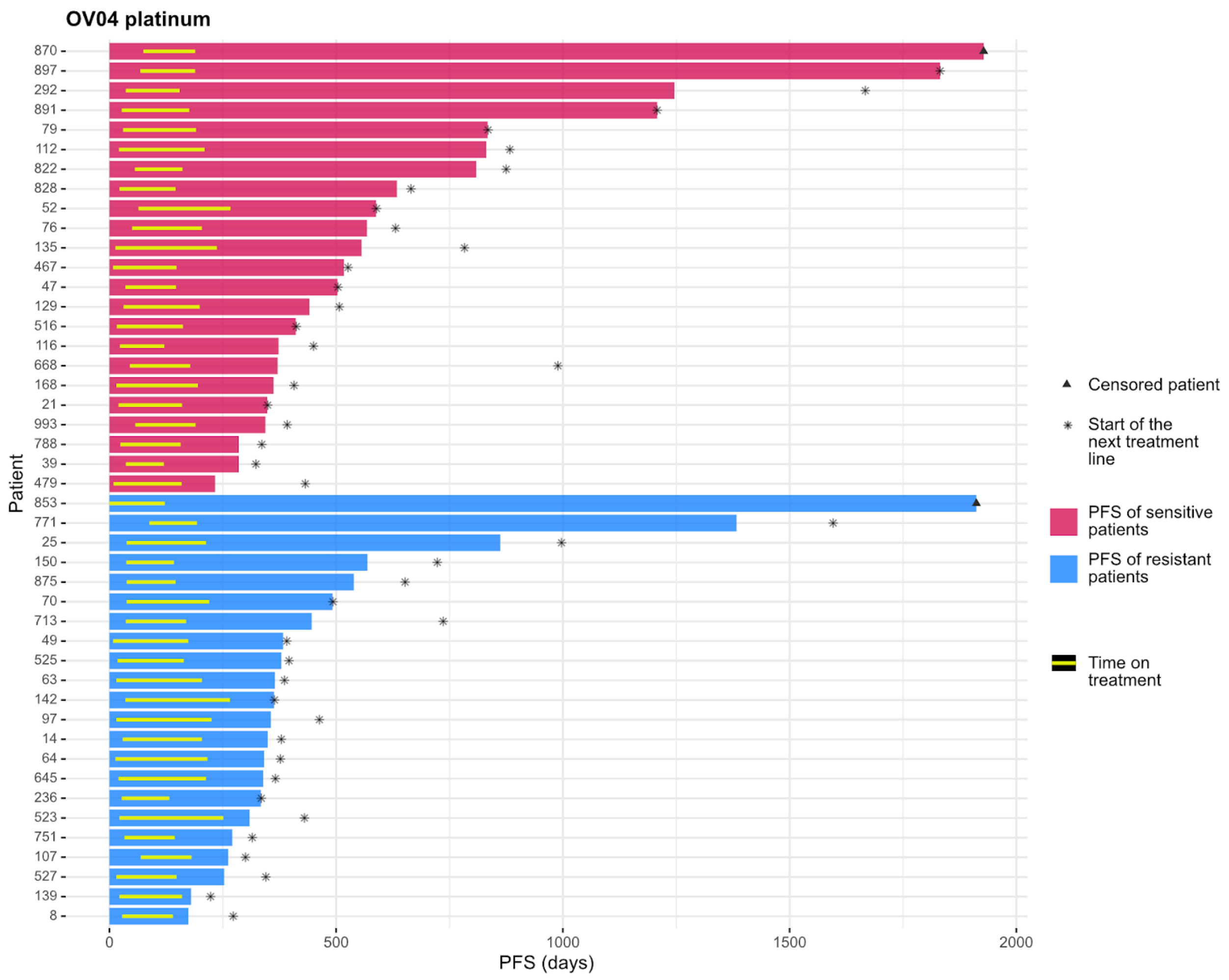

Supplementary Figure 2. Swimmer plot showing time on treatment, PFS and next treatment in OV04 patients included in the platinum cohort. Bars indicate progression-free survival. Asterisks indicate the start of the next treatment line, triangles indicate those patients that are censored, and yellow lines indicate time on treatment. No statistical difference in time on treatment was observed between resistant and sensitive patients (Wilcoxon rank sum test p-value = 0.44). Note: this visualisation does not take into account real-world covariates which may affect PFS and therefore visual differences between resistant and sensitive groups are limited.

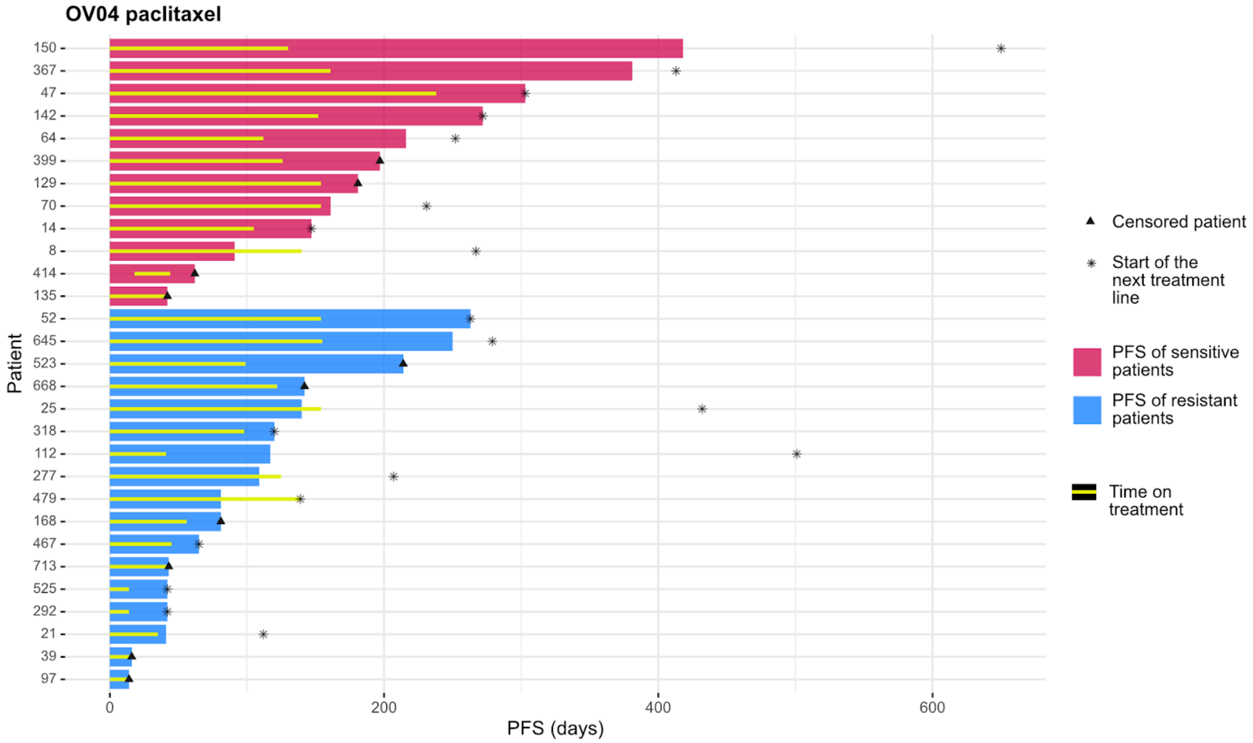

Supplementary Figure 3. Swimmer plot showing time on treatment, PFS and next treatment in OV04 patients included in the paclitaxel cohort. Bars indicate progression-free survival. Asterisks indicate the start of the next treatment line, triangles indicate those patients that are censored, and yellow lines indicate time on treatment. No statistical difference in time on treatment was observed between resistant and sensitive patients (Wilcoxon rank sum test p-value = 0.57). Note: this visualisation does not take into account real-world covariates which may affect PFS and therefore visual differences between resistant and sensitive groups are limited.

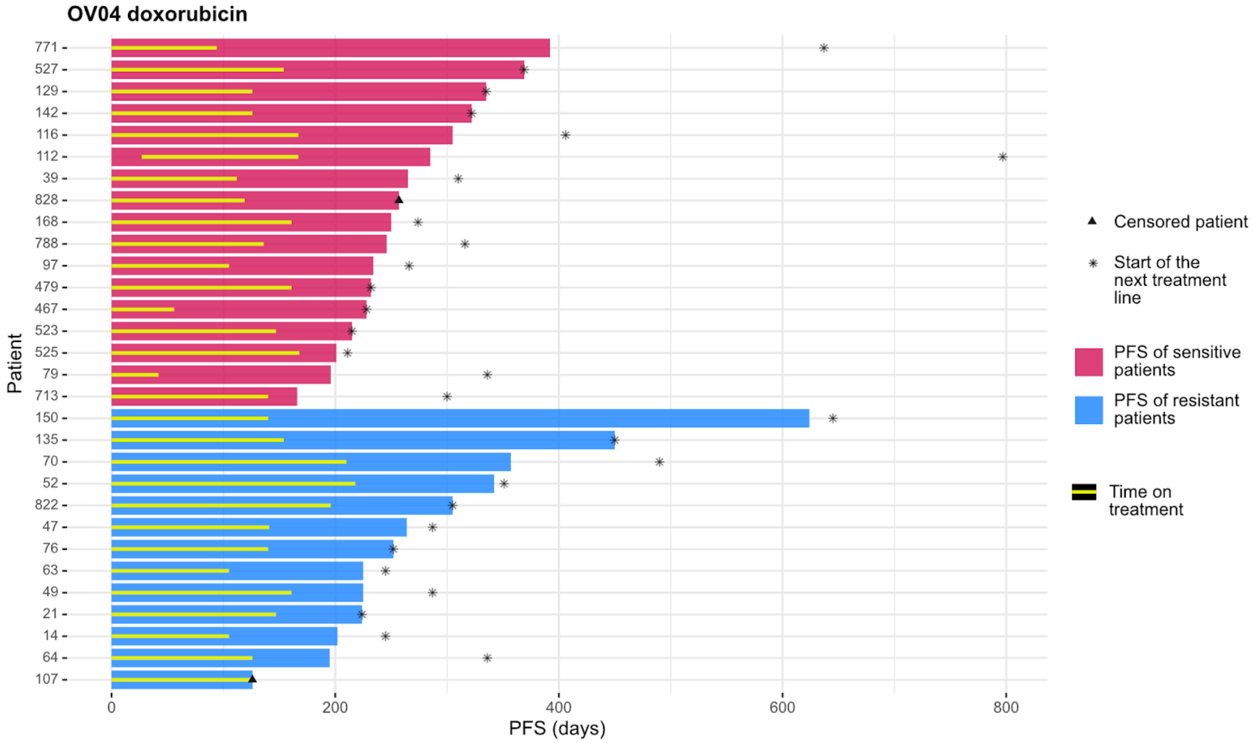

Supplementary Figure 4. Swimmer plot showing time on treatment, PFS and next treatment in OV04 patients included in the doxorubicin cohort. Bars indicate progression-free survival. Asterisks indicate the start of the next treatment line, triangles indicate those patients that are censored, and yellow lines indicate time on treatment. No statistical difference in time on treatment was observed between resistant and sensitive patients (Wilcoxon rank sum test p-value = 0.21). Note: this visualisation does not take into account real-world covariates which may affect PFS and therefore visual differences between resistant and sensitive groups are limited.

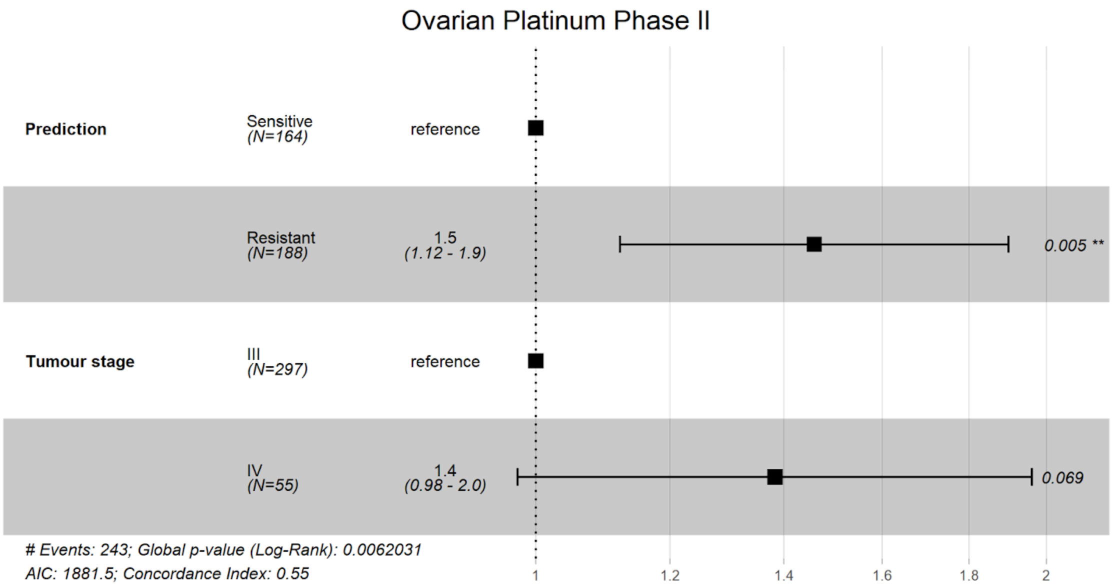

Supplementary Figure 5. Cox proportional hazards model for patients predicted as resistant to 1st-line platinum in TCGA-OV. Expanded from Figure 3a. Points and error bars represent HR and its 95% CI. Values on the left of the plot denote HR and its 95% CI, values on the right denote P-values. Significant P-values are identified by asterisks.

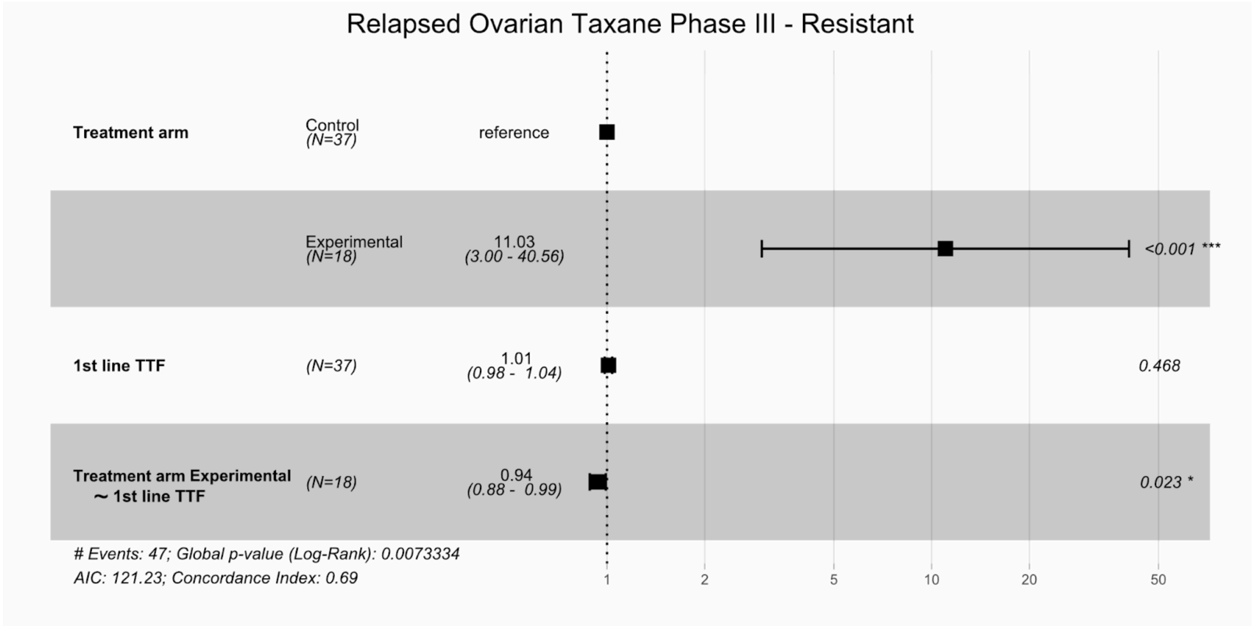

Supplementary Figure 6. Cox proportional hazards model for patients predicted as resistant to taxane-based treatment in TCGA-OV. Expanded from Figure 3b. Cox model was weighted by year of treatment. Points and error bars represent HR and its 95% CI. Values on the left of the plot denote HR and its 95% CI, values on the right denote P-values. Significant P-values are identified by asterisks.

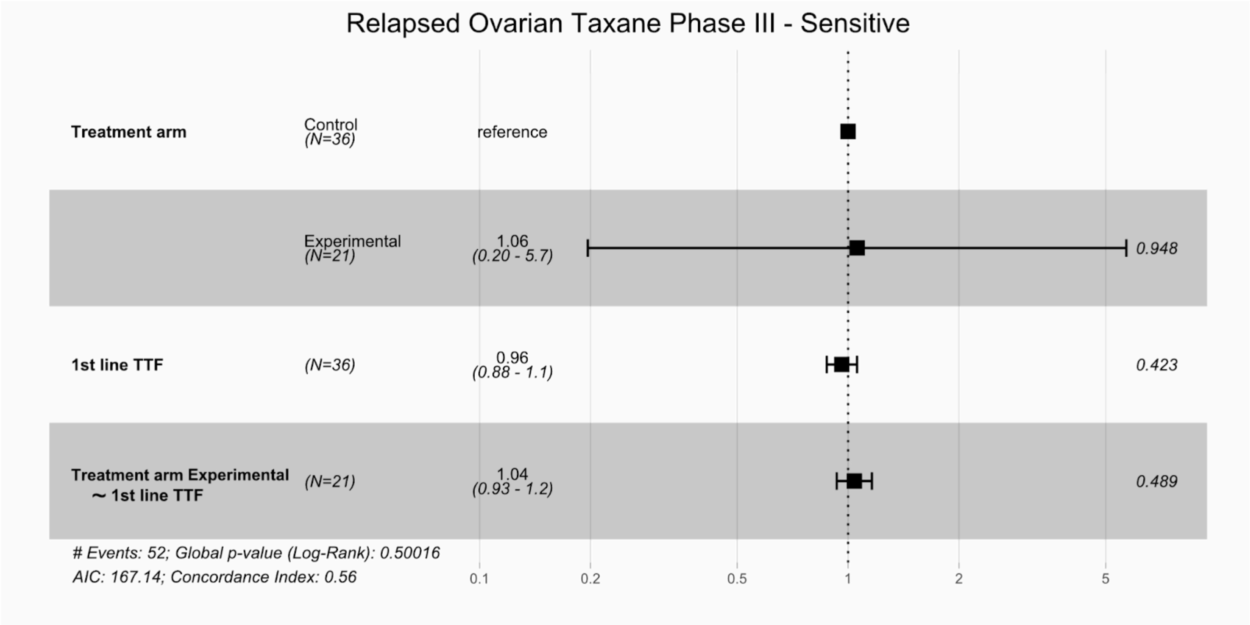

Supplementary Figure 7. Cox proportional hazards model for patients predicted as sensitive to taxane-based treatment in TCGA-OV. Expanded from Figure 3b. Cox model was weighted by year of treatment. Points and error bars represent HR and its 95% CI. Values on the left of the plot denote HR and its 95% CI, values on the right denote P-values. Significant P-values are identified by asterisks.

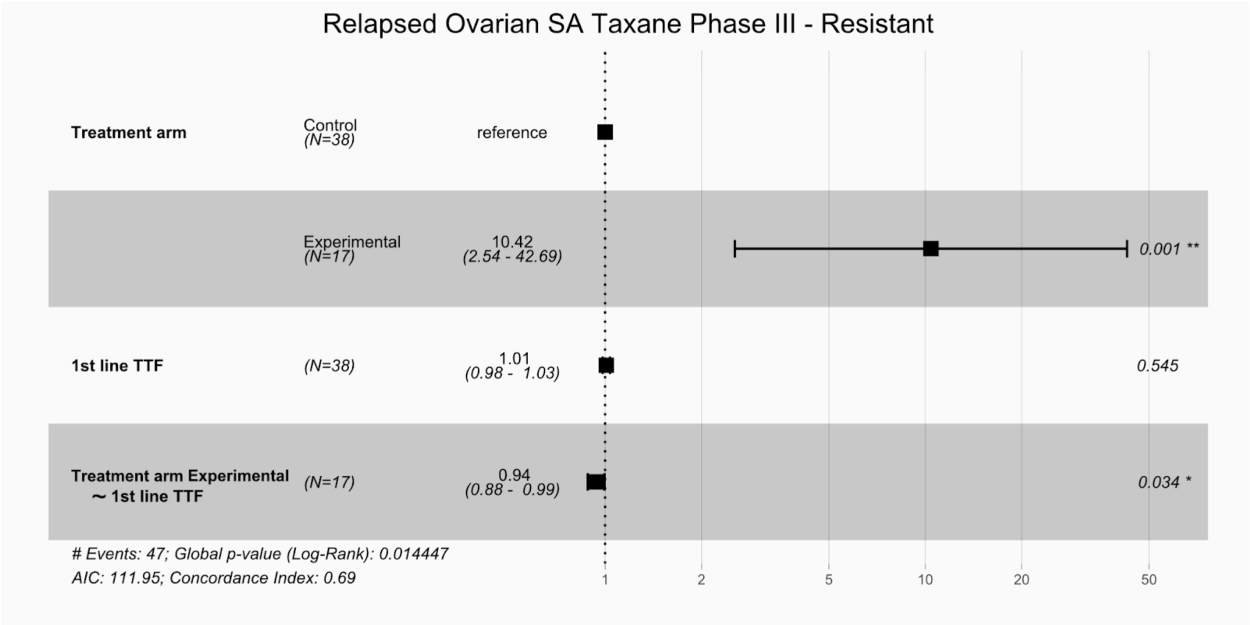

Supplementary Figure 8. Cox proportional hazards model for patients predicted as resistant to single-agent taxane in TCGA-OV. Cox model was weighted by year of treatment. Points and error bars represent HR and its 95% CI. Values on the left of the plot denote HR and its 95% CI, values on the right denote P-values. Significant P-values are identified by asterisks.

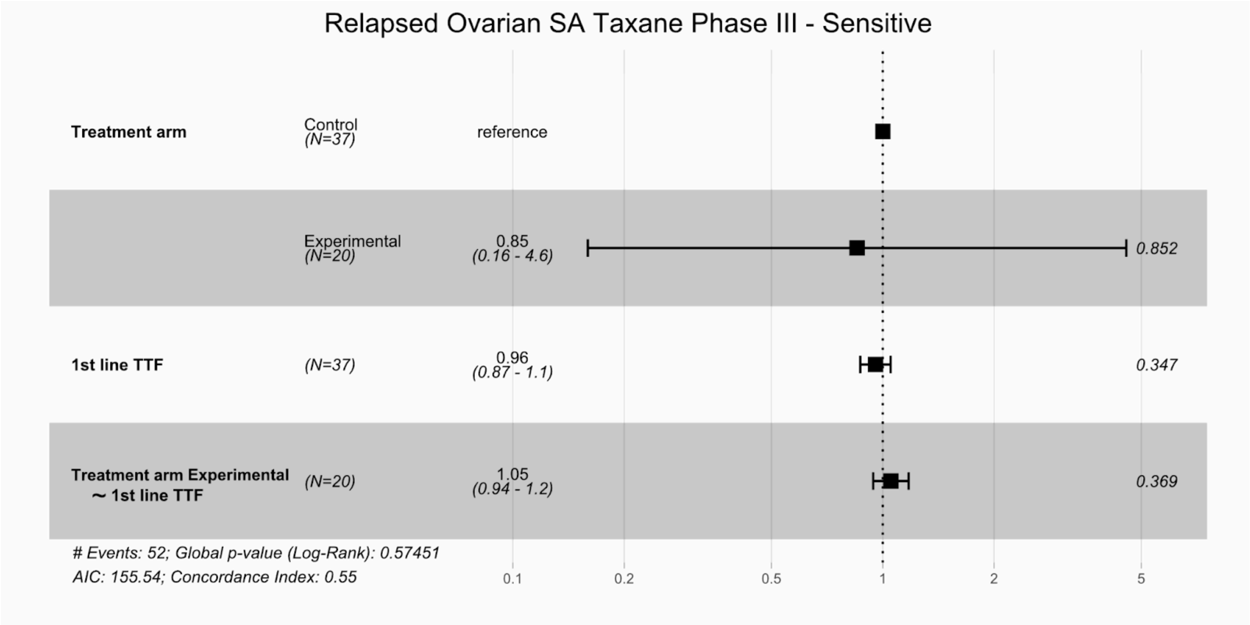

Supplementary Figure 9. Cox proportional hazards model for patients predicted as sensitive to single-agent taxane in TCGA-OV. Cox model was weighted by year of treatment. Points and error bars represent HR and its 95% CI. Values on the left of the plot denote HR and its 95% CI, values on the right denote P-values. Significant P-values are identified by asterisks.

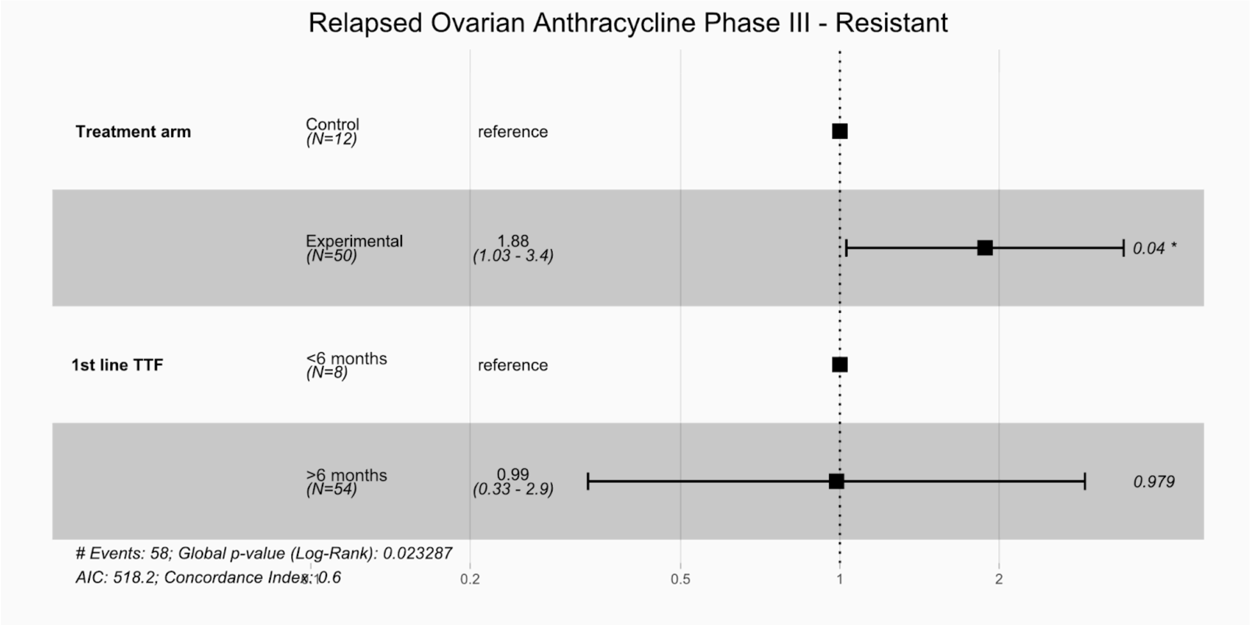

Supplementary Figure 10. Cox proportional hazards model for patients predicted as resistant to anthracycline-based treatment in TCGA-OV. Expanded from Figure 3c. Cox model was weighted by year of treatment. Points and error bars represent HR and its 95% CI. Values on the left of the plot denote HR and its 95% CI, values on the right denote P-values. Significant P-values are identified by asterisks.

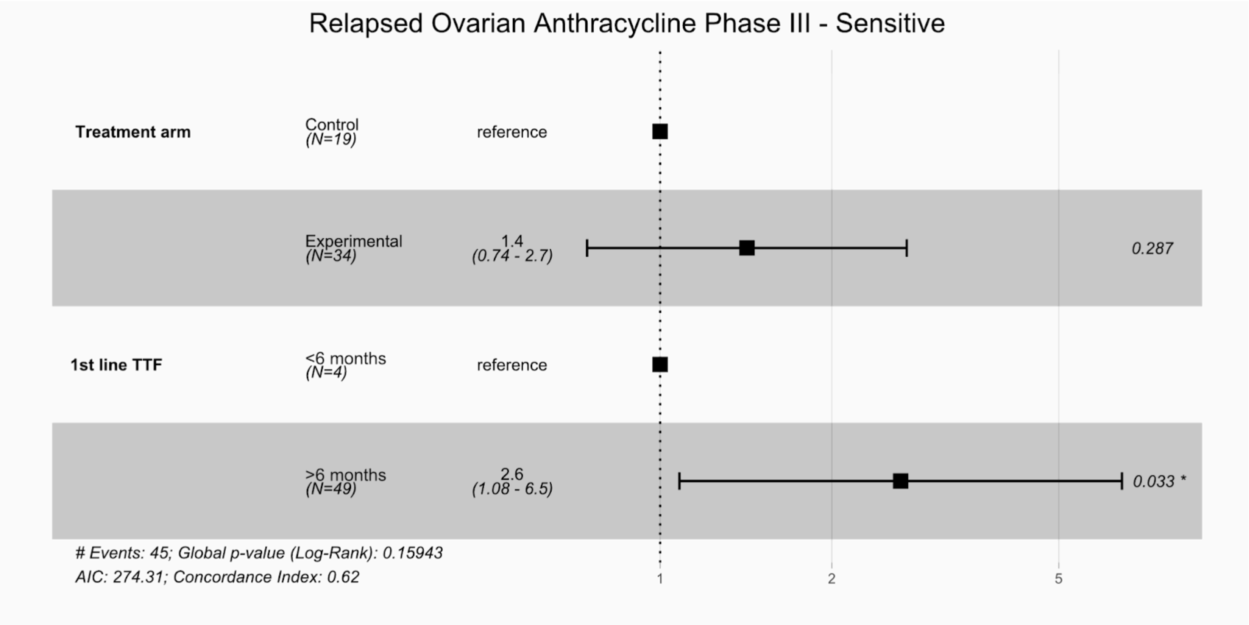

Supplementary Figure 11. Cox proportional hazards model for patients predicted as sensitive to anthracycline-based treatment in TCGA-OV. Expanded from Figure 3c. Cox model was weighted by year of treatment. Points and error bars represent HR and its 95% CI. Values on the left of the plot denote HR and its 95% CI, values on the right denote P-values. Significant P-values are identified by asterisks.

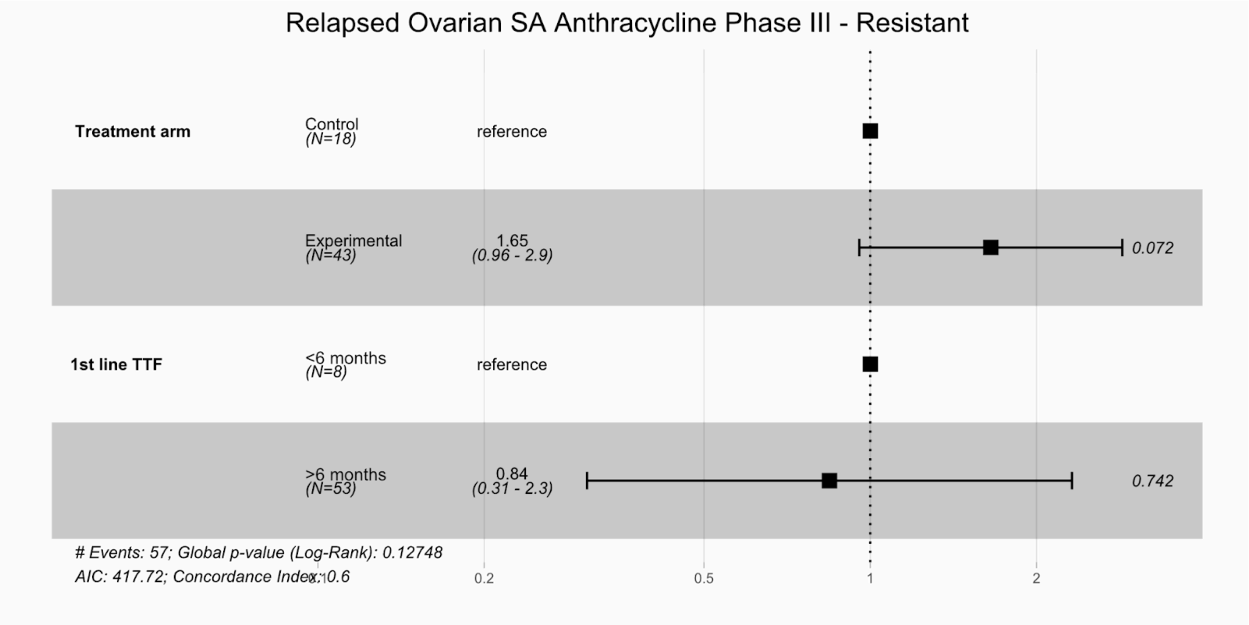

Supplementary Figure 12. Cox proportional hazards model for patients predicted as resistant to single-agent anthracycline in TCGA-OV. Cox model was weighted by year of treatment. Points and error bars represent HR and its 95% CI. Values on the left of the plot denote HR and its 95% CI, values on the right denote P-values. Significant P-values are identified by asterisks.

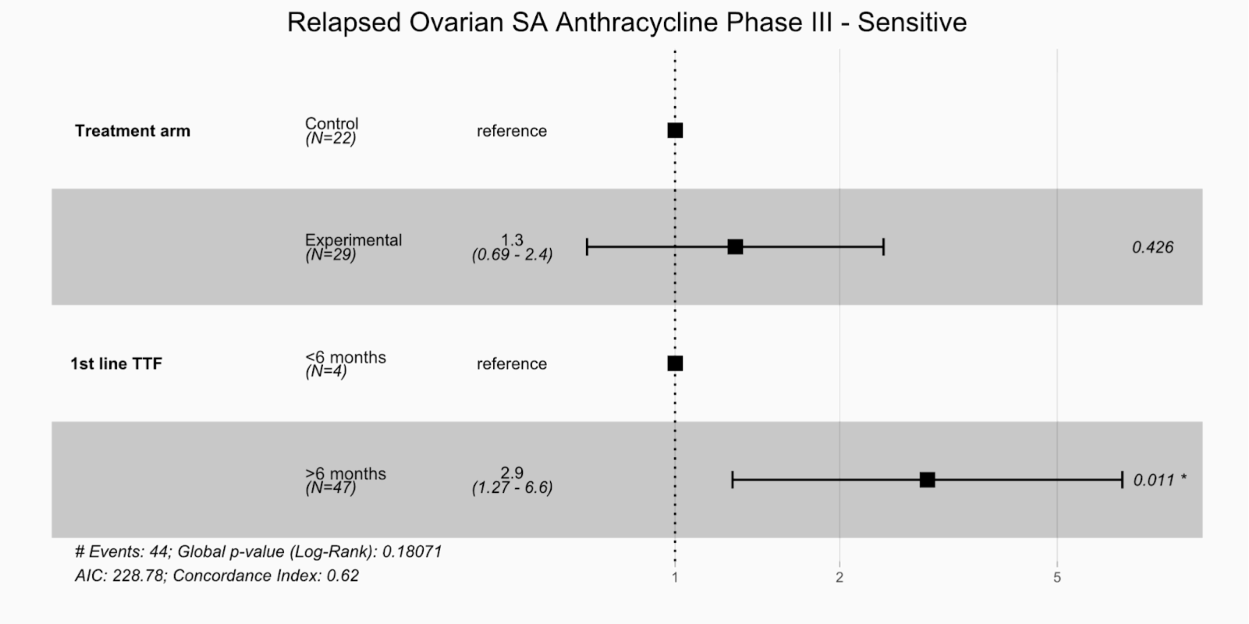

Supplementary Figure 13. Cox proportional hazards model for patients predicted as sensitive to single-agent anthracycline in TCGA-OV. Cox model was weighted by year of treatment. Points and error bars represent HR and its 95% CI. Values on the left of the plot denote HR and its 95% CI, values on the right denote P-values. Significant P-values are identified by asterisks.

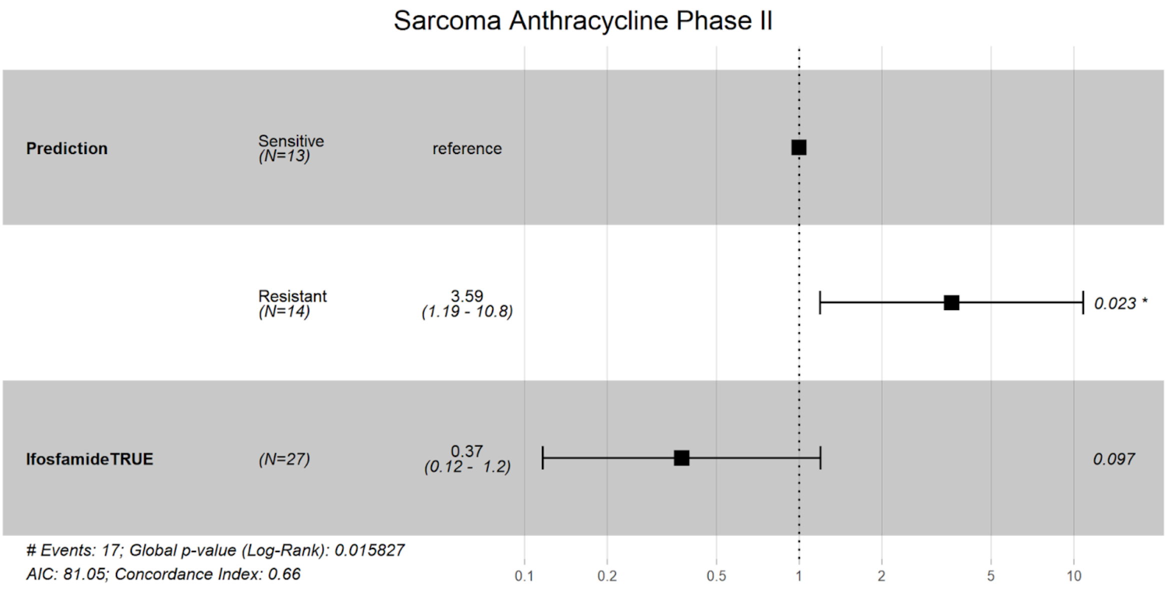

Supplementary Figure 14. Cox proportional hazards model for patients predicted as resistant to anthracyclines in TCGA-SARC. Expanded from Figure 3d. Points and error bars represent HR and its 95% CI. Values on the left of the plot denote HR and its 95% CI, values on the right denote P-values. Significant P-values are identified by asterisks.

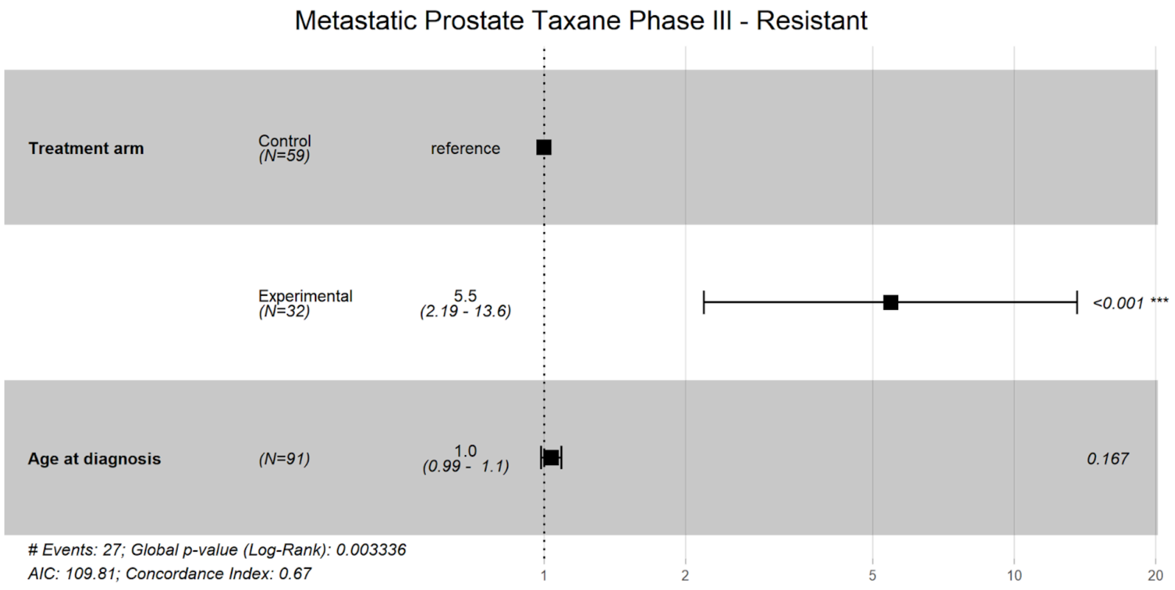

Supplementary Figure 15. Cox proportional hazards model for patients predicted as resistant to taxane-based treatment in HMF-Prostate. Expanded from Figure 4a. Cox model was weighted by year of biopsy. Points and error bars represent HR and its 95% CI. Values on the left of the plot denote HR and its 95% CI, values on the right denote P-values. Significant P-values are identified by asterisks.

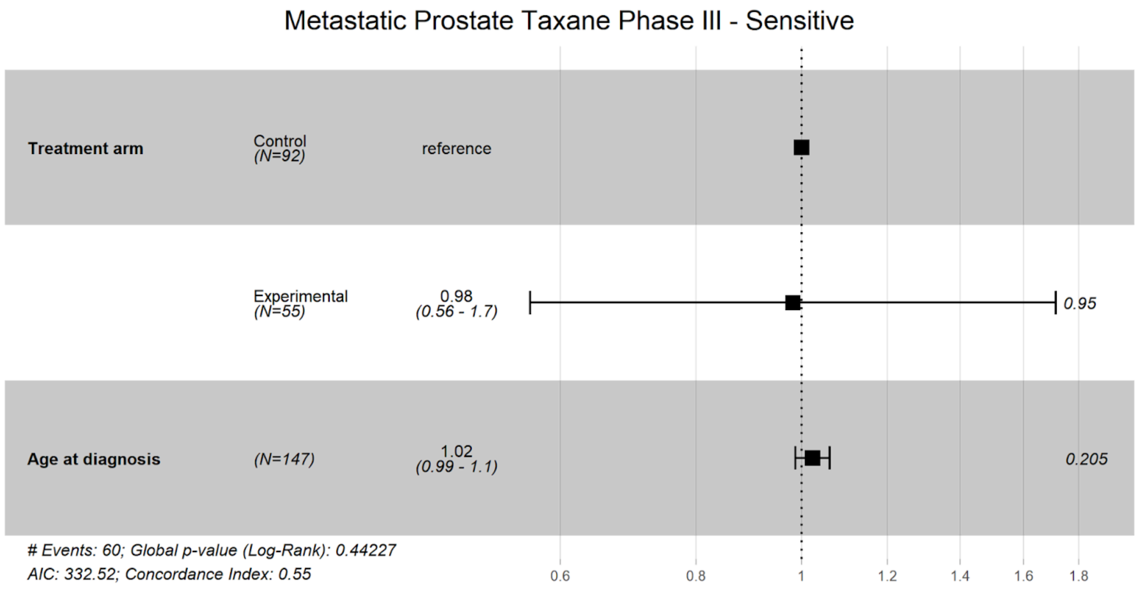

Supplementary Figure 16. Cox proportional hazards model for patients predicted as sensitive to taxane-based treatment in HMF-Prostate. Expanded from Figure 4a. Cox model was weighted by year of biopsy. Points and error bars represent HR and its 95% CI. Values on the left of the plot denote HR and its 95% CI, values on the right denote P-values. Significant P-values are identified by asterisks.

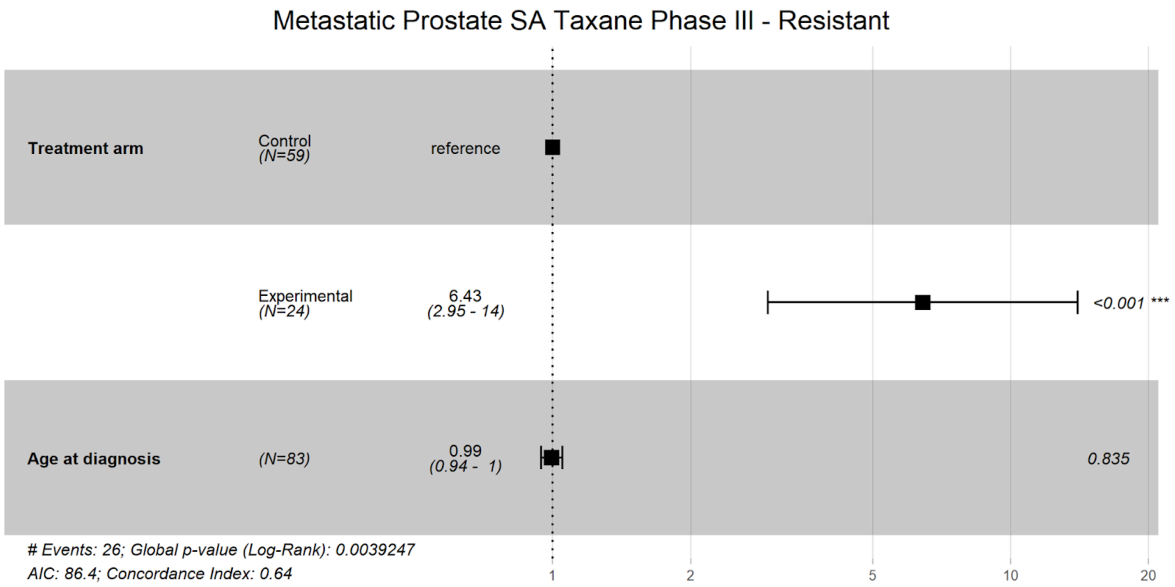

Supplementary Figure 17. Cox proportional hazards model for patients predicted as resistant to single-agent taxane treatment in HMF-Prostate. Cox model was weighted by year of biopsy. Points and error bars represent HR and its 95% CI. Values on the left of the plot denote HR and its 95% CI, values on the right denote P-values. Significant P-values are identified by asterisks.

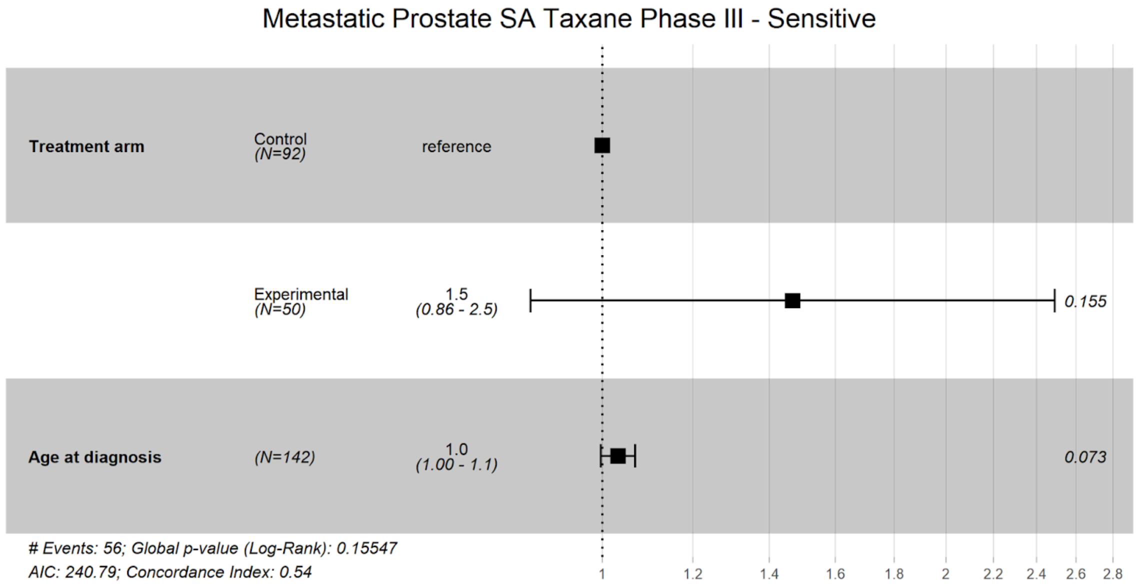

Supplementary Figure 18. Cox proportional hazards model for patients predicted as sensitive to single-agent taxane treatment in HMF-Prostate. Cox model was weighted by year of biopsy. Points and error bars represent HR and its 95% CI. Values on the left of the plot denote HR and its 95% CI, values on the right denote P-values. Significant P-values are identified by asterisks.

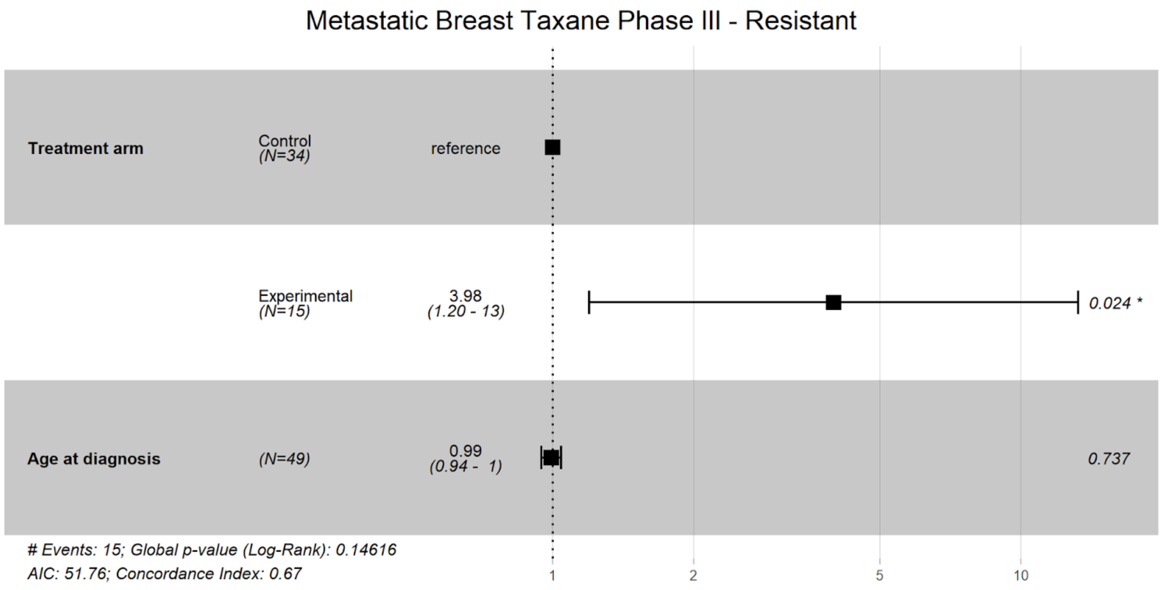

Supplementary Figure 19. Cox proportional hazards model for patients predicted as resistant to taxane-based treatment in HMF-Breast. Expanded from Figure 4b. Cox model was weighted by year of biopsy. Points and error bars represent HR and its 95% CI. Values on the left of the plot denote HR and its 95% CI, values on the right denote P-values. Significant P-values are identified by asterisks.

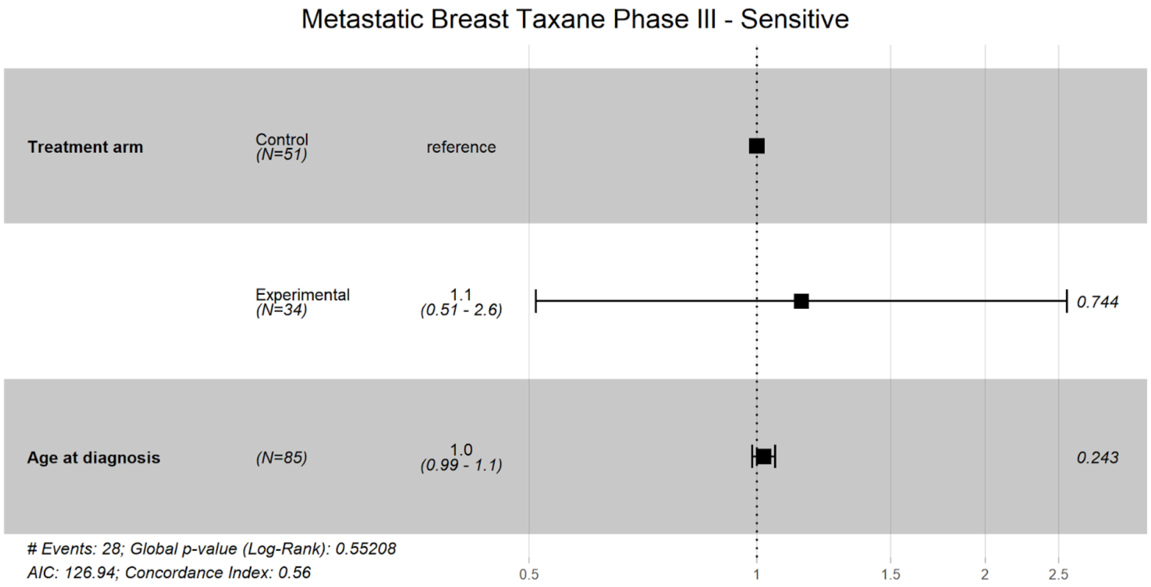

Supplementary Figure 20. Cox proportional hazards model for patients predicted as sensitive to taxane-based treatment in HMF-Breast. Expanded from Figure 4b. Cox model was weighted by year of biopsy. Points and error bars represent HR and its 95% CI. Values on the left of the plot denote HR and its 95% CI, values on the right denote P-values. Significant P-values are identified by asterisks.

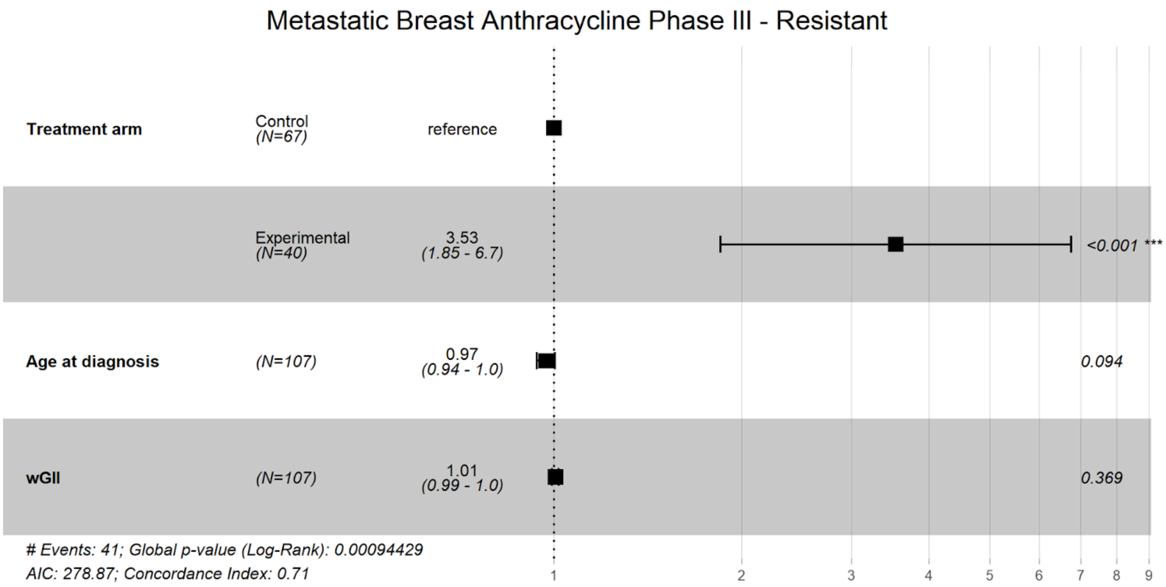

Supplementary Figure 21. Cox proportional hazards model for patients predicted as resistant to anthracycline-based treatment in HMF-Breast. Expanded from Figure 4c. Cox model was weighted by year of biopsy. Points and error bars represent HR and its 95% CI. Values on the left of the plot denote HR and its 95% CI, values on the right denote P-values. Significant P-values are identified by asterisks.

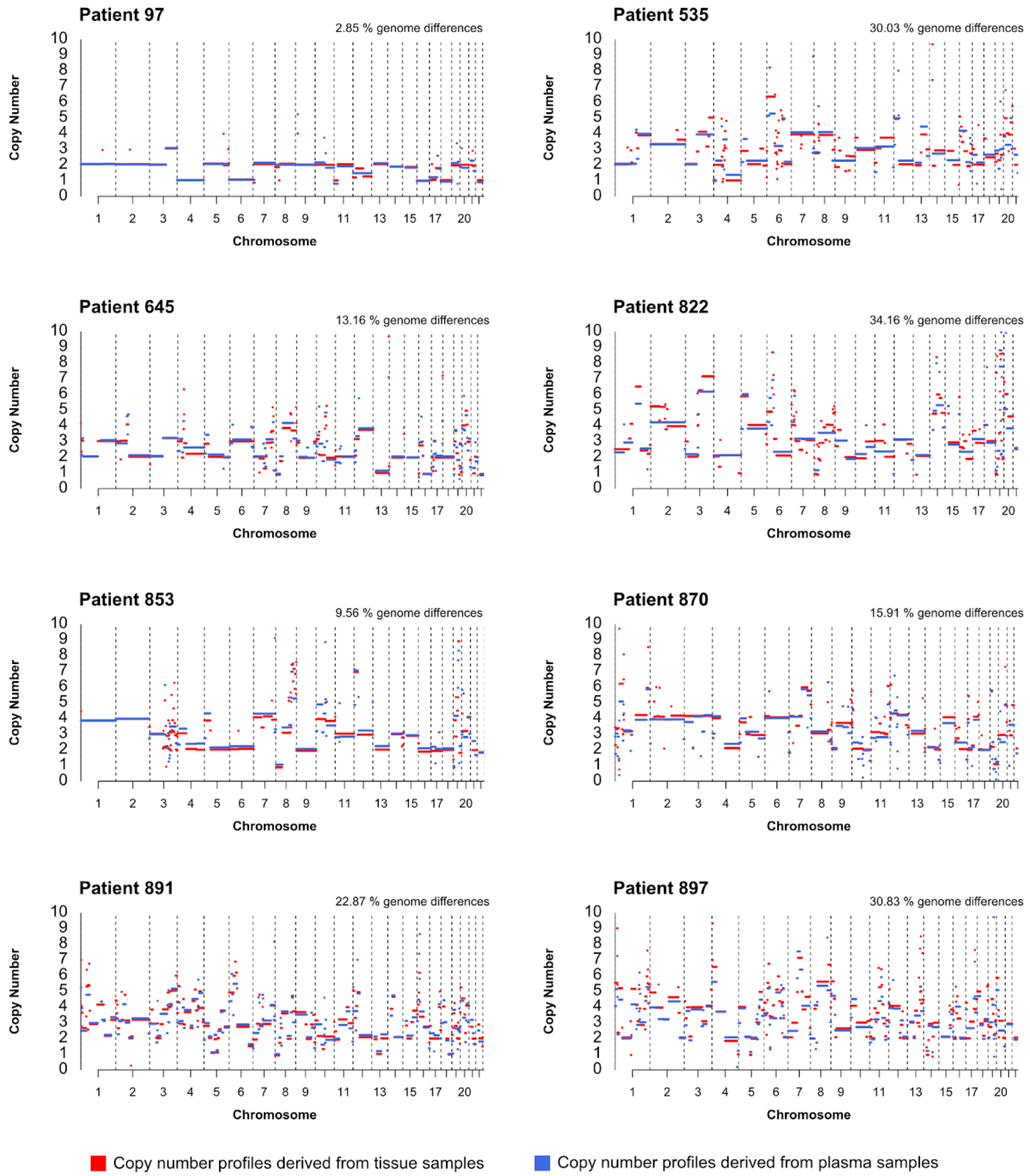

Supplementary Figure 22. Comparison of copy number profiles of tumour tissues derived from sWGS and TSO500. The quantification of the extent of genome differences between pairs was calculated using the *CNpare* tool in R.

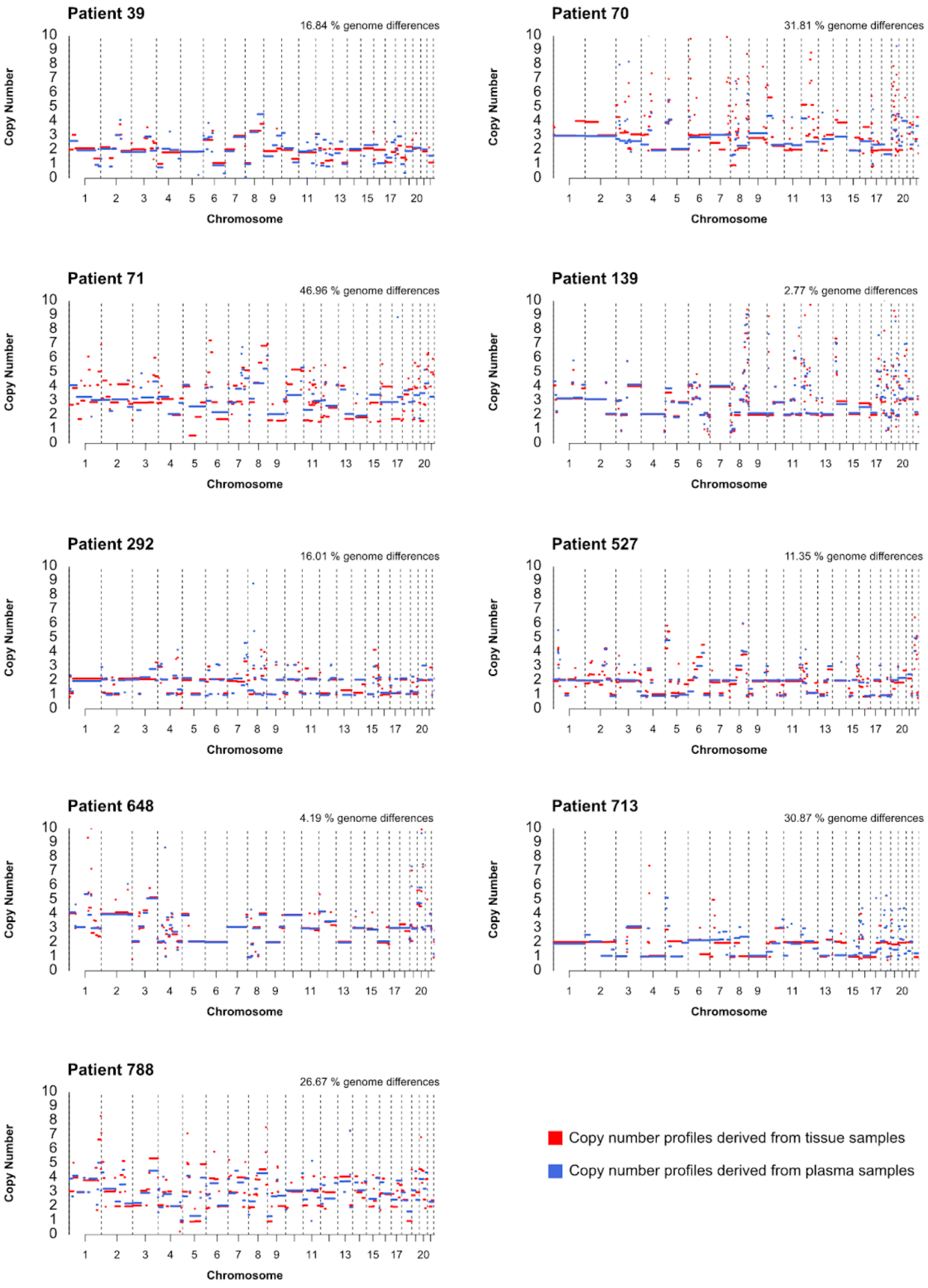

Supplementary Figure 23. Comparison of copy number profiles derived from tissue or liquid biopsy. The quantification of the extent of genome differences between pairs was calculated using the *CNpare* tool in R.

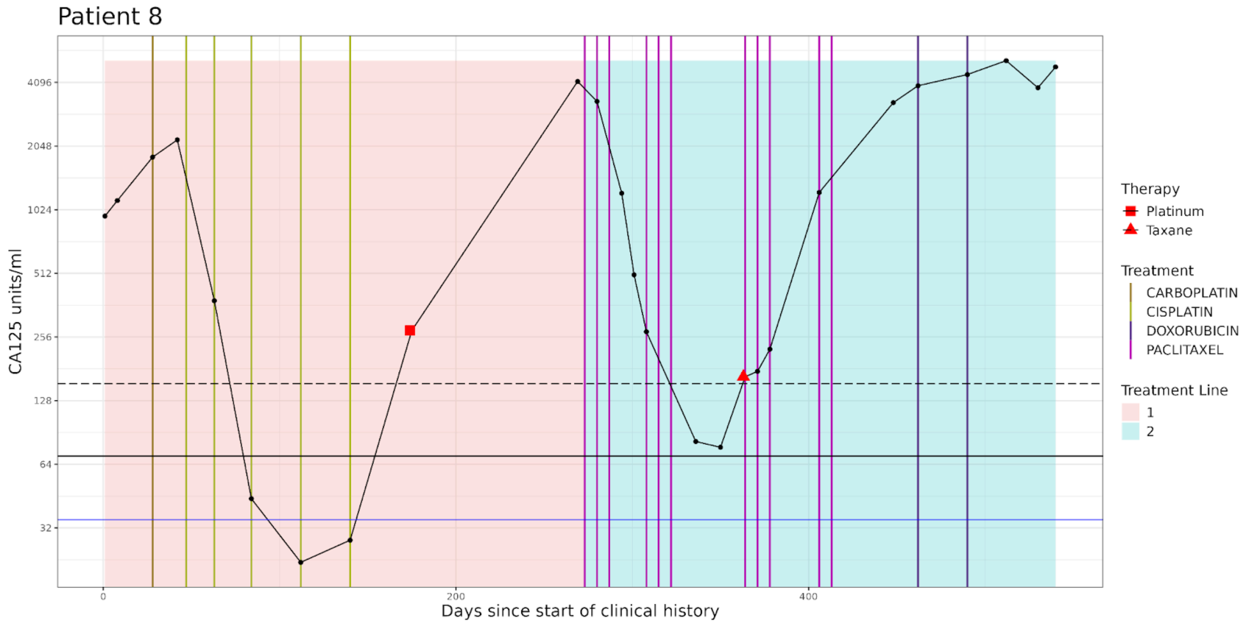

Supplementary Figure 24. Clinical history plot for OV04 patient 8. Blood serum CA125 levels are shown over time as a line graph, and points on the line marked in red show the calculated date of progression. The horizontal lines in black show the CA125 thresholds required for progression, specific to each chemotherapy of interest. Vertical lines denote individual treatment administrations, and background panels indicate which treatment line the administration belongs to. The horizontal bar at 35 units/ml denotes the threshold between ‘normal’ and ‘abnormal’ CA125 readings. In cases where multiple treatments are given on the same day, the treatment date is shifted slightly to show all treatments.

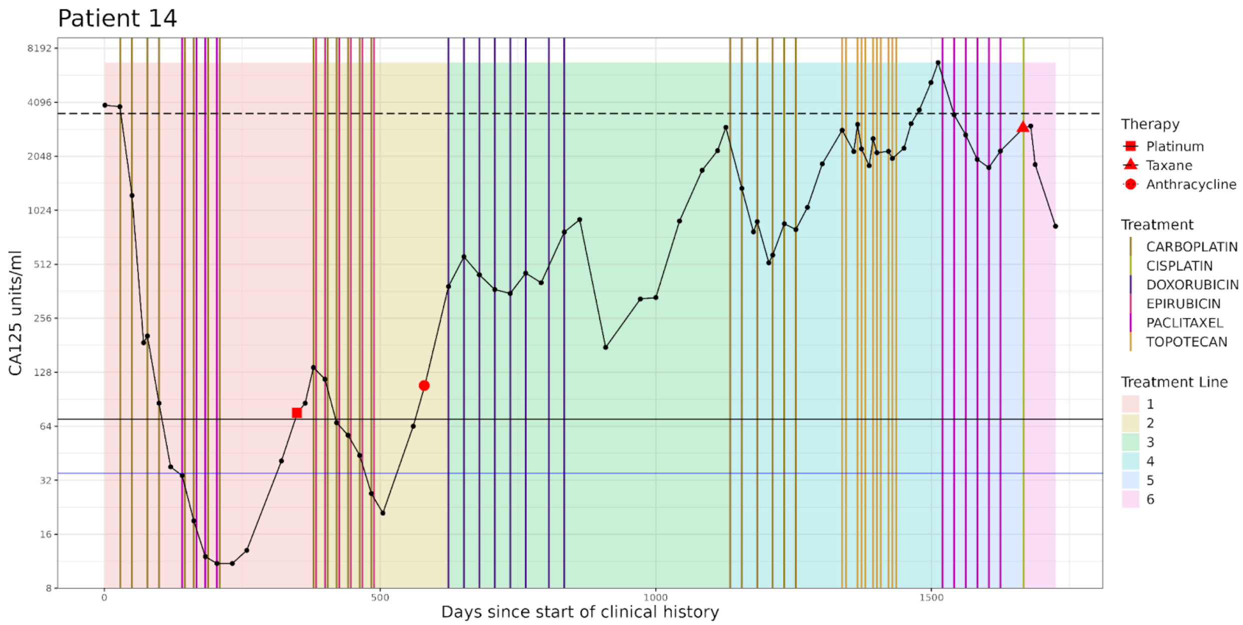

Supplementary Figure 25. Clinical history plot for OV04 patient 14. Blood serum CA125 levels are shown over time as a line graph, and points on the line marked in red show the calculated date of progression. The horizontal lines in black show the CA125 thresholds required for progression, specific to each chemotherapy of interest. Vertical lines denote individual treatment administrations, and background panels indicate which treatment line the administration belongs to. The horizontal bar at 35 units/ml denotes the threshold between ‘normal’ and ‘abnormal’ CA125 readings. In cases where multiple treatments are given on the same day, the treatment date is shifted slightly to show all treatments.

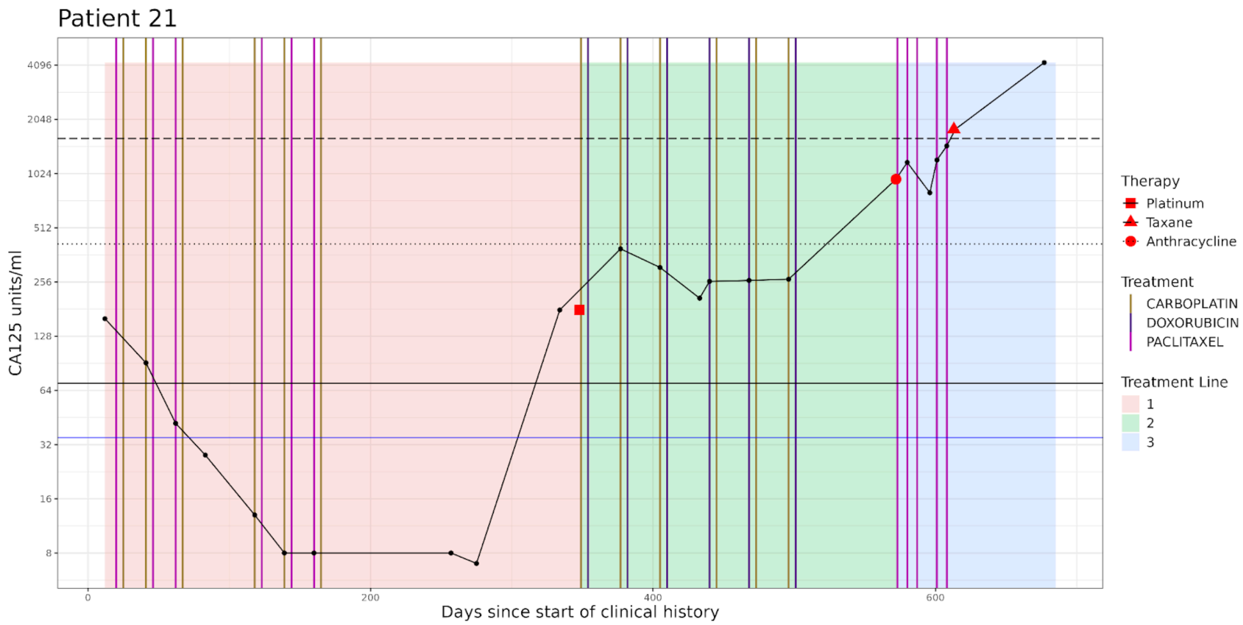

Supplementary Figure 26. Clinical history plot for OV04 patient 21. Blood serum CA125 levels are shown over time as a line graph, and points on the line marked in red show the calculated date of progression. The horizontal lines in black show the CA125 thresholds required for progression, specific to each chemotherapy of interest. Vertical lines denote individual treatment administrations, and background panels indicate which treatment line the administration belongs to. The horizontal bar at 35 units/ml denotes the threshold between ‘normal’ and ‘abnormal’ CA125 readings. In cases where multiple treatments are given on the same day, the treatment date is shifted slightly to show all treatments.

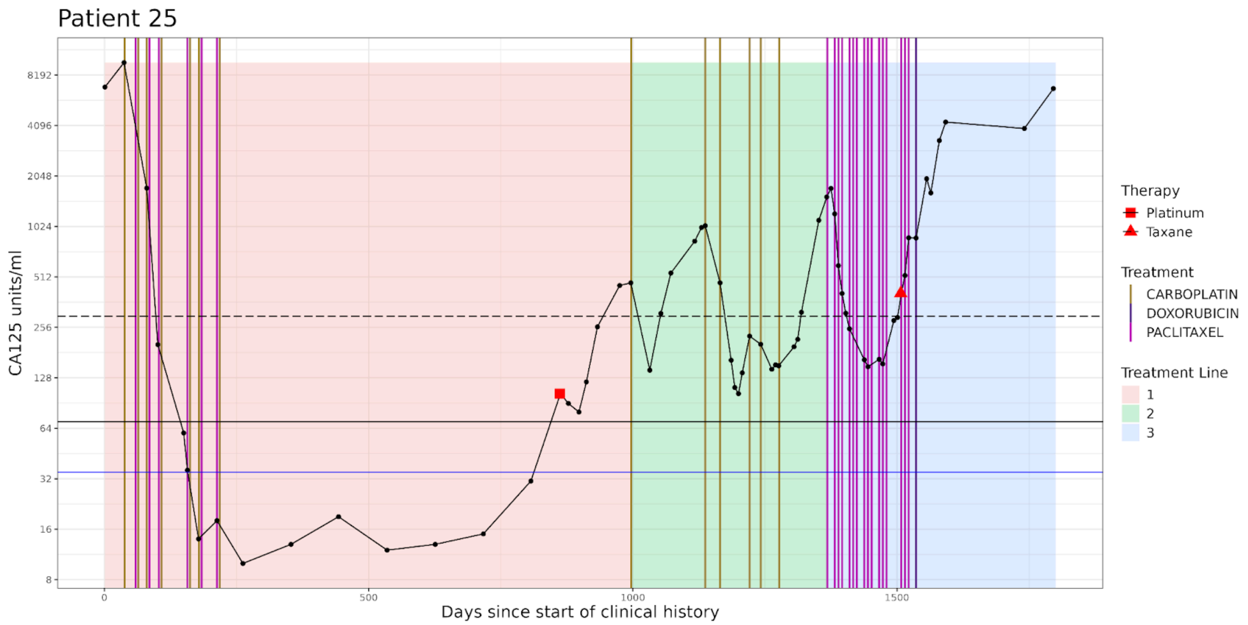

Supplementary Figure 27. Clinical history plot for OV04 patient 25. Blood serum CA125 levels are shown over time as a line graph, and points on the line marked in red show the calculated date of progression. The horizontal lines in black show the CA125 thresholds required for progression, specific to each chemotherapy of interest. Vertical lines denote individual treatment administrations, and background panels indicate which treatment line the administration belongs to. The horizontal bar at 35 units/ml denotes the threshold between ‘normal’ and ‘abnormal’ CA125 readings. In cases where multiple treatments are given on the same day, the treatment date is shifted slightly to show all treatments.

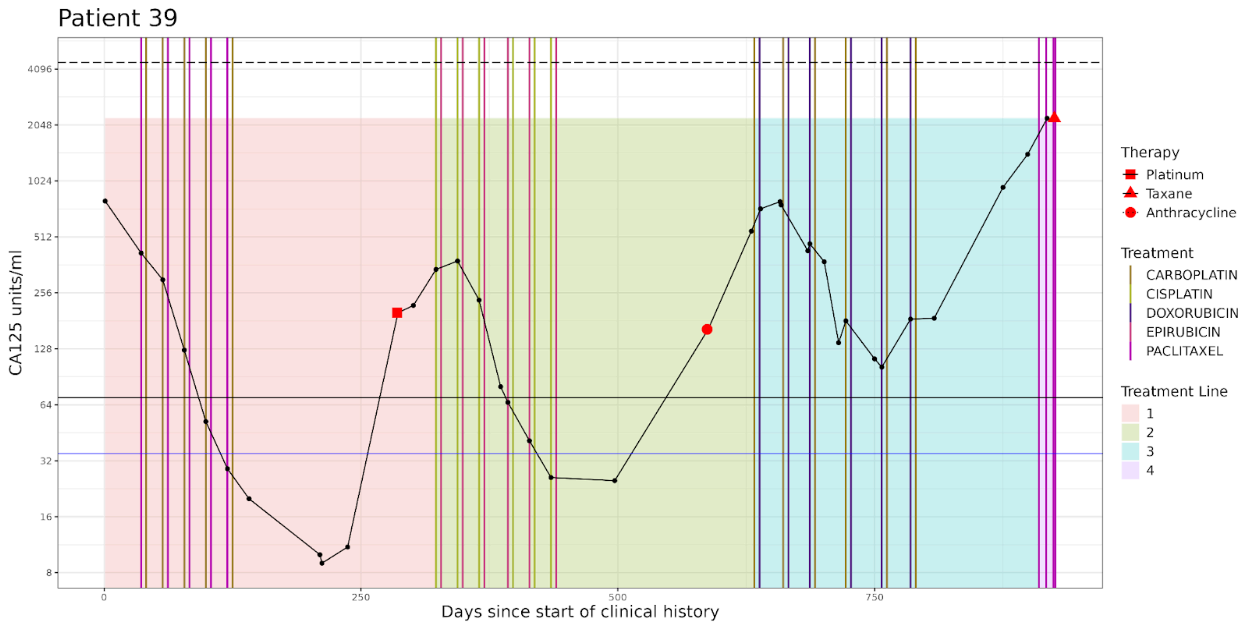

Supplementary Figure 28. Clinical history plot for OV04 patient 39. Blood serum CA125 levels are shown over time as a line graph, and points on the line marked in red show the calculated date of progression. The horizontal lines in black show the CA125 thresholds required for progression, specific to each chemotherapy of interest. Vertical lines denote individual treatment administrations, and background panels indicate which treatment line the administration belongs to. The horizontal bar at 35 units/ml denotes the threshold between 'normal' and 'abnormal' CA125 readings. In cases where multiple treatments are given on the same day, the treatment date is shifted slightly to show all treatments.

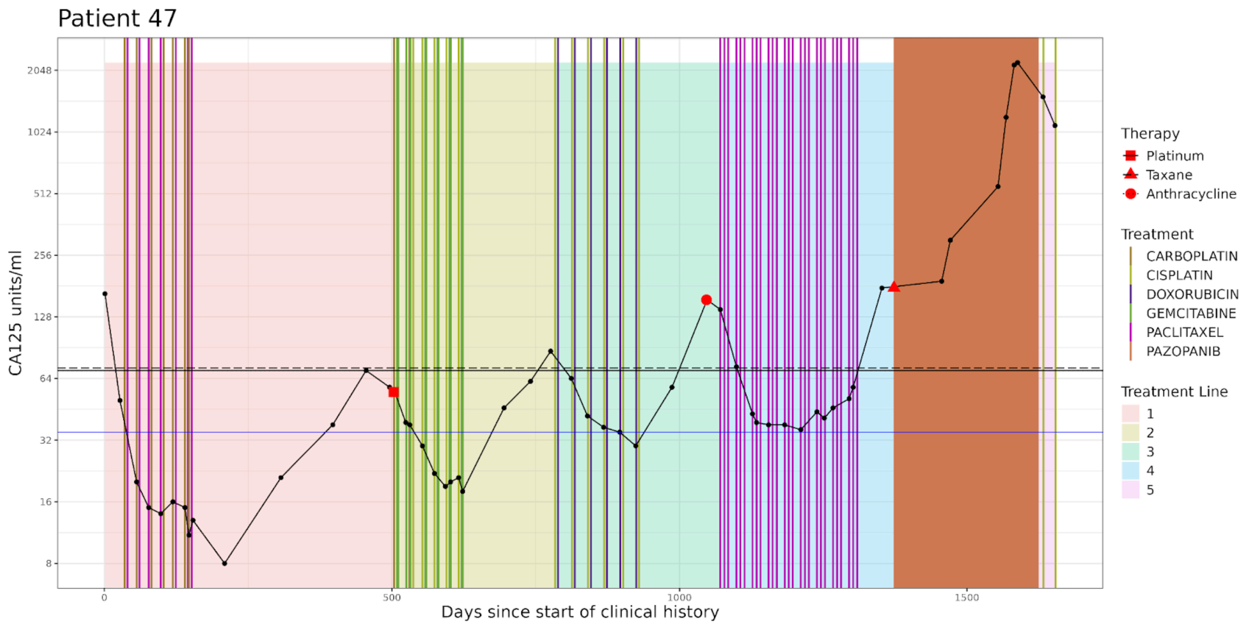

Supplementary Figure 29. Clinical history plot for OV04 patient 47. Blood serum CA125 levels are shown over time as a line graph, and points on the line marked in red show the calculated date of progression. The horizontal lines in black show the CA125 thresholds required for progression, specific to each chemotherapy of interest. Vertical lines denote individual treatment administrations, and background panels indicate which treatment line the administration belongs to. The horizontal bar at 35 units/ml denotes the threshold between 'normal' and 'abnormal' CA125 readings. In cases where multiple treatments are given on the same day, the treatment date is shifted slightly to show all treatments.

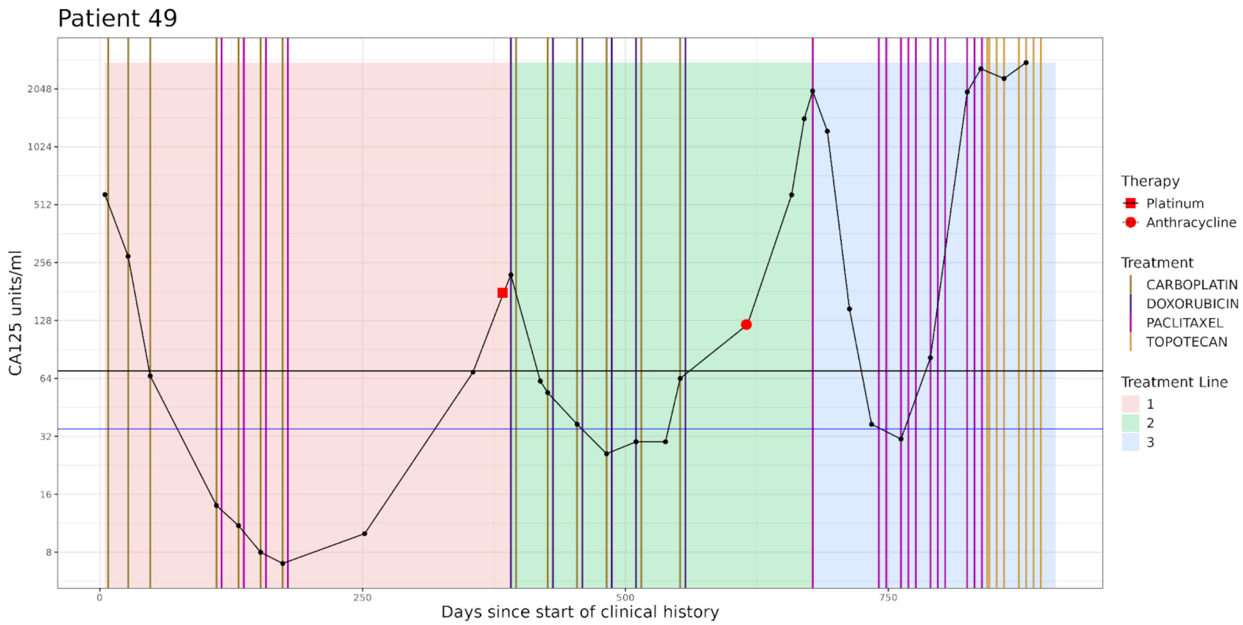

Supplementary Figure 30. Clinical history plot for OV04 patient 49. Blood serum CA125 levels are shown over time as a line graph, and points on the line marked in red show the calculated date of progression. The horizontal lines in black show the CA125 thresholds required for progression, specific to each chemotherapy of interest. Vertical lines denote individual treatment administrations, and background panels indicate which treatment line the administration belongs to. The horizontal bar at 35 units/ml denotes the threshold between 'normal' and 'abnormal' CA125 readings. In cases where multiple treatments are given on the same day, the treatment date is shifted slightly to show all treatments.

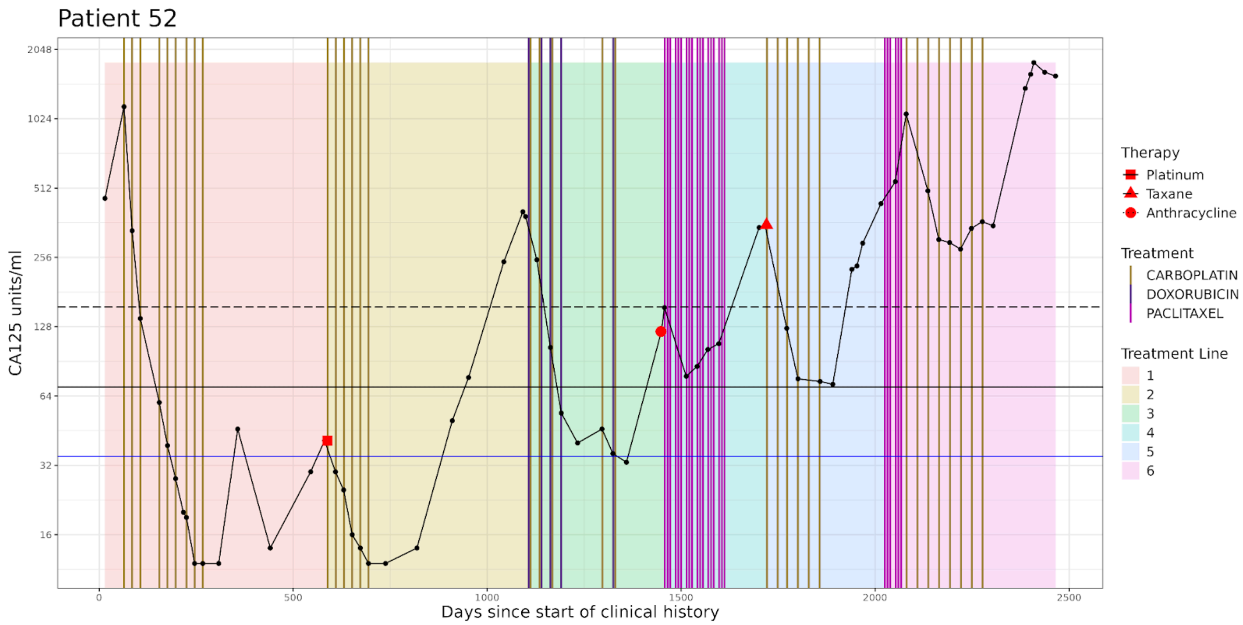

Supplementary Figure 31. Clinical history plot for OV04 patient 52. Blood serum CA125 levels are shown over time as a line graph, and points on the line marked in red show the calculated date of progression. The horizontal lines in black show the CA125 thresholds required for progression, specific to each chemotherapy of interest. Vertical lines denote individual treatment administrations, and background panels indicate which treatment line the administration belongs to. The horizontal bar at 35 units/ml denotes the threshold between ‘normal’ and ‘abnormal’ CA125 readings. In cases where multiple treatments are given on the same day, the treatment date is shifted slightly to show all treatments.

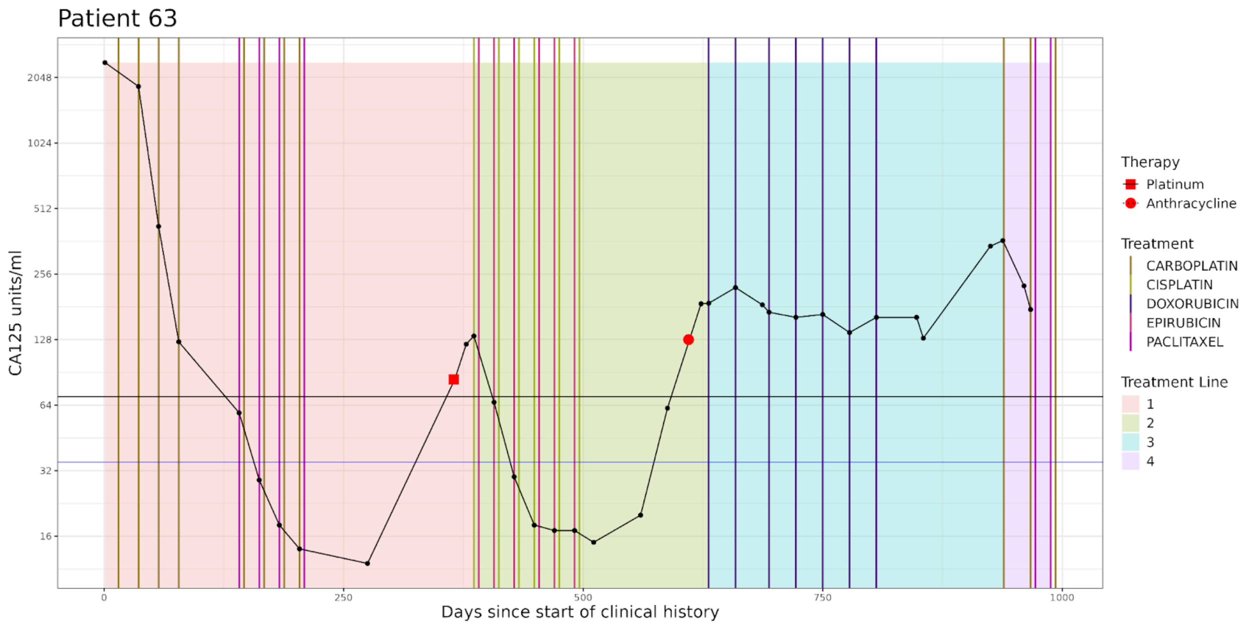

Supplementary Figure 32. Clinical history plot for OV04 patient 63. Blood serum CA125 levels are shown over time as a line graph, and points on the line marked in red show the calculated date of progression. The horizontal lines in black show the CA125 thresholds required for progression, specific to each chemotherapy of interest. Vertical lines denote individual treatment administrations, and background panels indicate which treatment line the administration belongs to. The horizontal bar at 35 units/ml denotes the threshold between ‘normal’ and ‘abnormal’ CA125 readings. In cases where multiple treatments are given on the same day, the treatment date is shifted slightly to show all treatments.

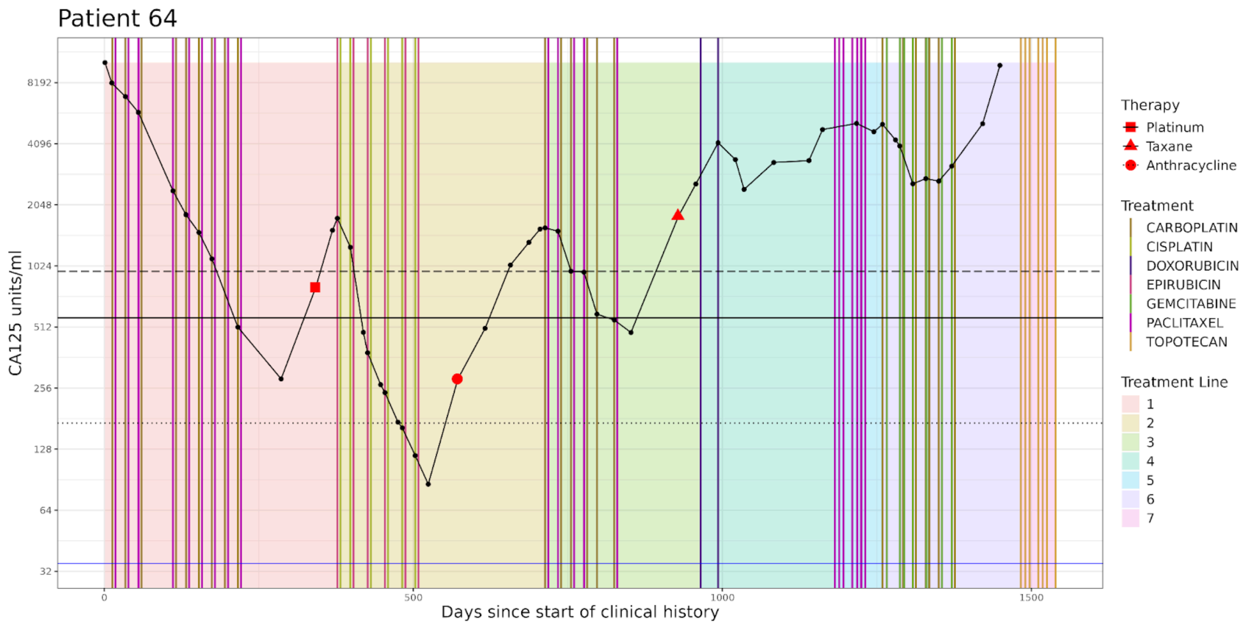

Supplementary Figure 33. Clinical history plot for OV04 patient 64. Blood serum CA125 levels are shown over time as a line graph, and points on the line marked in red show the calculated date of progression. The horizontal lines in black show the CA125 thresholds required for progression, specific to each chemotherapy of interest. Vertical lines denote individual treatment administrations, and background panels indicate which treatment line the administration belongs to. The horizontal bar at 35 units/ml denotes the threshold between 'normal' and 'abnormal' CA125 readings. In cases where multiple treatments are given on the same day, the treatment date is shifted slightly to show all treatments.

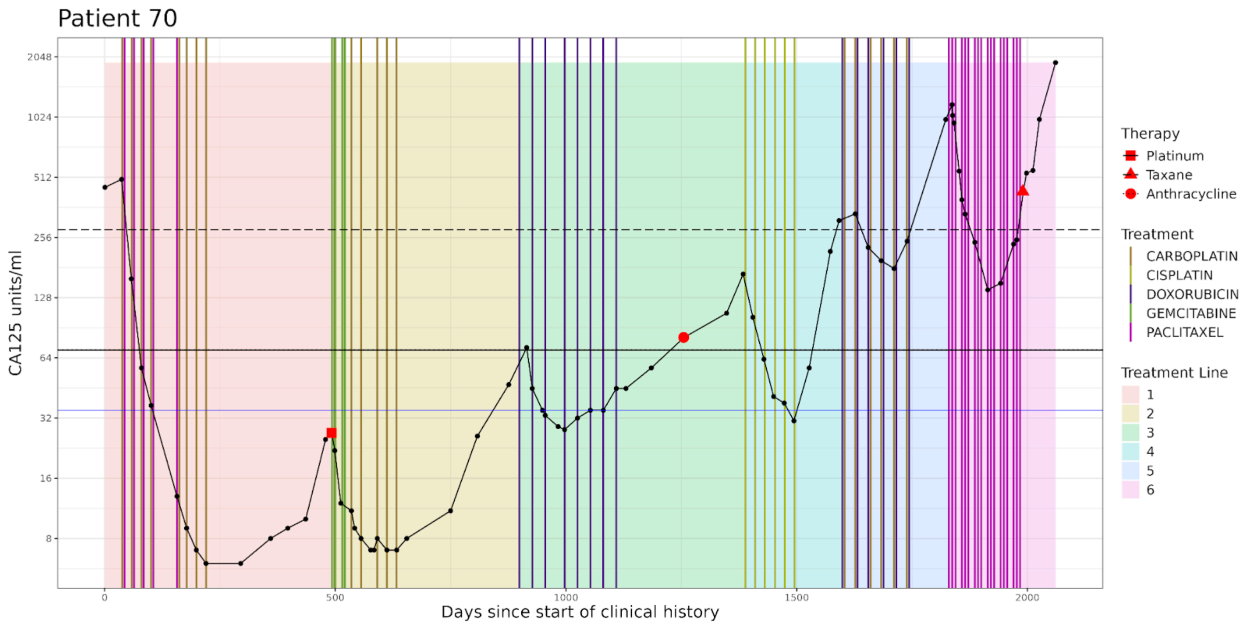

Supplementary Figure 34. Clinical history plot for OV04 patient 70. Blood serum CA125 levels are shown over time as a line graph, and points on the line marked in red show the calculated date of progression. The horizontal lines in black show the CA125 thresholds required for progression, specific to each chemotherapy of interest. Vertical lines denote individual treatment administrations, and background panels indicate which treatment line the administration belongs to. The horizontal bar at 35 units/ml denotes the threshold between 'normal' and 'abnormal' CA125 readings. In cases where multiple treatments are given on the same day, the treatment date is shifted slightly to show all treatments.

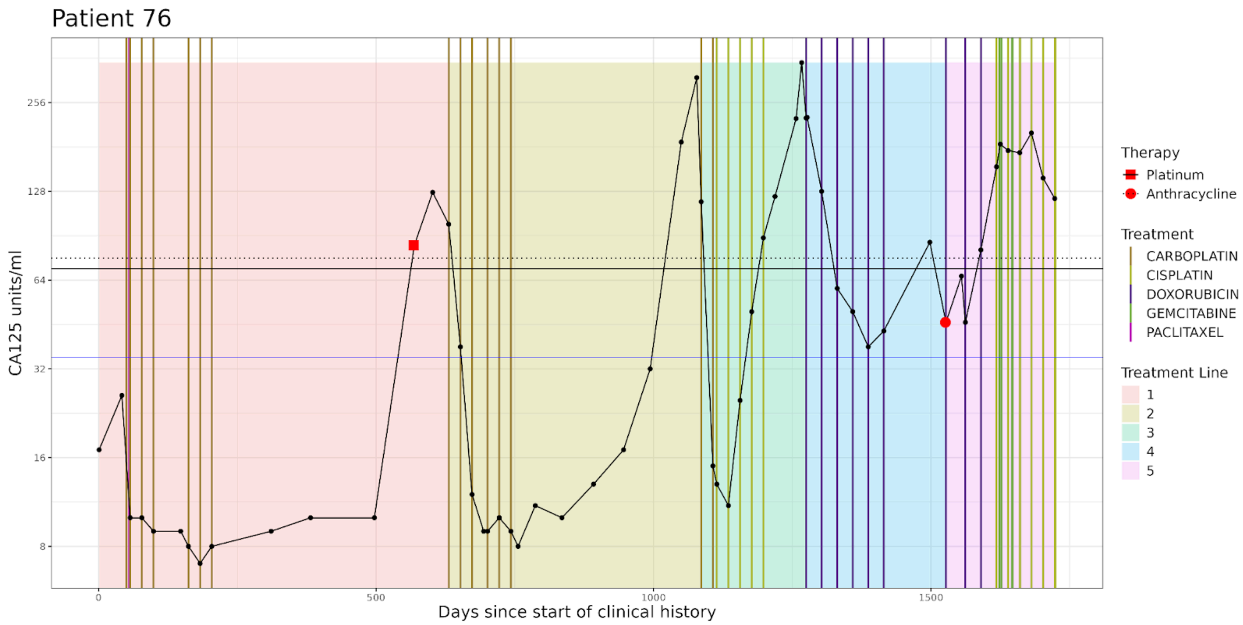

Supplementary Figure 35. Clinical history plot for OV04 patient 76. Blood serum CA125 levels are shown over time as a line graph, and points on the line marked in red show the calculated date of progression. The horizontal lines in black show the CA125 thresholds required for progression, specific to each chemotherapy of interest. Vertical lines denote individual treatment administrations, and background panels indicate which treatment line the administration belongs to. The horizontal bar at 35 units/ml denotes the threshold between ‘normal’ and ‘abnormal’ CA125 readings. In cases where multiple treatments are given on the same day, the treatment date is shifted slightly to show all treatments.

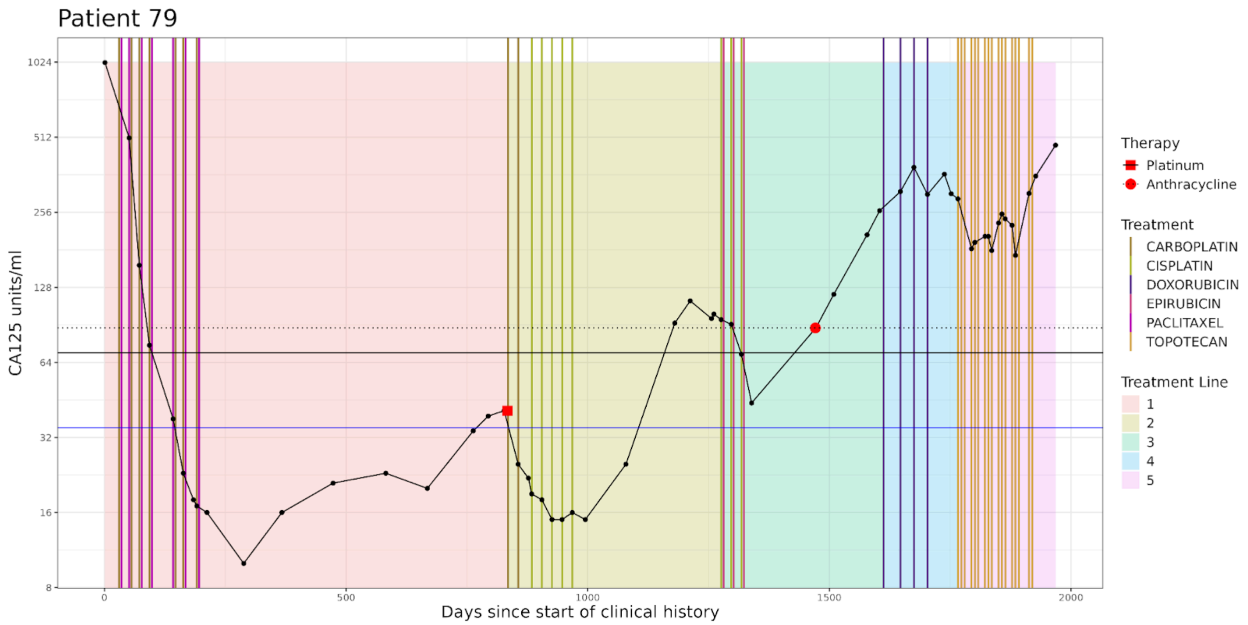

Supplementary Figure 36. Clinical history plot for OV04 patient 79. Blood serum CA125 levels are shown over time as a line graph, and points on the line marked in red show the calculated date of progression. The horizontal lines in black show the CA125 thresholds required for progression, specific to each chemotherapy of interest. Vertical lines denote individual treatment administrations, and background panels indicate which treatment line the administration belongs to. The horizontal bar at 35 units/ml denotes the threshold between ‘normal’ and ‘abnormal’ CA125 readings. In cases where multiple treatments are given on the same day, the treatment date is shifted slightly to show all treatments.

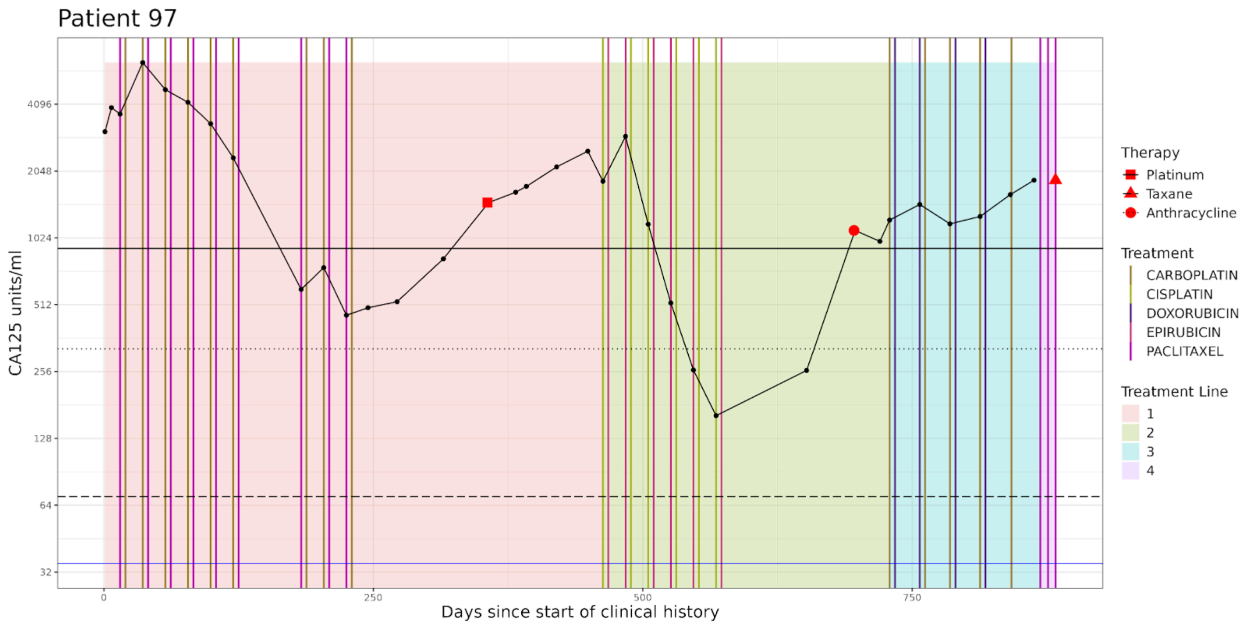

Supplementary Figure 37. Clinical history plot for OV04 patient 97. Blood serum CA125 levels are shown over time as a line graph, and points on the line marked in red show the calculated date of progression. The horizontal lines in black show the CA125 thresholds required for progression, specific to each chemotherapy of interest. Vertical lines denote individual treatment administrations, and background panels indicate which treatment line the administration belongs to. The horizontal bar at 35 units/ml denotes the threshold between 'normal' and 'abnormal' CA125 readings. In cases where multiple treatments are given on the same day, the treatment date is shifted slightly to show all treatments.

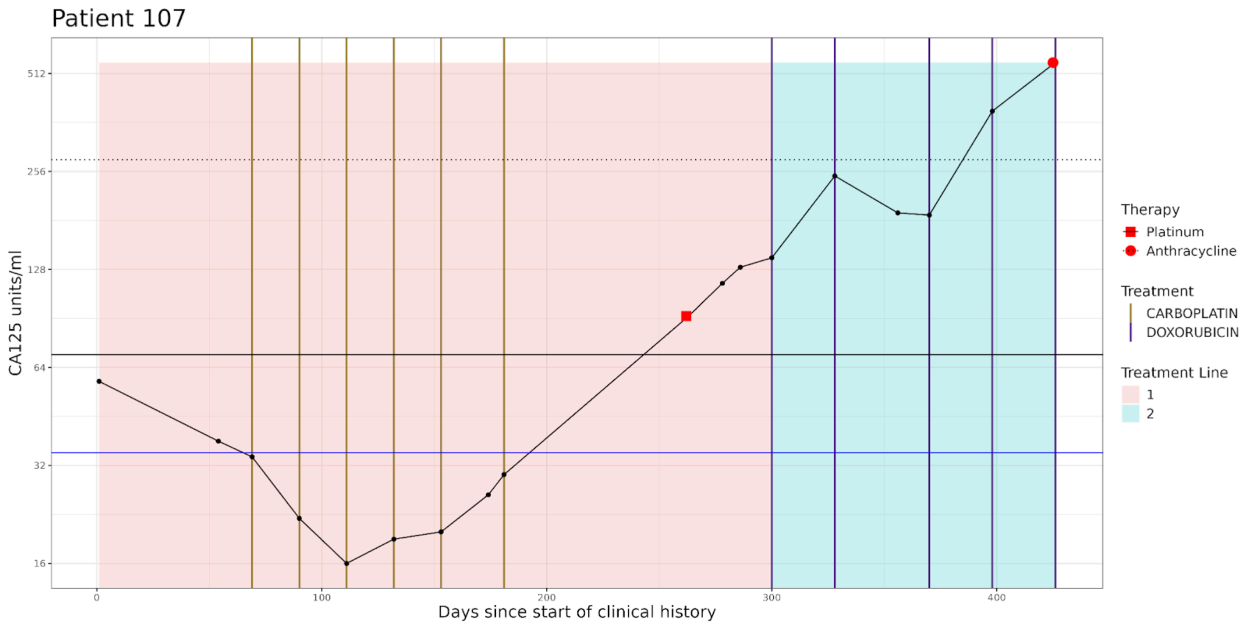

Supplementary Figure 38. Clinical history plot for OV04 patient 107. Blood serum CA125 levels are shown over time as a line graph, and points on the line marked in red show the calculated date of progression. The horizontal lines in black show the CA125 thresholds required for progression, specific to each chemotherapy of interest. Vertical lines denote individual treatment administrations, and background panels indicate which treatment line the administration belongs to. The horizontal bar at 35 units/ml denotes the threshold between ‘normal’ and ‘abnormal’ CA125 readings. In cases where multiple treatments are given on the same day, the treatment date is shifted slightly to show all treatments.

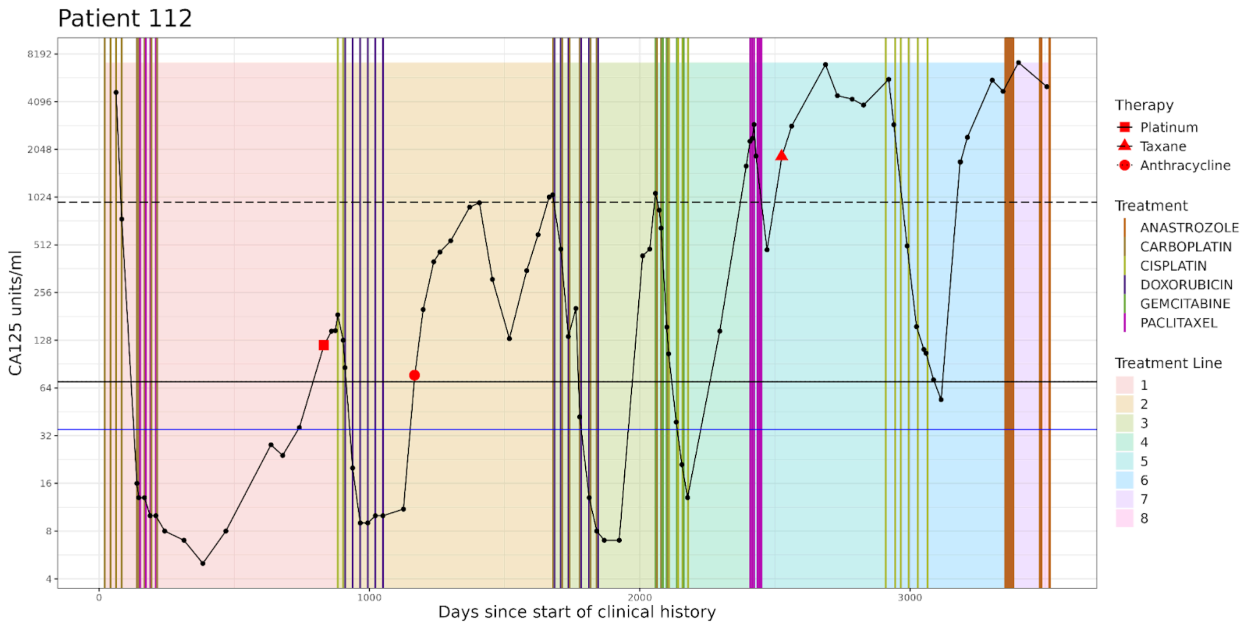

Supplementary Figure 39. Clinical history plot for OV04 patient 112. Blood serum CA125 levels are shown over time as a line graph, and points on the line marked in red show the calculated date of progression. The horizontal lines in black show the CA125 thresholds required for progression, specific to each chemotherapy of interest. Vertical lines denote individual treatment administrations, and background panels indicate which treatment line the administration belongs to. The horizontal bar at 35 units/ml denotes the threshold between ‘normal’ and ‘abnormal’ CA125 readings. In cases where multiple treatments are given on the same day, the treatment date is shifted slightly to show all treatments.

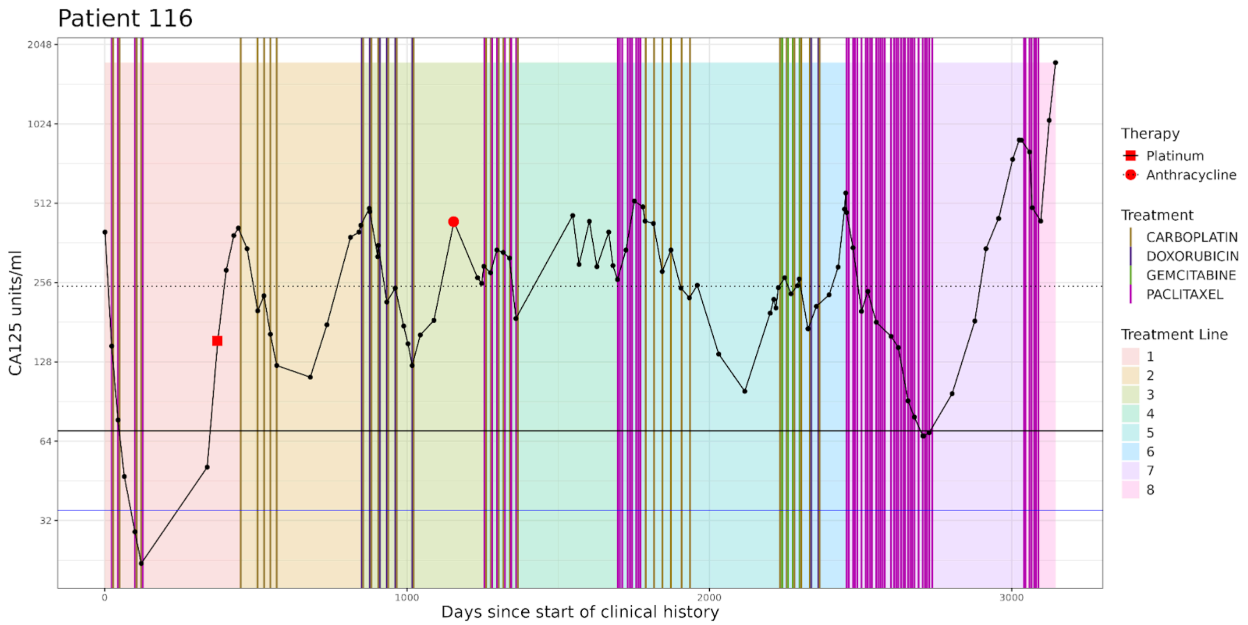

Supplementary Figure 40. Clinical history plot for OV04 patient 116. Blood serum CA125 levels are shown over time as a line graph, and points on the line marked in red show the calculated date of progression. The horizontal lines in black show the CA125 thresholds required for progression, specific to each chemotherapy of interest. Vertical lines denote individual treatment administrations, and background panels indicate which treatment line the administration belongs to. The horizontal bar at 35 units/ml denotes the threshold between ‘normal’ and ‘abnormal’ CA125 readings. In cases where multiple treatments are given on the same day, the treatment date is shifted slightly to show all treatments.

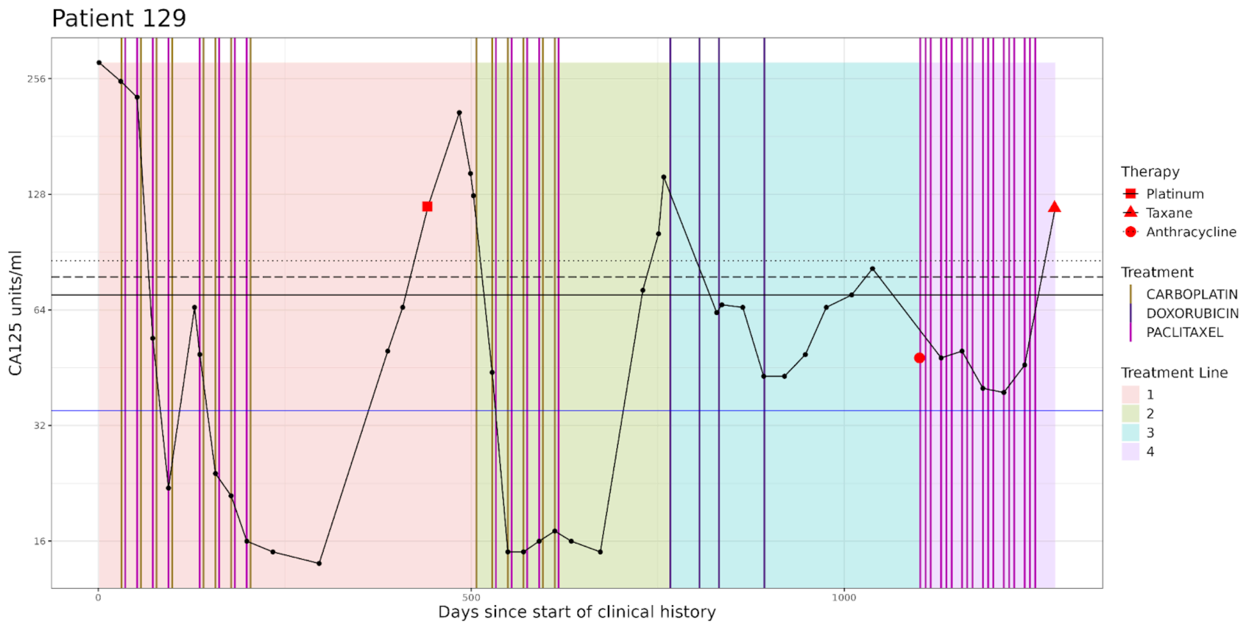

Supplementary Figure 41. Clinical history plot for OV04 patient 129. Blood serum CA125 levels are shown over time as a line graph, and points on the line marked in red show the calculated date of progression. The horizontal lines in black show the CA125 thresholds required for progression, specific to each chemotherapy of interest. Vertical lines denote individual treatment administrations, and background panels indicate which treatment line the administration belongs to. The horizontal bar at 35 units/ml denotes the threshold between ‘normal’ and ‘abnormal’ CA125 readings. In cases where multiple treatments are given on the same day, the treatment date is shifted slightly to show all treatments.

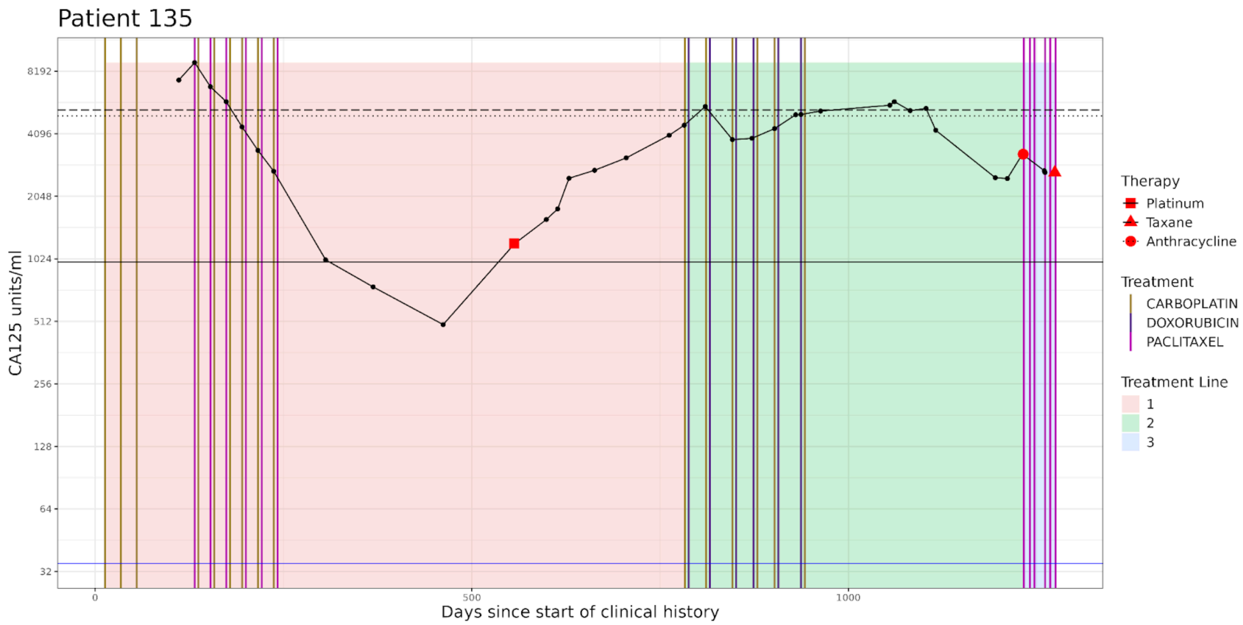

Supplementary Figure 42. Clinical history plot for OV04 patient 135. Blood serum CA125 levels are shown over time as a line graph, and points on the line marked in red show the calculated date of progression. The horizontal lines in black show the CA125 thresholds required for progression, specific to each chemotherapy of interest. Vertical lines denote individual treatment administrations, and background panels indicate which treatment line the administration belongs to. The horizontal bar at 35 units/ml denotes the threshold between 'normal' and 'abnormal' CA125 readings. In cases where multiple treatments are given on the same day, the treatment date is shifted slightly to show all treatments.

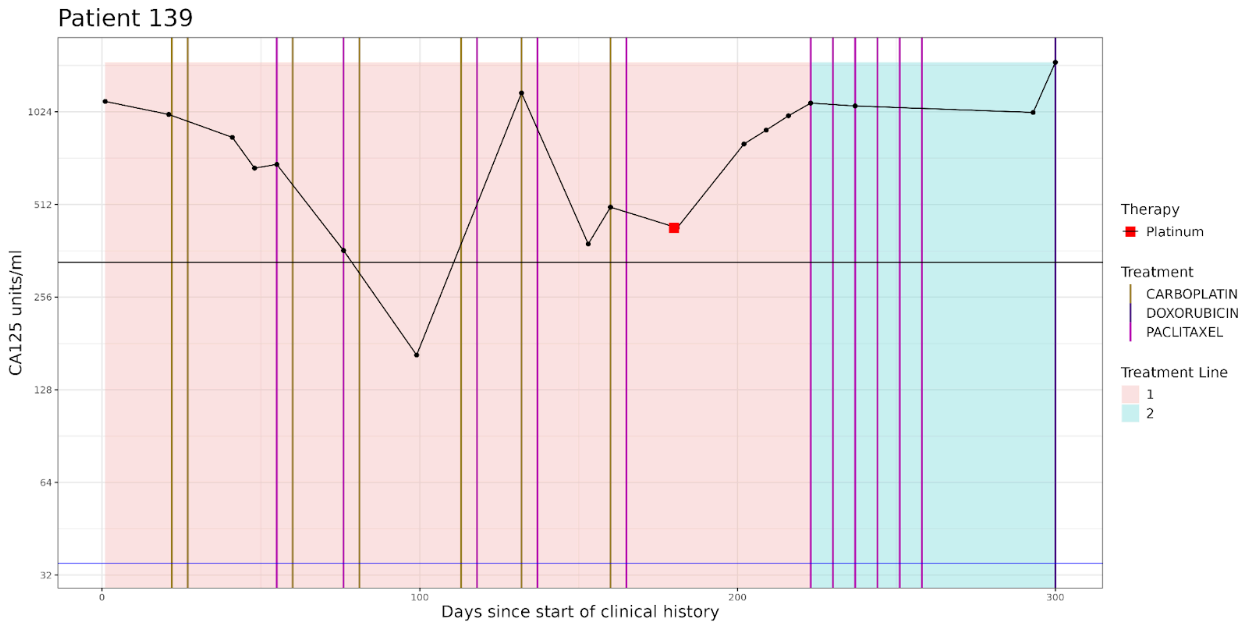

Supplementary Figure 43. Clinical history plot for OV04 patient 139. Blood serum CA125 levels are shown over time as a line graph, and points on the line marked in red show the calculated date of progression. The horizontal lines in black show the CA125 thresholds required for progression, specific to each chemotherapy of interest. Vertical lines denote individual treatment administrations, and background panels indicate which treatment line the administration belongs to. The horizontal bar at 35 units/ml denotes the threshold between ‘normal’ and ‘abnormal’ CA125 readings. In cases where multiple treatments are given on the same day, the treatment date is shifted slightly to show all treatments.

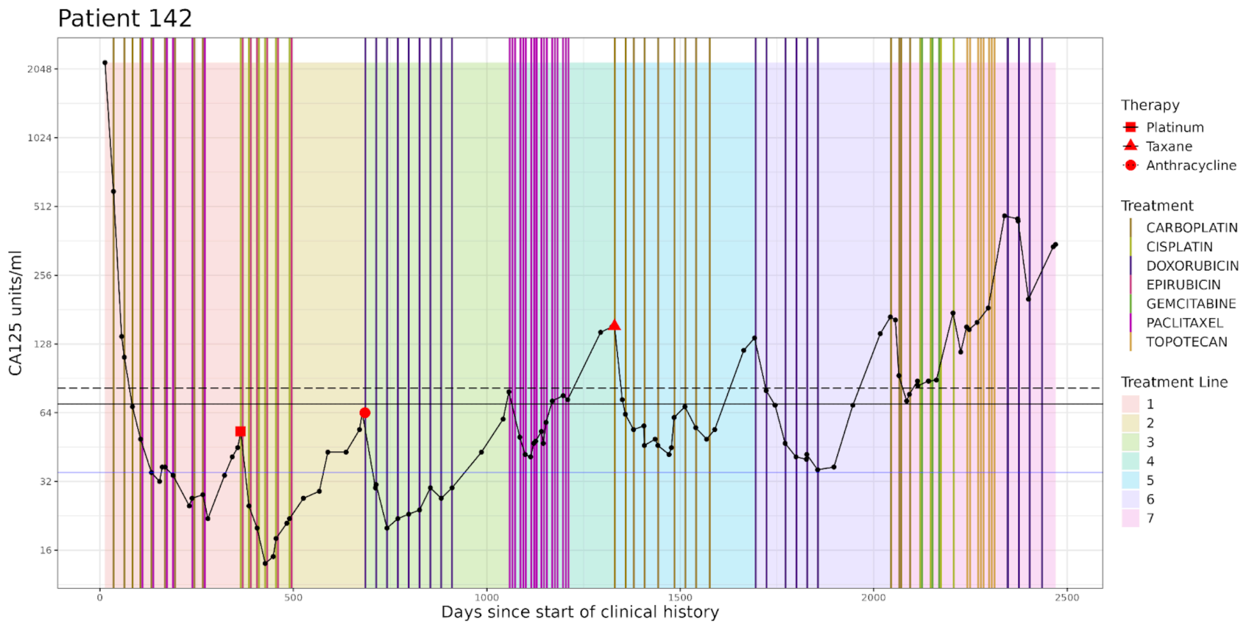

Supplementary Figure 44. Clinical history plot for OV04 patient 142. Blood serum CA125 levels are shown over time as a line graph, and points on the line marked in red show the calculated date of progression. The horizontal lines in black show the CA125 thresholds required for progression, specific to each chemotherapy of interest. Vertical lines denote individual treatment administrations, and background panels indicate which treatment line the administration belongs to. The horizontal bar at 35 units/ml denotes the threshold between ‘normal’ and ‘abnormal’ CA125 readings. In cases where multiple treatments are given on the same day, the treatment date is shifted slightly to show all treatments.

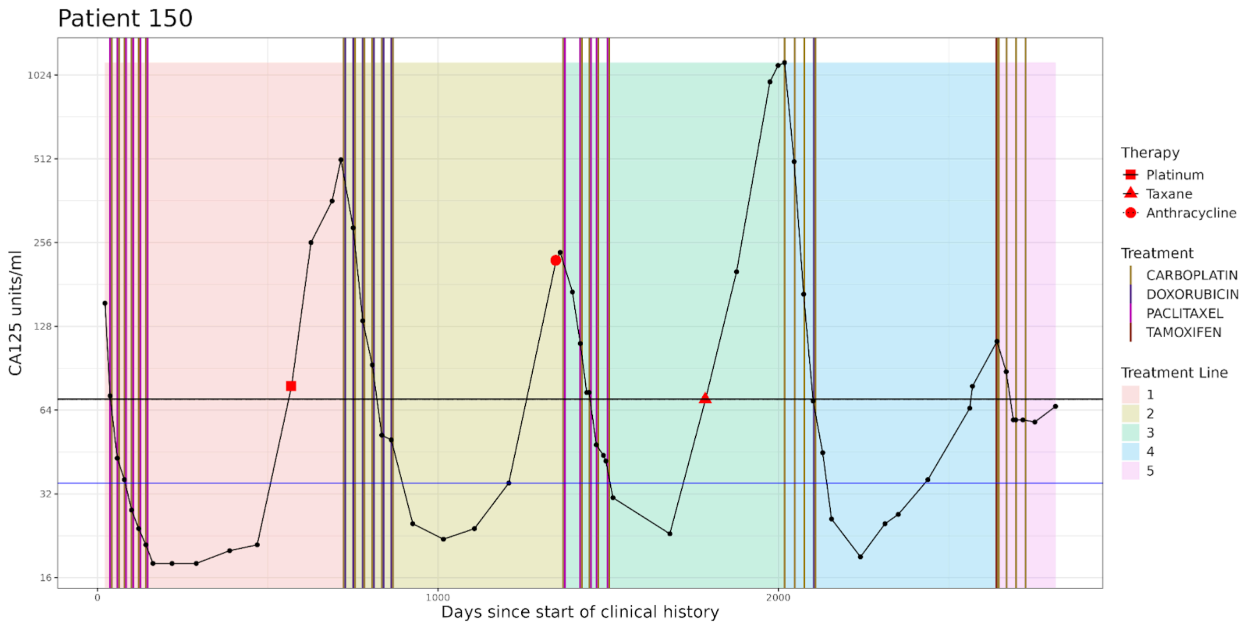

Supplementary Figure 45. Clinical history plot for OV04 patient 150. Blood serum CA125 levels are shown over time as a line graph, and points on the line marked in red show the calculated date of progression. The horizontal lines in black show the CA125 thresholds required for progression, specific to each chemotherapy of interest. Vertical lines denote individual treatment administrations, and background panels indicate which treatment line the administration belongs to. The horizontal bar at 35 units/ml denotes the threshold between 'normal' and 'abnormal' CA125 readings. In cases where multiple treatments are given on the same day, the treatment date is shifted slightly to show all treatments.

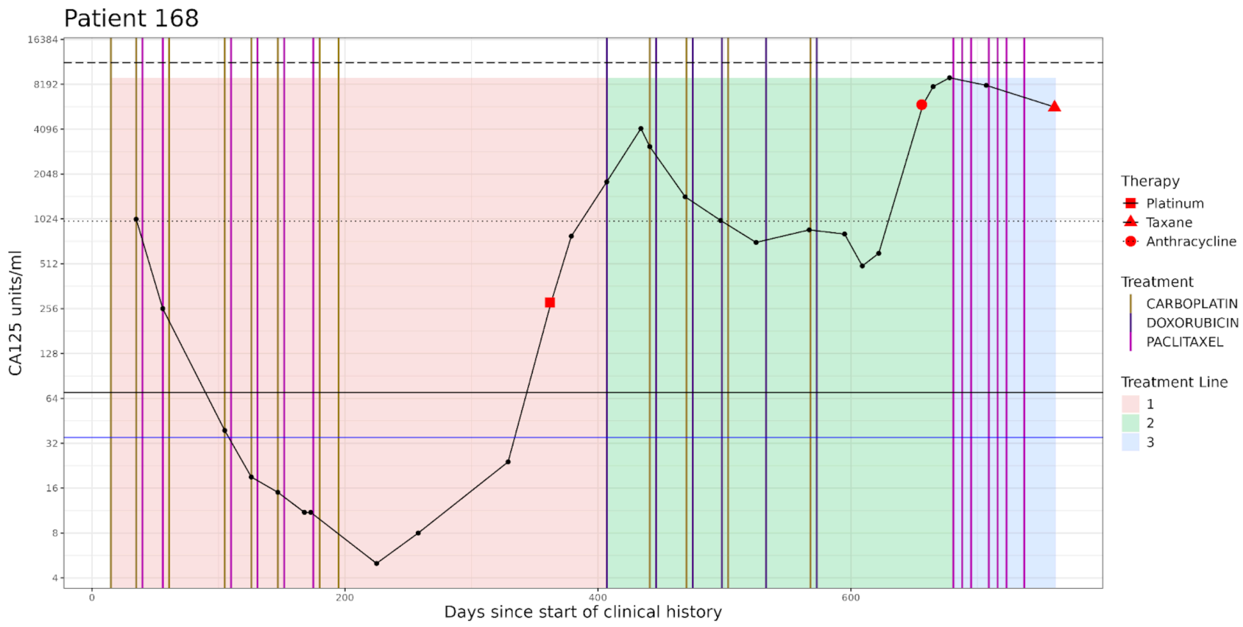

Supplementary Figure 46. Clinical history plot for OV04 patient 168. Blood serum CA125 levels are shown over time as a line graph, and points on the line marked in red show the calculated date of progression. The horizontal lines in black show the CA125 thresholds required for progression, specific to each chemotherapy of interest. Vertical lines denote individual treatment administrations, and background panels indicate which treatment line the administration belongs to. The horizontal bar at 35 units/ml denotes the threshold between 'normal' and 'abnormal' CA125 readings. In cases where multiple treatments are given on the same day, the treatment date is shifted slightly to show all treatments.

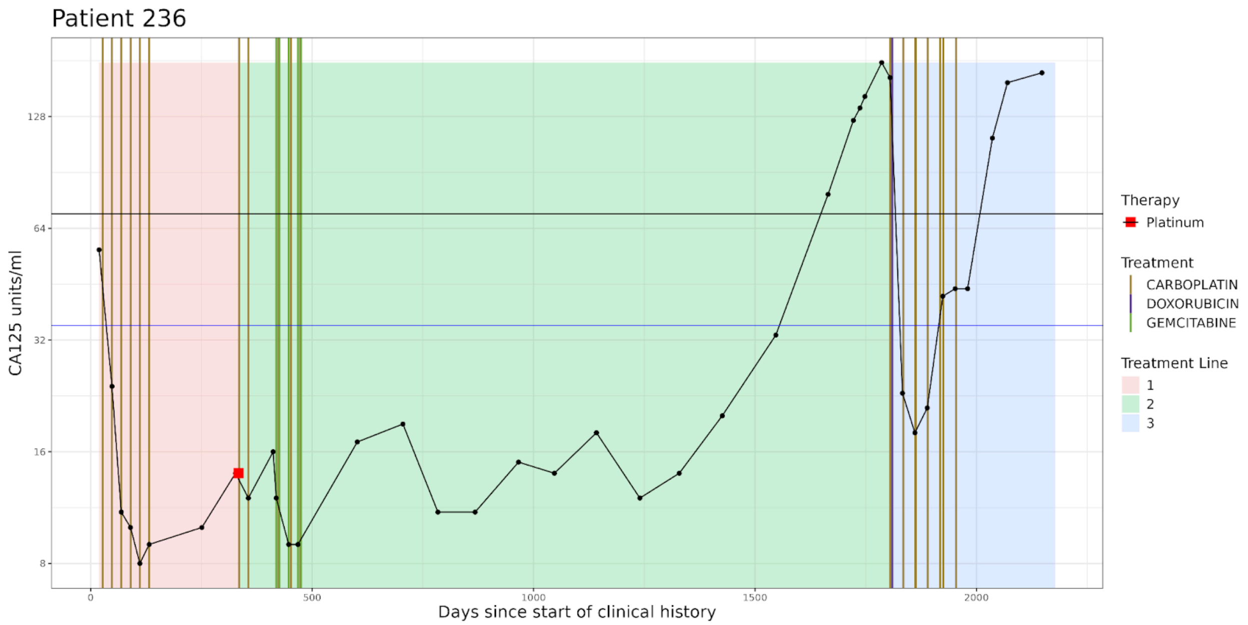

Supplementary Figure 47. Clinical history plot for OV04 patient 236. Blood serum CA125 levels are shown over time as a line graph, and points on the line marked in red show the calculated date of progression. The horizontal lines in black show the CA125 thresholds required for progression, specific to each chemotherapy of interest. Vertical lines denote individual treatment administrations, and background panels indicate which treatment line the administration belongs to. The horizontal bar at 35 units/ml denotes the threshold between ‘normal’ and ‘abnormal’ CA125 readings. In cases where multiple treatments are given on the same day, the treatment date is shifted slightly to show all treatments.

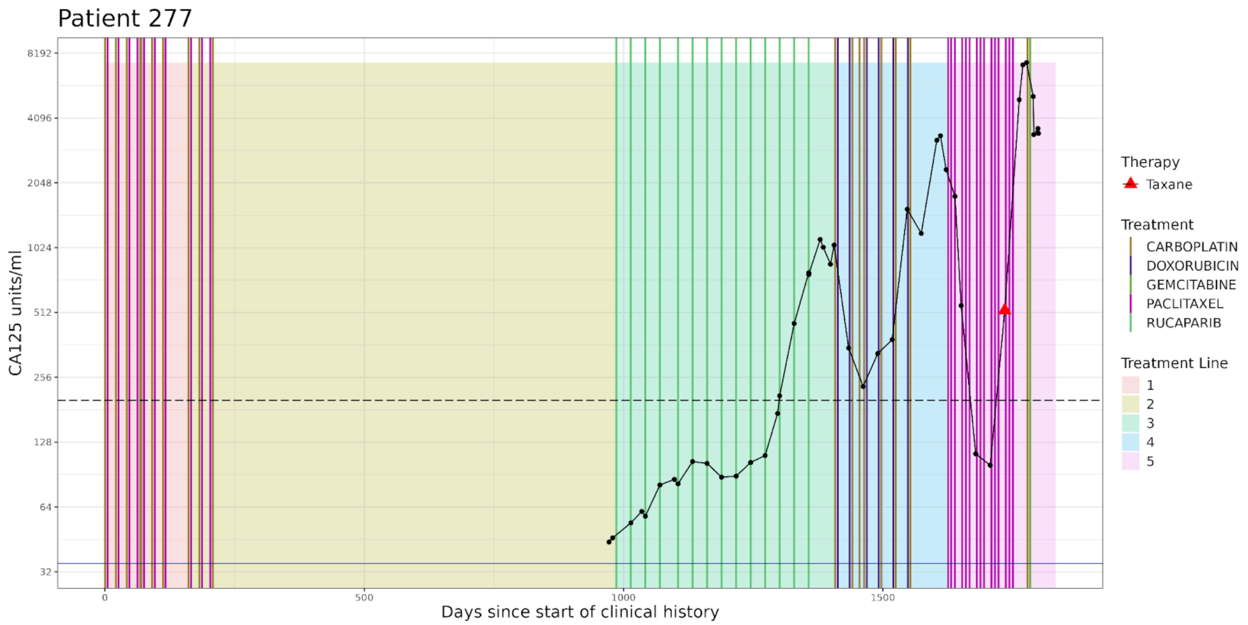

Supplementary Figure 48. Clinical history plot for OV04 patient 277. Blood serum CA125 levels are shown over time as a line graph, and points on the line marked in red show the calculated date of progression. The horizontal lines in black show the CA125 thresholds required for progression, specific to each chemotherapy of interest. Vertical lines denote individual treatment administrations, and background panels indicate which treatment line the administration belongs to. The horizontal bar at 35 units/ml denotes the threshold between 'normal' and 'abnormal' CA125 readings. In cases where multiple treatments are given on the same day, the treatment date is shifted slightly to show all treatments.

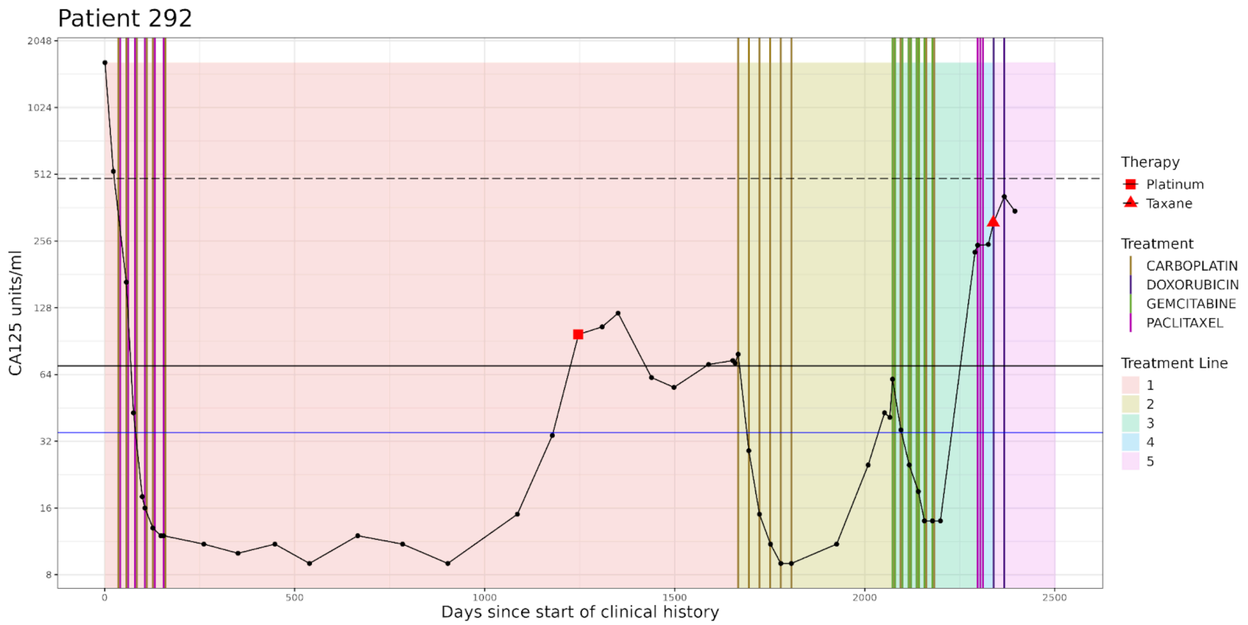

Supplementary Figure 49. Clinical history plot for OV04 patient 292. Blood serum CA125 levels are shown over time as a line graph, and points on the line marked in red show the calculated date of progression. The horizontal lines in black show the CA125 thresholds required for progression, specific to each chemotherapy of interest. Vertical lines denote individual treatment administrations, and background panels indicate which treatment line the administration belongs to. The horizontal bar at 35 units/ml denotes the threshold between ‘normal’ and ‘abnormal’ CA125 readings. In cases where multiple treatments are given on the same day, the treatment date is shifted slightly to show all treatments.

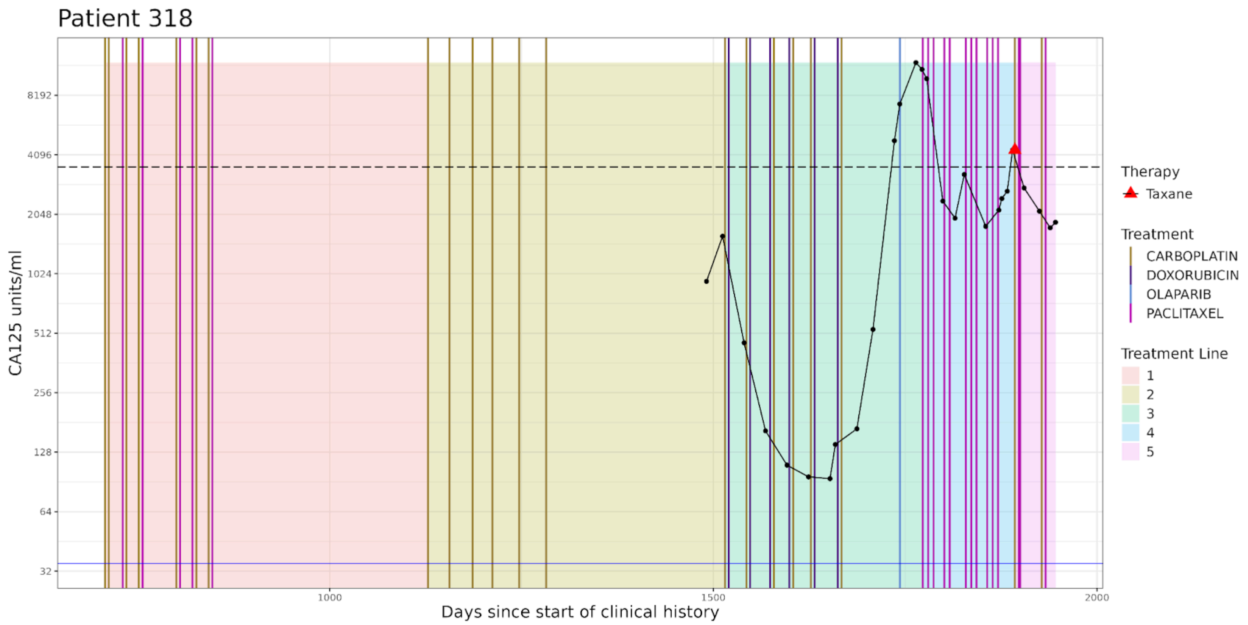

Supplementary Figure 50. Clinical history plot for OV04 patient 318. Blood serum CA125 levels are shown over time as a line graph, and points on the line marked in red show the calculated date of progression. The horizontal lines in black show the CA125 thresholds required for progression, specific to each chemotherapy of interest. Vertical lines denote individual treatment administrations, and background panels indicate which treatment line the administration belongs to. The horizontal bar at 35 units/ml denotes the threshold between ‘normal’ and ‘abnormal’ CA125 readings. In cases where multiple treatments are given on the same day, the treatment date is shifted slightly to show all treatments.

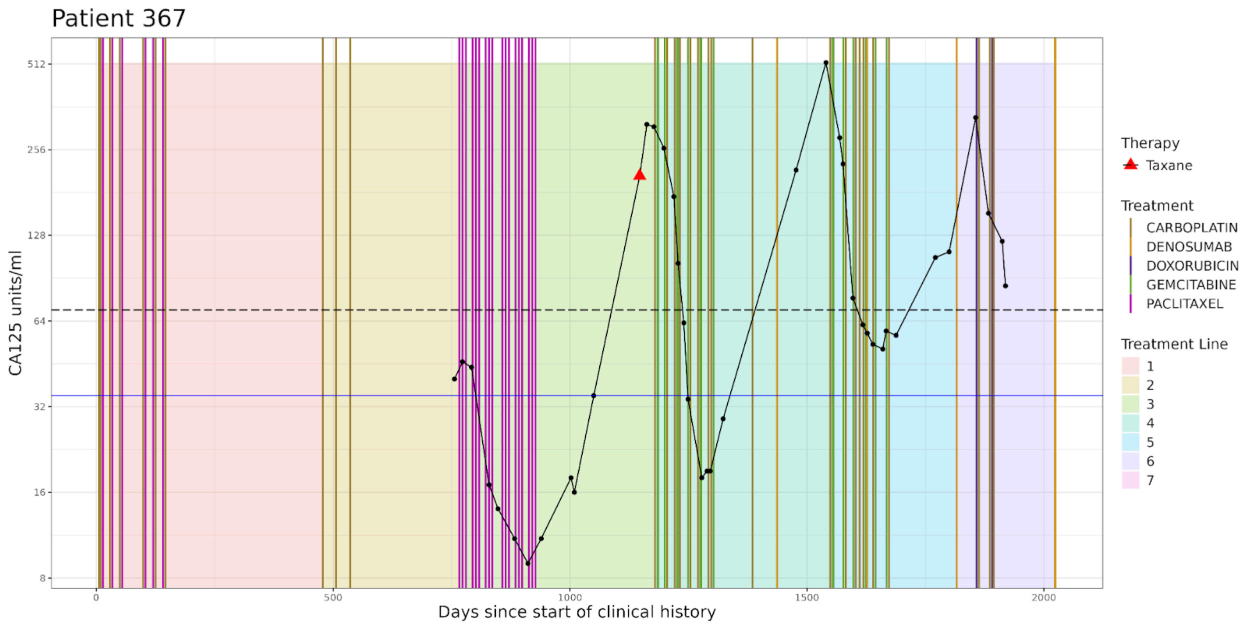

Supplementary Figure 51. Clinical history plot for OV04 patient 367. Blood serum CA125 levels are shown over time as a line graph, and points on the line marked in red show the calculated date of progression. The horizontal lines in black show the CA125 thresholds required for progression, specific to each chemotherapy of interest. Vertical lines denote individual treatment administrations, and background panels indicate which treatment line the administration belongs to. The horizontal bar at 35 units/ml denotes the threshold between ‘normal’ and ‘abnormal’ CA125 readings. In cases where multiple treatments are given on the same day, the treatment date is shifted slightly to show all treatments.

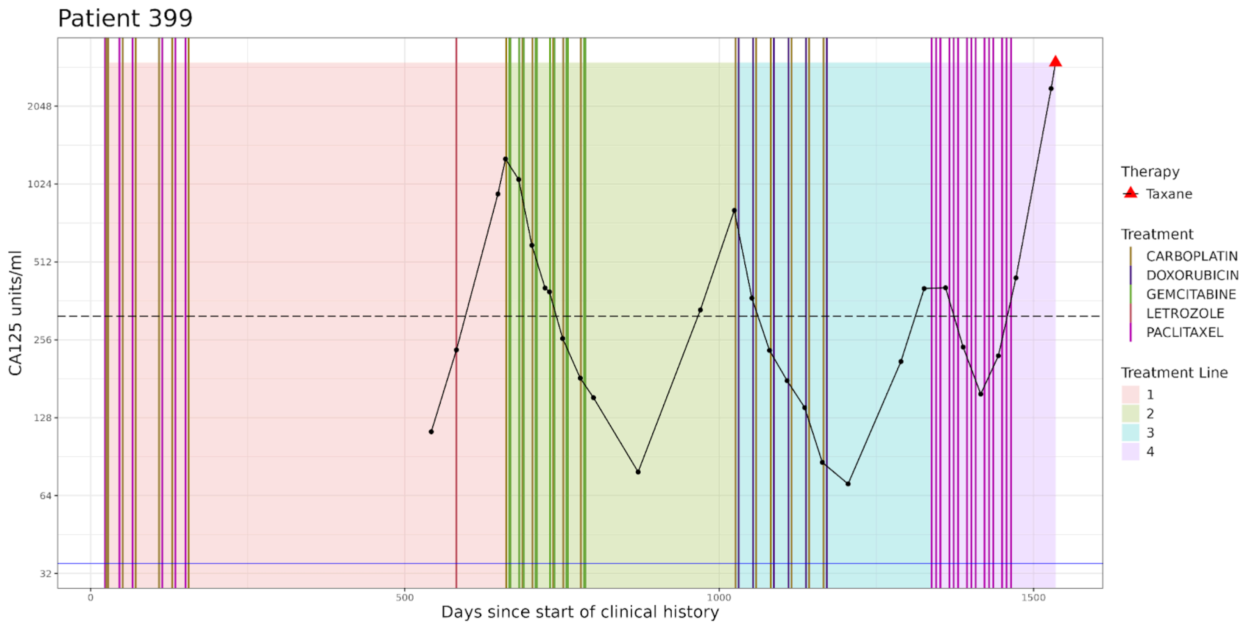

Supplementary Figure 52. Clinical history plot for OV04 patient 399. Blood serum CA125 levels are shown over time as a line graph, and points on the line marked in red show the calculated date of progression. The horizontal lines in black show the CA125 thresholds required for progression, specific to each chemotherapy of interest. Vertical lines denote individual treatment administrations, and background panels indicate which treatment line the administration belongs to. The horizontal bar at 35 units/ml denotes the threshold between 'normal' and 'abnormal' CA125 readings. In cases where multiple treatments are given on the same day, the treatment date is shifted slightly to show all treatments.

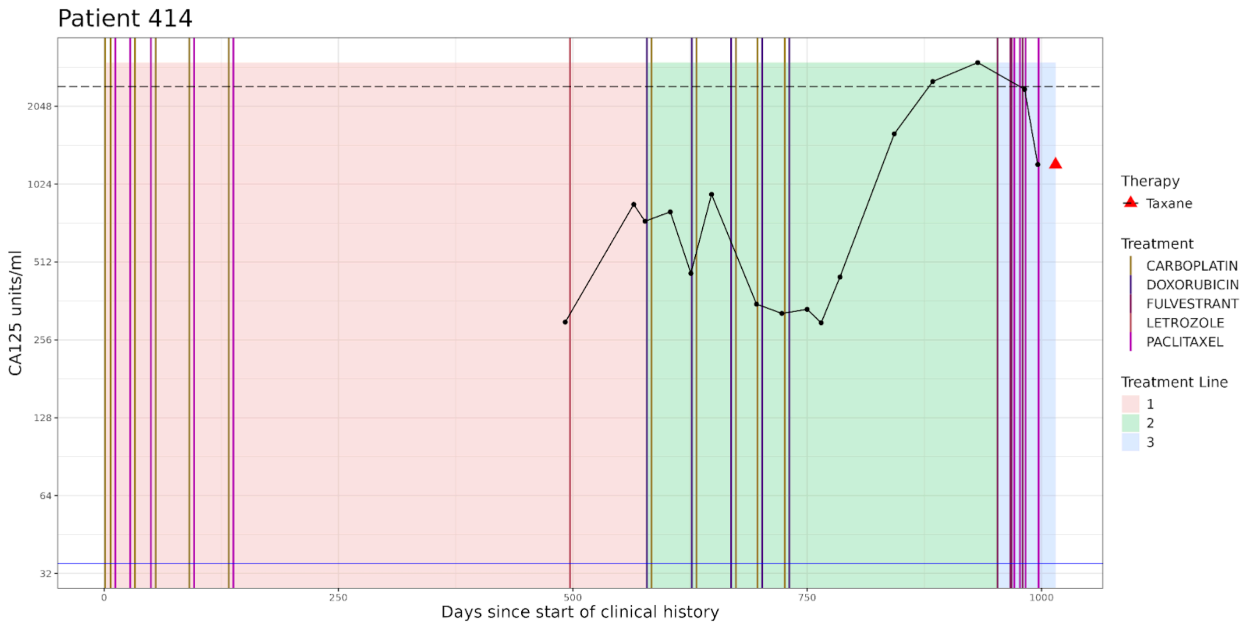

Supplementary Figure 53. Clinical history plot for OV04 patient 414. Blood serum CA125 levels are shown over time as a line graph, and points on the line marked in red show the calculated date of progression. The horizontal lines in black show the CA125 thresholds required for progression, specific to each chemotherapy of interest. Vertical lines denote individual treatment administrations, and background panels indicate which treatment line the administration belongs to. The horizontal bar at 35 units/ml denotes the threshold between ‘normal’ and ‘abnormal’ CA125 readings. In cases where multiple treatments are given on the same day, the treatment date is shifted slightly to show all treatments.

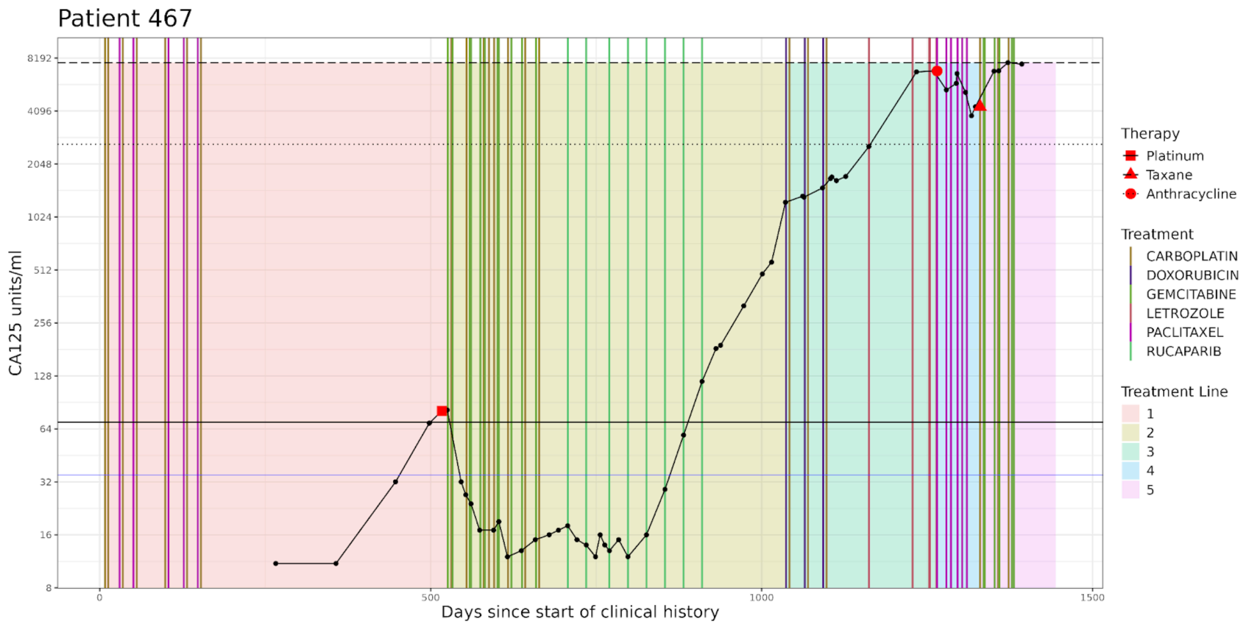

Supplementary Figure 54. Clinical history plot for OV04 patient 467. Blood serum CA125 levels are shown over time as a line graph, and points on the line marked in red show the calculated date of progression. The horizontal lines in black show the CA125 thresholds required for progression, specific to each chemotherapy of interest. Vertical lines denote individual treatment administrations, and background panels indicate which treatment line the administration belongs to. The horizontal bar at 35 units/ml denotes the threshold between ‘normal’ and ‘abnormal’ CA125 readings. In cases where multiple treatments are given on the same day, the treatment date is shifted slightly to show all treatments.

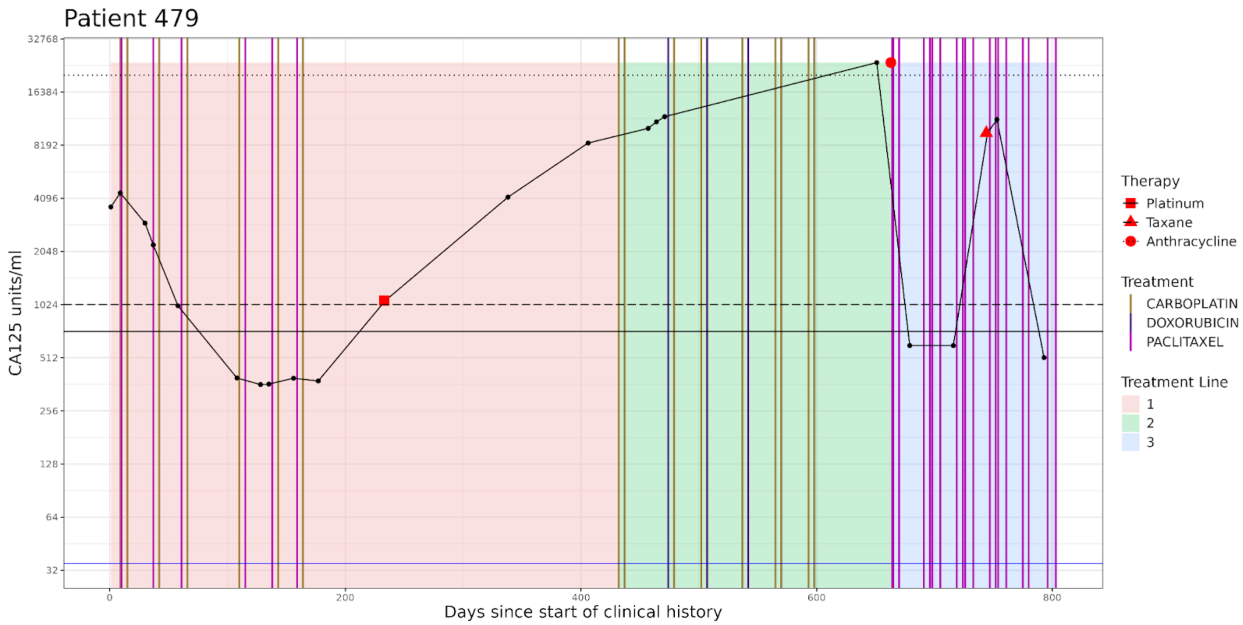

Supplementary Figure 55. Clinical history plot for OV04 patient 479. Blood serum CA125 levels are shown over time as a line graph, and points on the line marked in red show the calculated date of progression. The horizontal lines in black show the CA125 thresholds required for progression, specific to each chemotherapy of interest. Vertical lines denote individual treatment administrations, and background panels indicate which treatment line the administration belongs to. The horizontal bar at 35 units/ml denotes the threshold between ‘normal’ and ‘abnormal’ CA125 readings. In cases where multiple treatments are given on the same day, the treatment date is shifted slightly to show all treatments.

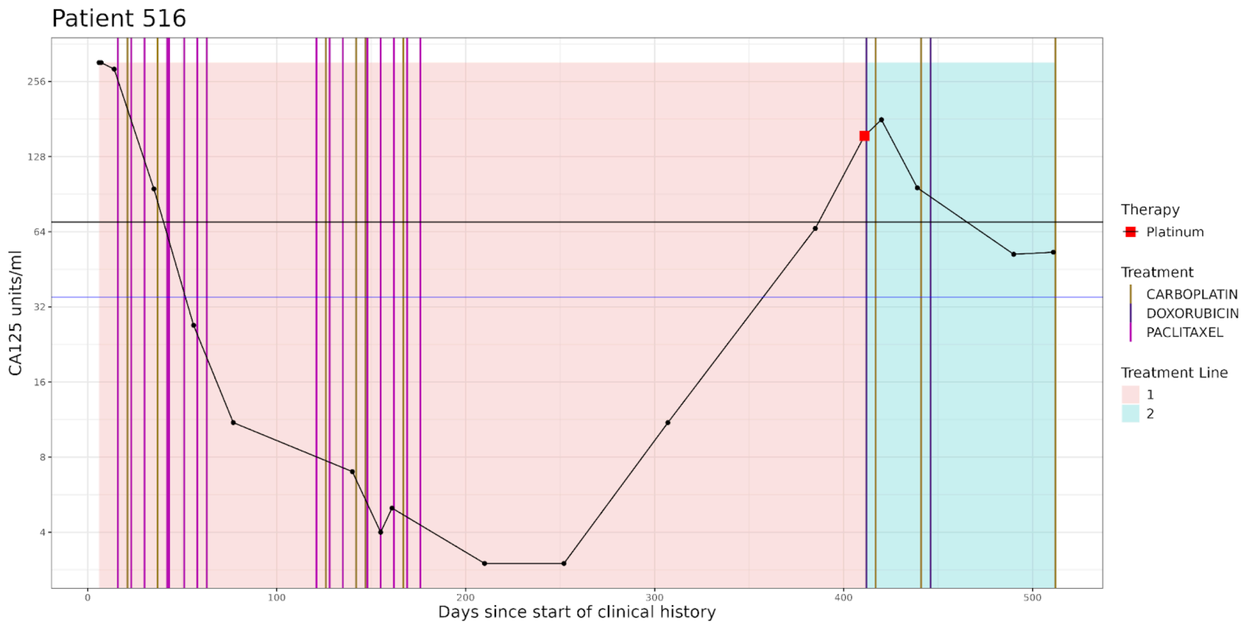

Supplementary Figure 56. Clinical history plot for OV04 patient 516. Blood serum CA125 levels are shown over time as a line graph, and points on the line marked in red show the calculated date of progression. The horizontal lines in black show the CA125 thresholds required for progression, specific to each chemotherapy of interest. Vertical lines denote individual treatment administrations, and background panels indicate which treatment line the administration belongs to. The horizontal bar at 35 units/ml denotes the threshold between 'normal' and 'abnormal' CA125 readings. In cases where multiple treatments are given on the same day, the treatment date is shifted slightly to show all treatments.

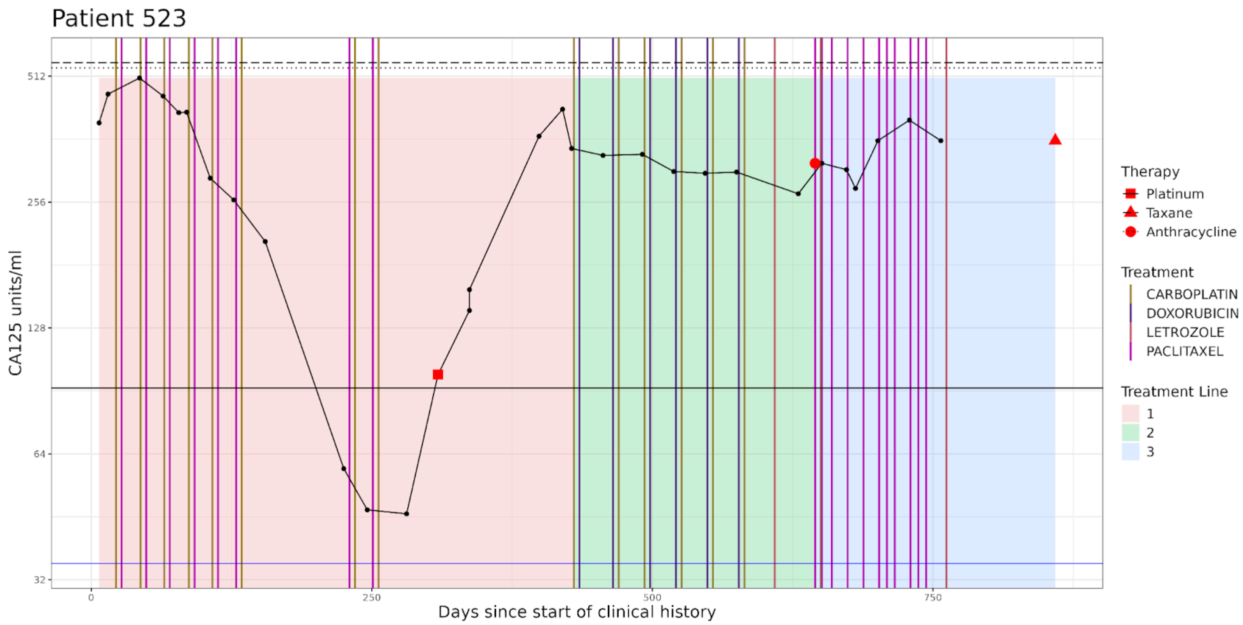

Supplementary Figure 57. Clinical history plot for OV04 patient 523. Blood serum CA125 levels are shown over time as a line graph, and points on the line marked in red show the calculated date of progression. The horizontal lines in black show the CA125 thresholds required for progression, specific to each chemotherapy of interest. Vertical lines denote individual treatment administrations, and background panels indicate which treatment line the administration belongs to. The horizontal bar at 35 units/ml denotes the threshold between ‘normal’ and ‘abnormal’ CA125 readings. In cases where multiple treatments are given on the same day, the treatment date is shifted slightly to show all treatments.

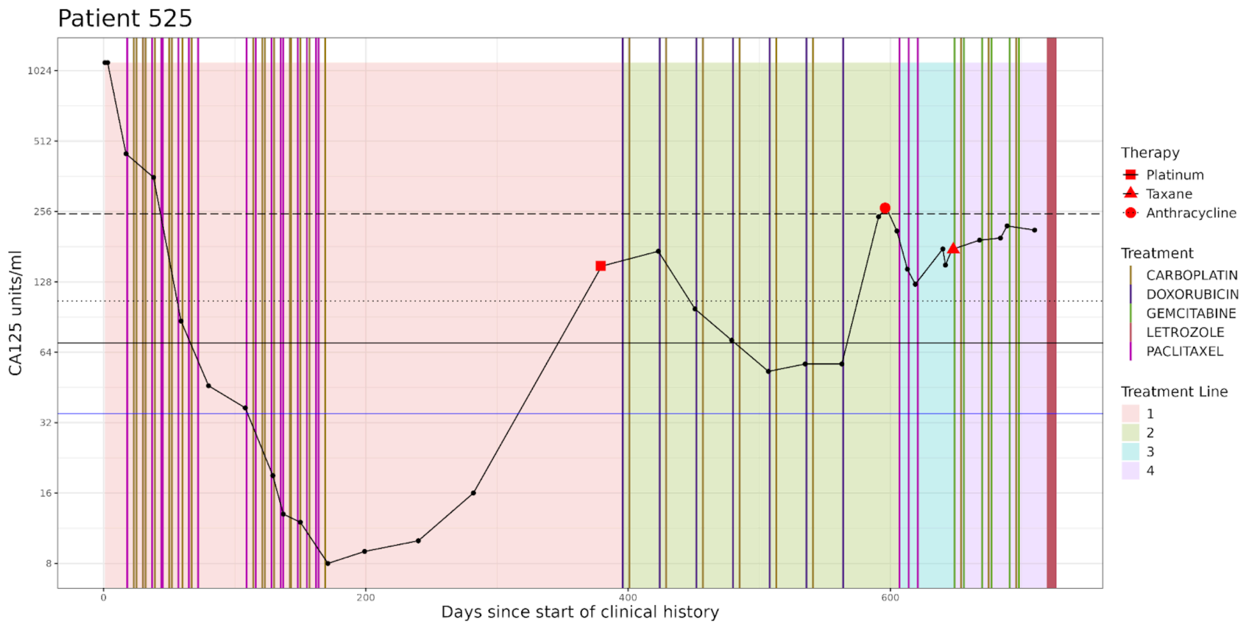

Supplementary Figure 58. Clinical history plot for OV04 patient 525. Blood serum CA125 levels are shown over time as a line graph, and points on the line marked in red show the calculated date of progression. The horizontal lines in black show the CA125 thresholds required for progression, specific to each chemotherapy of interest. Vertical lines denote individual treatment administrations, and background panels indicate which treatment line the administration belongs to. The horizontal bar at 35 units/ml denotes the threshold between 'normal' and 'abnormal' CA125 readings. In cases where multiple treatments are given on the same day, the treatment date is shifted slightly to show all treatments.

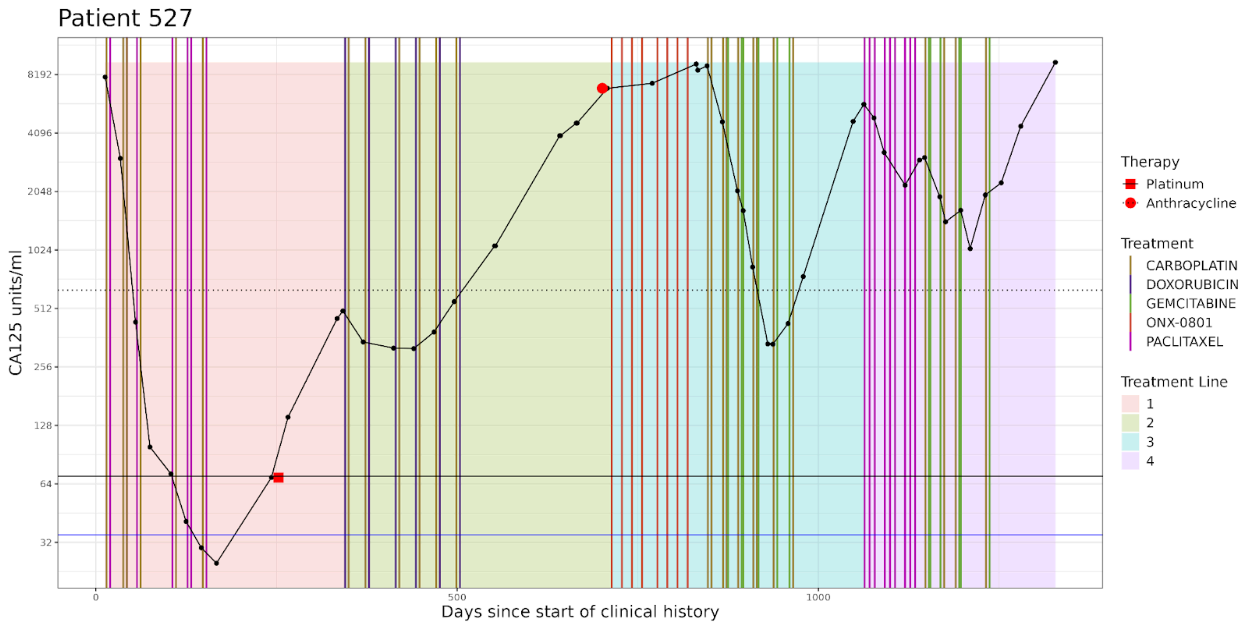

Supplementary Figure 59. Clinical history plot for OV04 patient 527. Blood serum CA125 levels are shown over time as a line graph, and points on the line marked in red show the calculated date of progression. The horizontal lines in black show the CA125 thresholds required for progression, specific to each chemotherapy of interest. Vertical lines denote individual treatment administrations, and background panels indicate which treatment line the administration belongs to. The horizontal bar at 35 units/ml denotes the threshold between ‘normal’ and ‘abnormal’ CA125 readings. In cases where multiple treatments are given on the same day, the treatment date is shifted slightly to show all treatments.

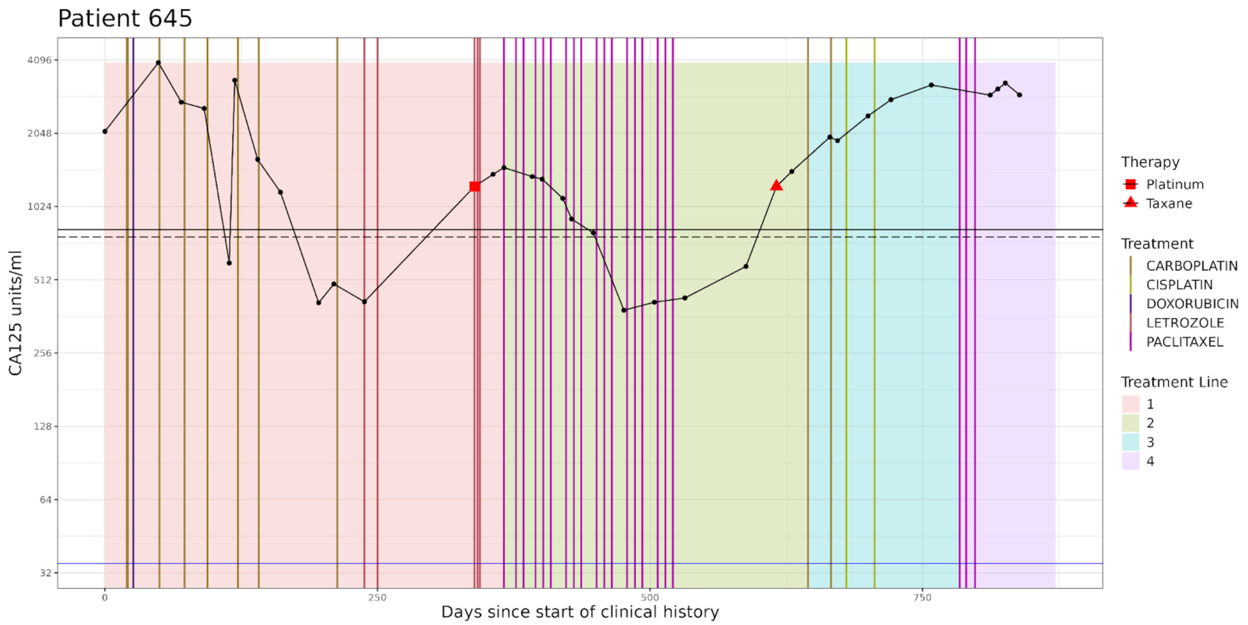

Supplementary Figure 60. Clinical history plot for OV04 patient 645. Blood serum CA125 levels are shown over time as a line graph, and points on the line marked in red show the calculated date of progression. The horizontal lines in black show the CA125 thresholds required for progression, specific to each chemotherapy of interest. Vertical lines denote individual treatment administrations, and background panels indicate which treatment line the administration belongs to. The horizontal bar at 35 units/ml denotes the threshold between 'normal' and 'abnormal' CA125 readings. In cases where multiple treatments are given on the same day, the treatment date is shifted slightly to show all treatments.

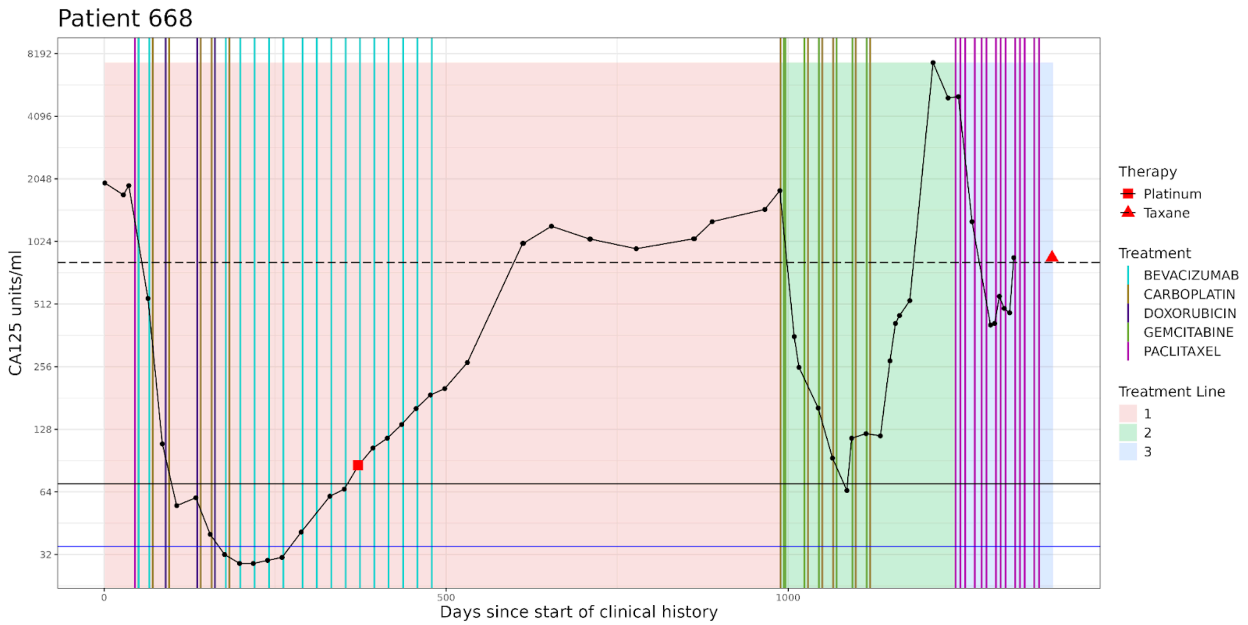

Supplementary Figure 61. Clinical history plot for OV04 patient 668. Blood serum CA125 levels are shown over time as a line graph, and points on the line marked in red show the calculated date of progression. The horizontal lines in black show the CA125 thresholds required for progression, specific to each chemotherapy of interest. Vertical lines denote individual treatment administrations, and background panels indicate which treatment line the administration belongs to. The horizontal bar at 35 units/ml denotes the threshold between ‘normal’ and ‘abnormal’ CA125 readings. In cases where multiple treatments are given on the same day, the treatment date is shifted slightly to show all treatments.

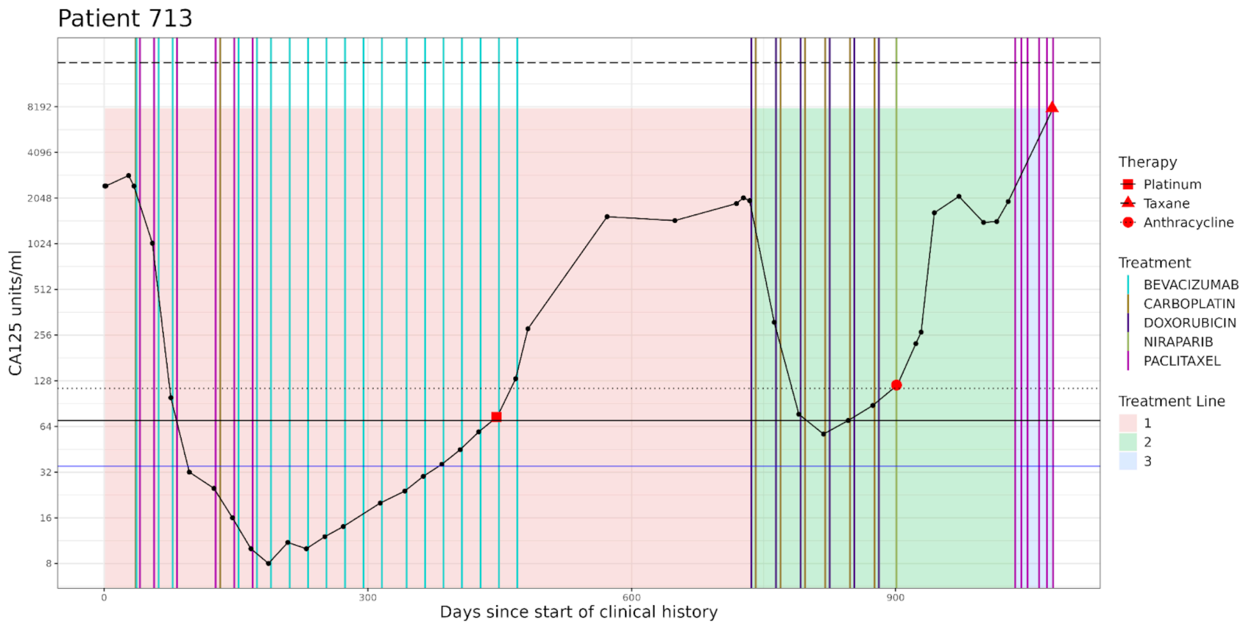

Supplementary Figure 62. Clinical history plot for OV04 patient 713. Blood serum CA125 levels are shown over time as a line graph, and points on the line marked in red show the calculated date of progression. The horizontal lines in black show the CA125 thresholds required for progression, specific to each chemotherapy of interest. Vertical lines denote individual treatment administrations, and background panels indicate which treatment line the administration belongs to. The horizontal bar at 35 units/ml denotes the threshold between ‘normal’ and ‘abnormal’ CA125 readings. In cases where multiple treatments are given on the same day, the treatment date is shifted slightly to show all treatments.

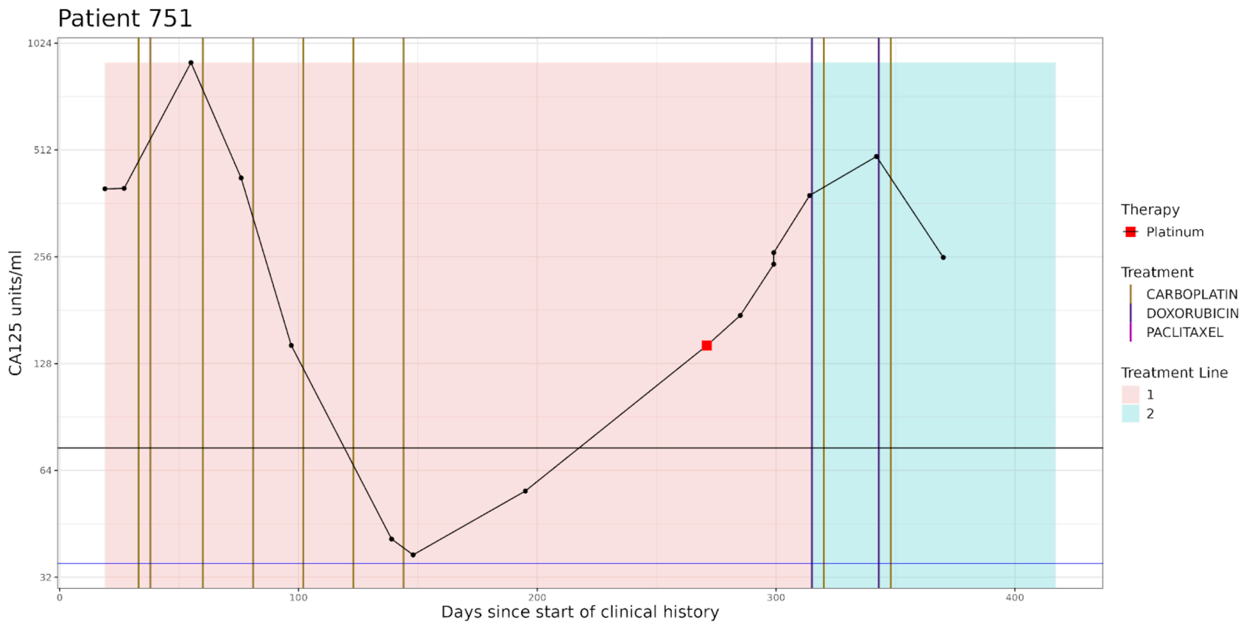

Supplementary Figure 63. Clinical history plot for OV04 patient 751. Blood serum CA125 levels are shown over time as a line graph, and points on the line marked in red show the calculated date of progression. The horizontal lines in black show the CA125 thresholds required for progression, specific to each chemotherapy of interest. Vertical lines denote individual treatment administrations, and background panels indicate which treatment line the administration belongs to. The horizontal bar at 35 units/ml denotes the threshold between 'normal' and 'abnormal' CA125 readings. In cases where multiple treatments are given on the same day, the treatment date is shifted slightly to show all treatments.

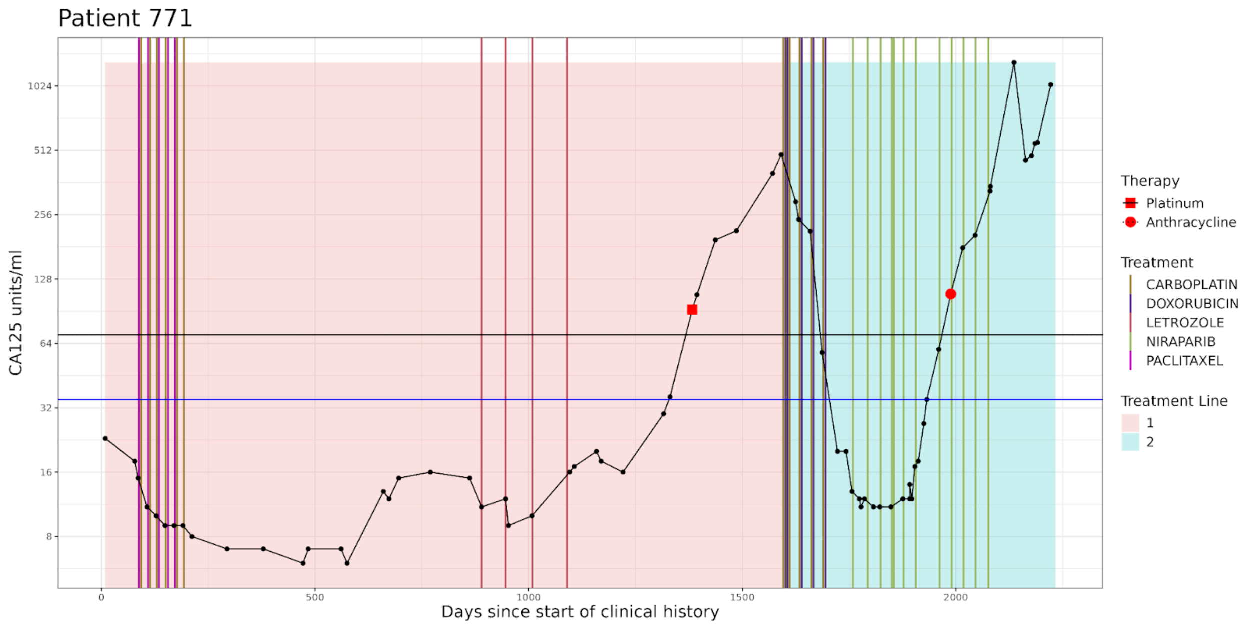

Supplementary Figure 64. Clinical history plot for OV04 patient 771. Blood serum CA125 levels are shown over time as a line graph, and points on the line marked in red show the calculated date of progression. The horizontal lines in black show the CA125 thresholds required for progression, specific to each chemotherapy of interest. Vertical lines denote individual treatment administrations, and background panels indicate which treatment line the administration belongs to. The horizontal bar at 35 units/ml denotes the threshold between ‘normal’ and ‘abnormal’ CA125 readings. In cases where multiple treatments are given on the same day, the treatment date is shifted slightly to show all treatments.

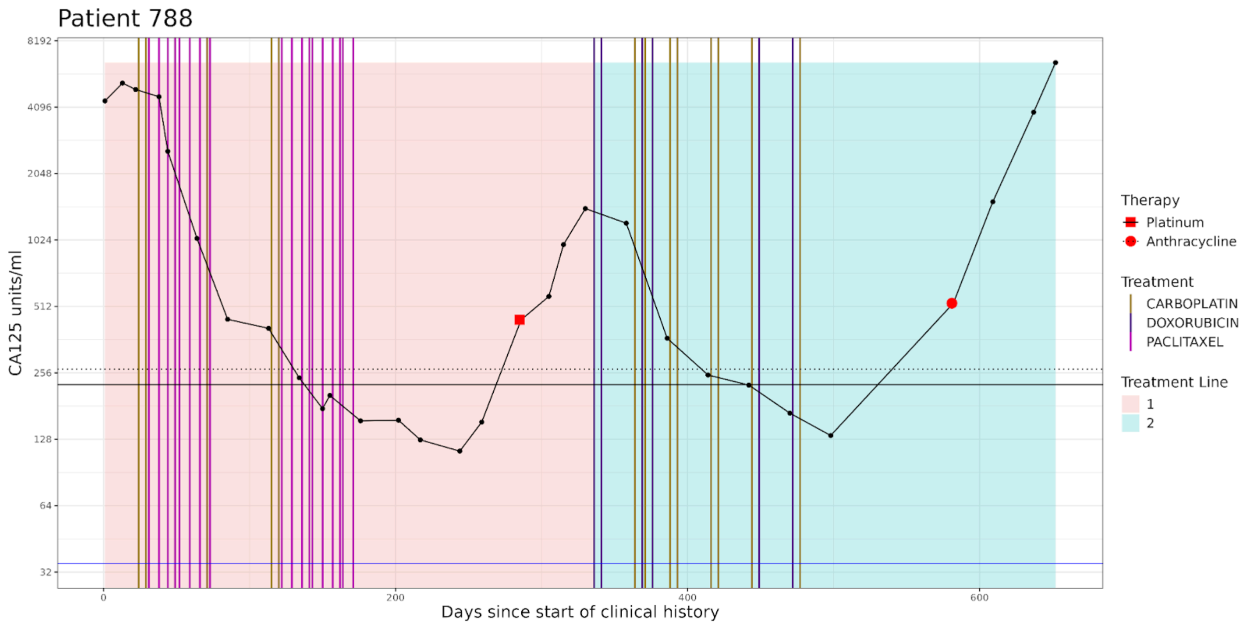

Supplementary Figure 65. Clinical history plot for OV04 patient 788. Blood serum CA125 levels are shown over time as a line graph, and points on the line marked in red show the calculated date of progression. The horizontal lines in black show the CA125 thresholds required for progression, specific to each chemotherapy of interest. Vertical lines denote individual treatment administrations, and background panels indicate which treatment line the administration belongs to. The horizontal bar at 35 units/ml denotes the threshold between ‘normal’ and ‘abnormal’ CA125 readings. In cases where multiple treatments are given on the same day, the treatment date is shifted slightly to show all treatments.

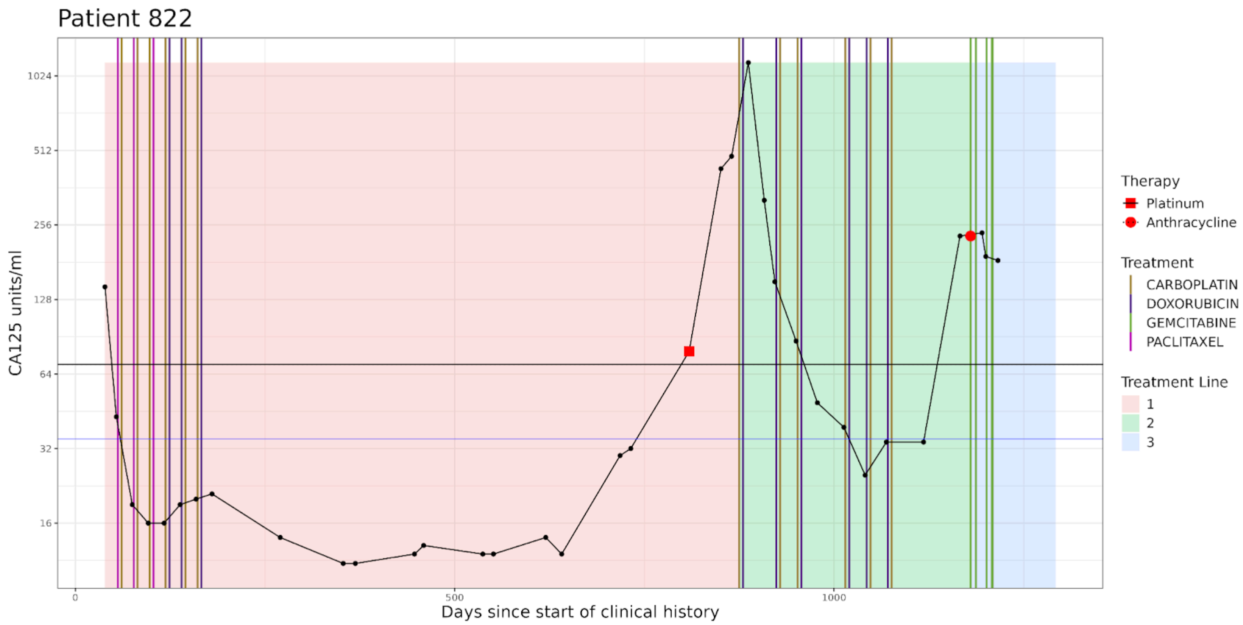

Supplementary Figure 66. Clinical history plot for OV04 patient 822. Blood serum CA125 levels are shown over time as a line graph, and points on the line marked in red show the calculated date of progression. The horizontal lines in black show the CA125 thresholds required for progression, specific to each chemotherapy of interest. Vertical lines denote individual treatment administrations, and background panels indicate which treatment line the administration belongs to. The horizontal bar at 35 units/ml denotes the threshold between 'normal' and 'abnormal' CA125 readings. In cases where multiple treatments are given on the same day, the treatment date is shifted slightly to show all treatments.

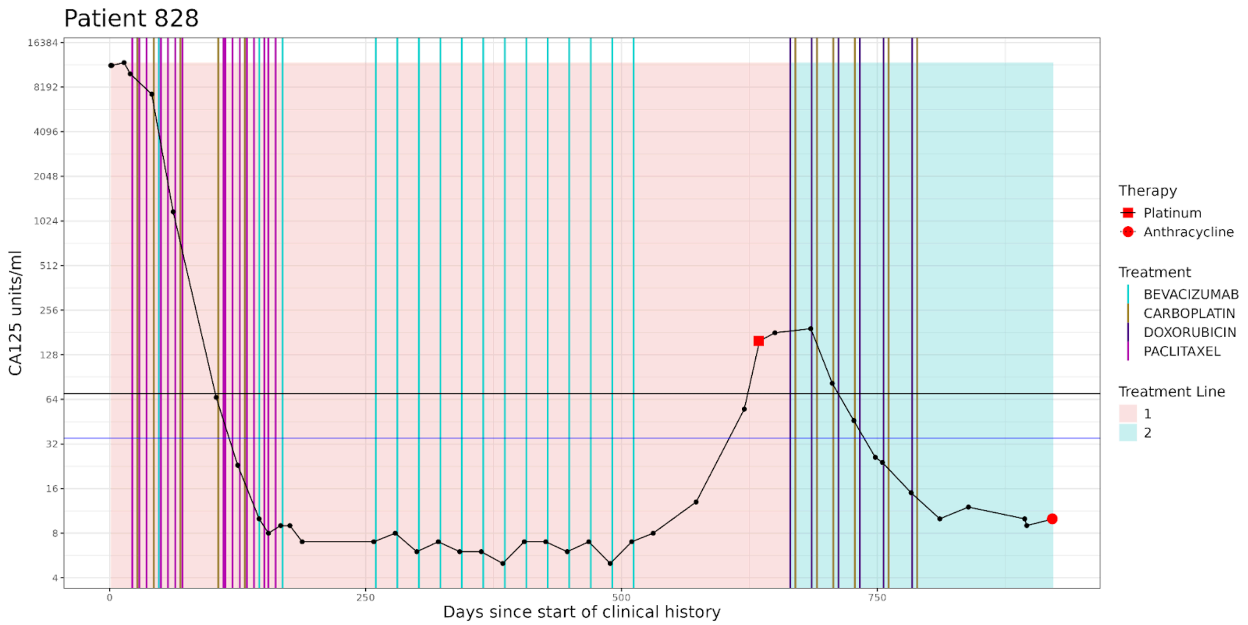

Supplementary Figure 67. Clinical history plot for OV04 patient 828. Blood serum CA125 levels are shown over time as a line graph, and points on the line marked in red show the calculated date of progression. The horizontal lines in black show the CA125 thresholds required for progression, specific to each chemotherapy of interest. Vertical lines denote individual treatment administrations, and background panels indicate which treatment line the administration belongs to. The horizontal bar at 35 units/ml denotes the threshold between ‘normal’ and ‘abnormal’ CA125 readings. In cases where multiple treatments are given on the same day, the treatment date is shifted slightly to show all treatments.

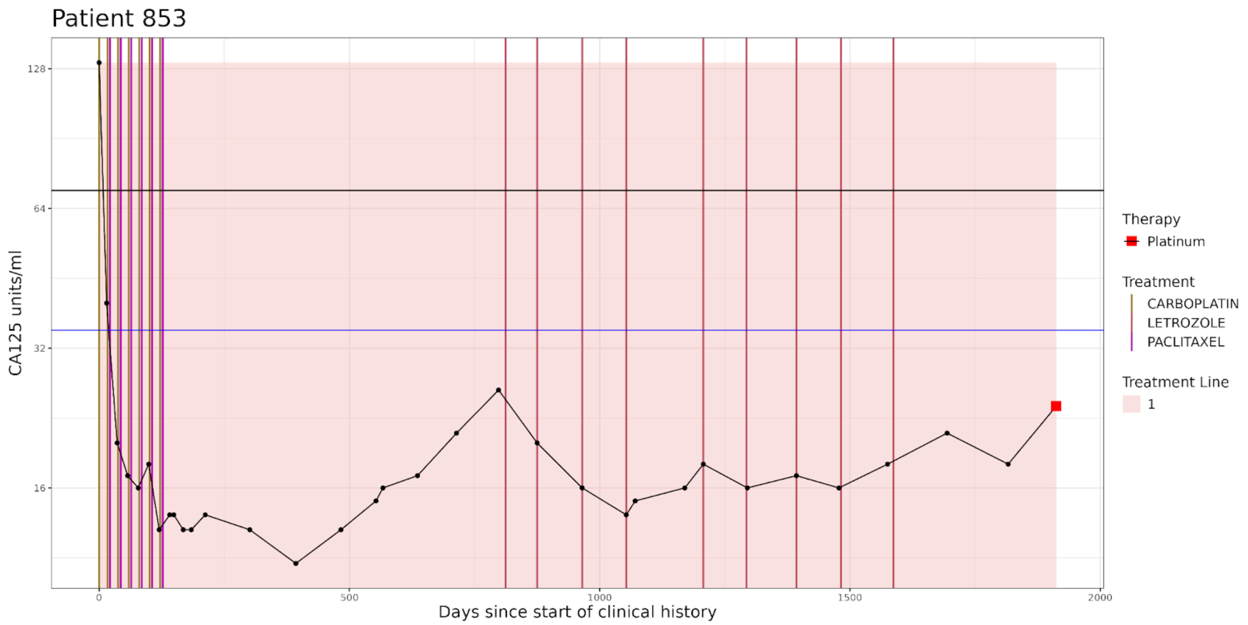

Supplementary Figure 68. Clinical history plot for OV04 patient 853. Blood serum CA125 levels are shown over time as a line graph, and points on the line marked in red show the calculated date of progression. The horizontal lines in black show the CA125 thresholds required for progression, specific to each chemotherapy of interest. Vertical lines denote individual treatment administrations, and background panels indicate which treatment line the administration belongs to. The horizontal bar at 35 units/ml denotes the threshold between 'normal' and 'abnormal' CA125 readings. In cases where multiple treatments are given on the same day, the treatment date is shifted slightly to show all treatments.

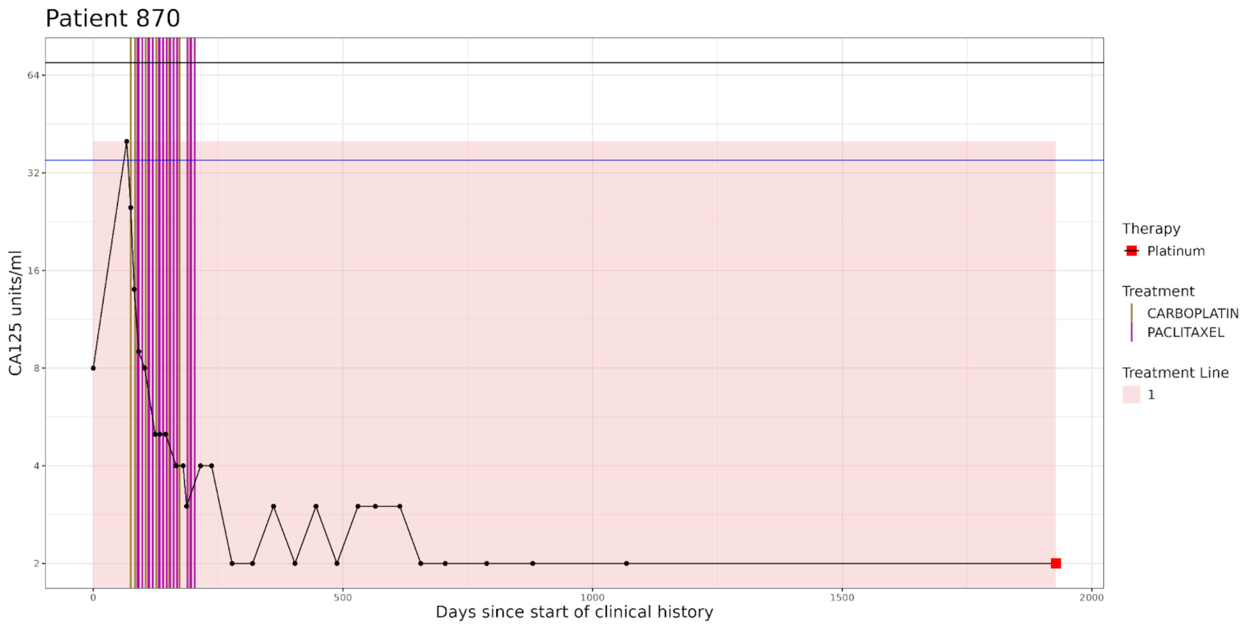

Supplementary Figure 69. Clinical history plot for OV04 patient 870. Blood serum CA125 levels are shown over time as a line graph, and points on the line marked in red show the calculated date of progression. The horizontal lines in black show the CA125 thresholds required for progression, specific to each chemotherapy of interest. Vertical lines denote individual treatment administrations, and background panels indicate which treatment line the administration belongs to. The horizontal bar at 35 units/ml denotes the threshold between ‘normal’ and ‘abnormal’ CA125 readings. In cases where multiple treatments are given on the same day, the treatment date is shifted slightly to show all treatments.

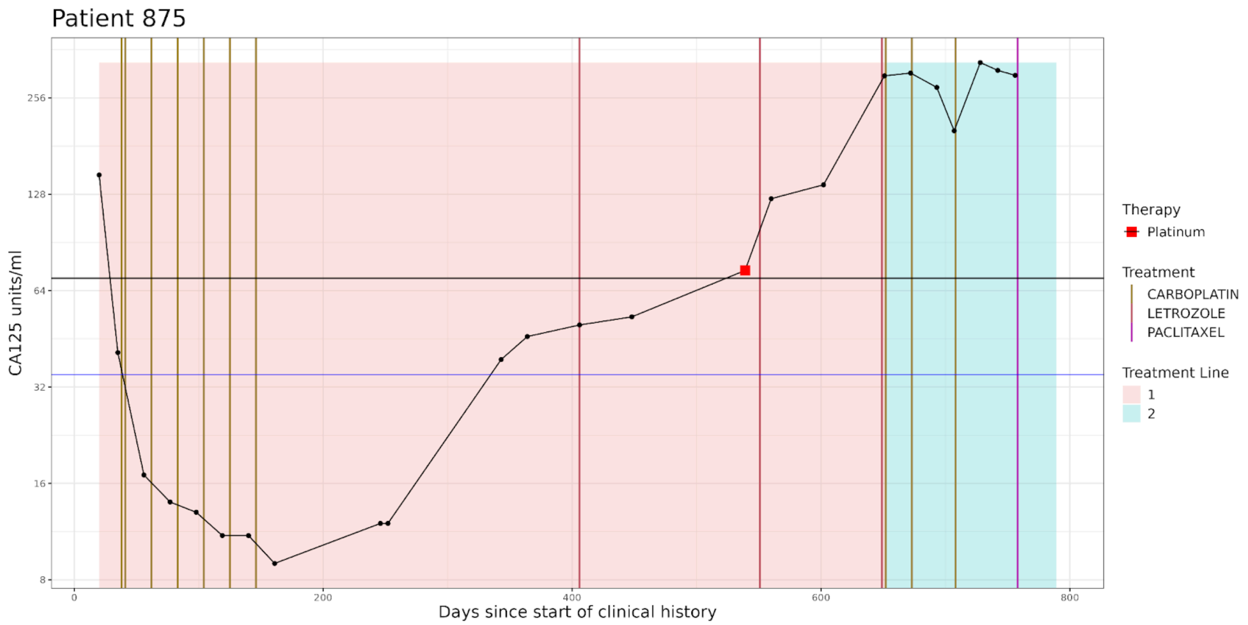

Supplementary Figure 70. Clinical history plot for OV04 patient 875. Blood serum CA125 levels are shown over time as a line graph, and points on the line marked in red show the calculated date of progression. The horizontal lines in black show the CA125 thresholds required for progression, specific to each chemotherapy of interest. Vertical lines denote individual treatment administrations, and background panels indicate which treatment line the administration belongs to. The horizontal bar at 35 units/ml denotes the threshold between ‘normal’ and ‘abnormal’ CA125 readings. In cases where multiple treatments are given on the same day, the treatment date is shifted slightly to show all treatments.

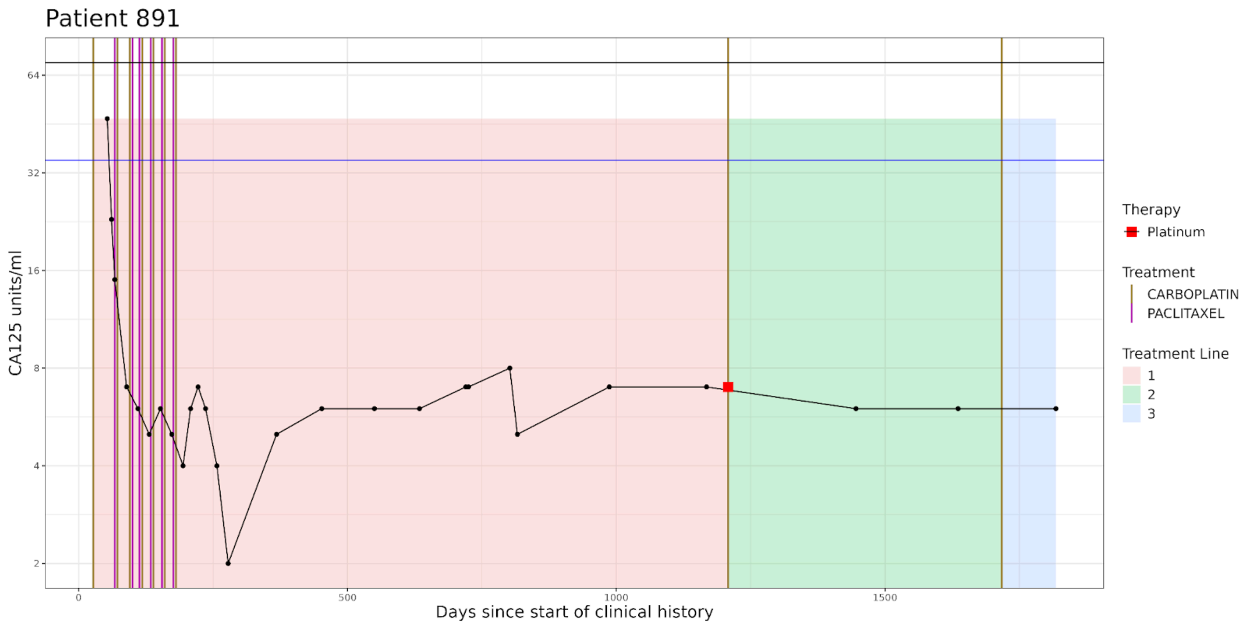

Supplementary Figure 71. Clinical history plot for OV04 patient 891. Blood serum CA125 levels are shown over time as a line graph, and points on the line marked in red show the calculated date of progression. The horizontal lines in black show the CA125 thresholds required for progression, specific to each chemotherapy of interest. Vertical lines denote individual treatment administrations, and background panels indicate which treatment line the administration belongs to. The horizontal bar at 35 units/ml denotes the threshold between 'normal' and 'abnormal' CA125 readings. In cases where multiple treatments are given on the same day, the treatment date is shifted slightly to show all treatments.

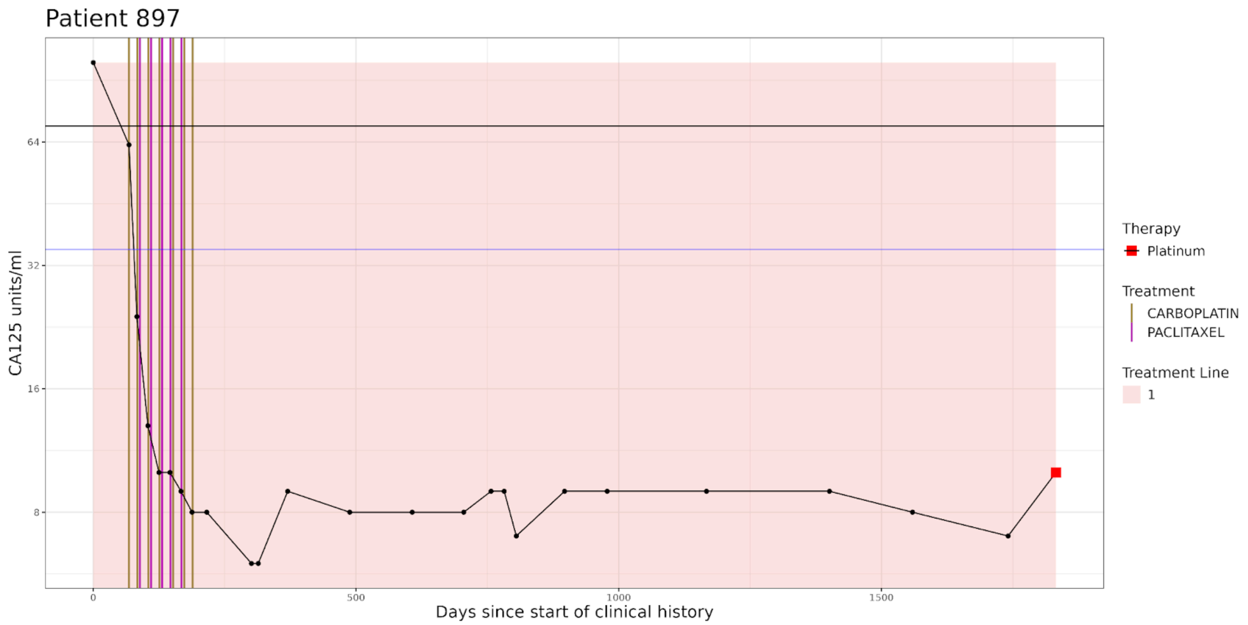

Supplementary Figure 72. Clinical history plot for OV04 patient 897. Blood serum CA125 levels are shown over time as a line graph, and points on the line marked in red show the calculated date of progression. The horizontal lines in black show the CA125 thresholds required for progression, specific to each chemotherapy of interest. Vertical lines denote individual treatment administrations, and background panels indicate which treatment line the administration belongs to. The horizontal bar at 35 units/ml denotes the threshold between ‘normal’ and ‘abnormal’ CA125 readings. In cases where multiple treatments are given on the same day, the treatment date is shifted slightly to show all treatments.

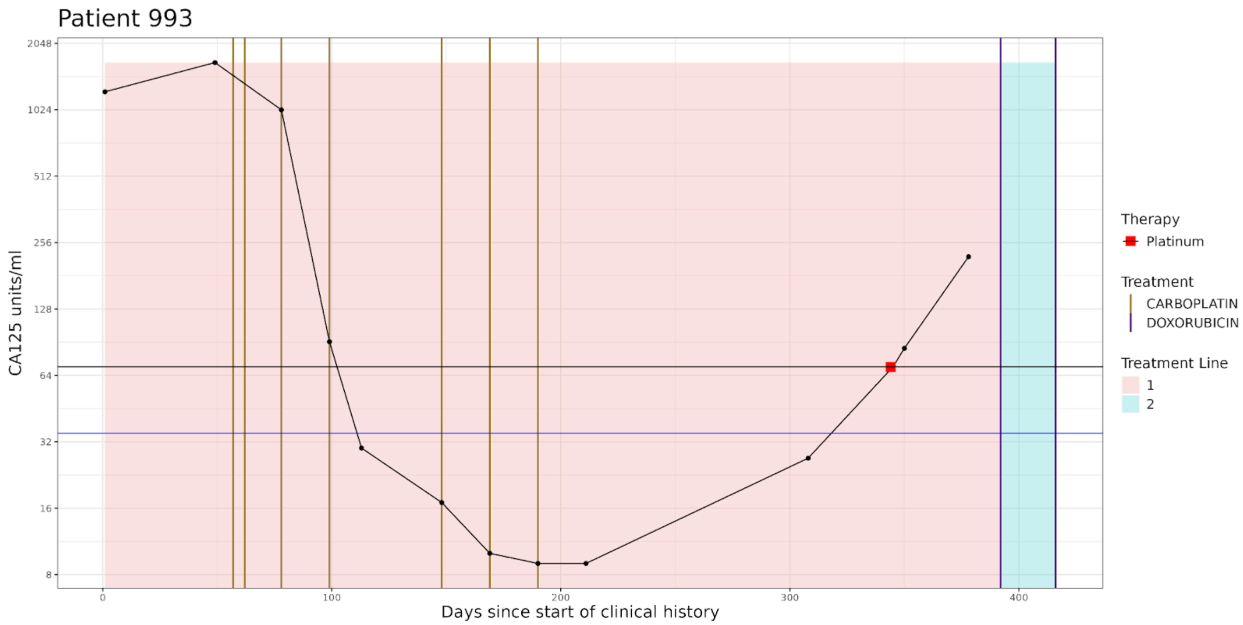

Supplementary Figure 73. Clinical history plot for OV04 patient 993. Blood serum CA125 levels are shown over time as a line graph, and points on the line marked in red show the calculated date of progression. The horizontal lines in black show the CA125 thresholds required for progression, specific to each chemotherapy of interest. Vertical lines denote individual treatment administrations, and background panels indicate which treatment line the administration belongs to. The horizontal bar at 35 units/ml denotes the threshold between 'normal' and 'abnormal' CA125 readings. In cases where multiple treatments are given on the same day, the treatment date is shifted slightly to show all treatments.

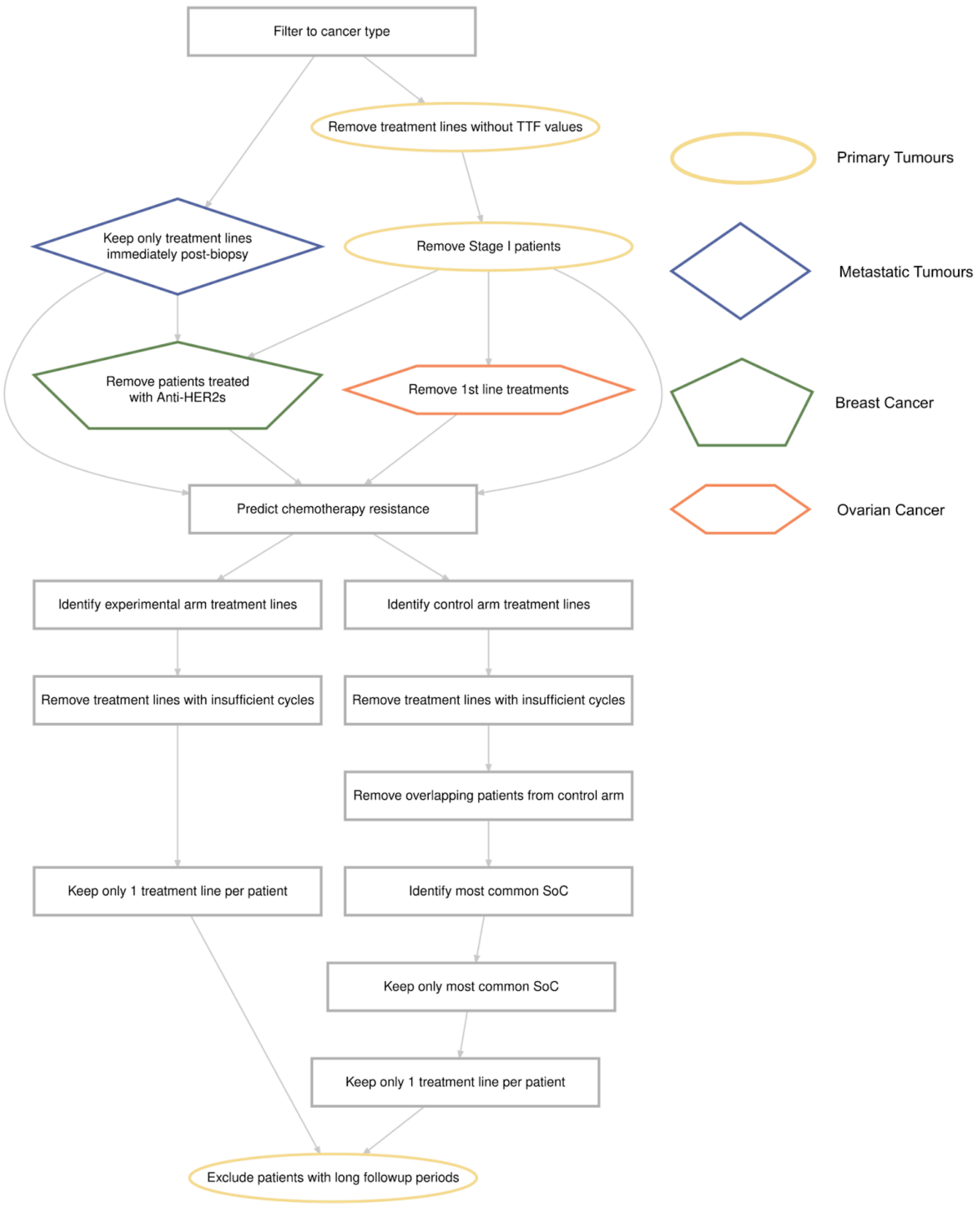

Supplementary Figure 74. Flowchart describing the filtering steps for phase III analysis. Summary of inclusion/exclusion criteria to generate a cohort for emulating phase III randomised clinical trials. Steps that are exclusive for specific tumour types/stages are highlighted in coloured boxed as indicated. Detailed flowcharts for each tumour-type cohort are available in figshare.

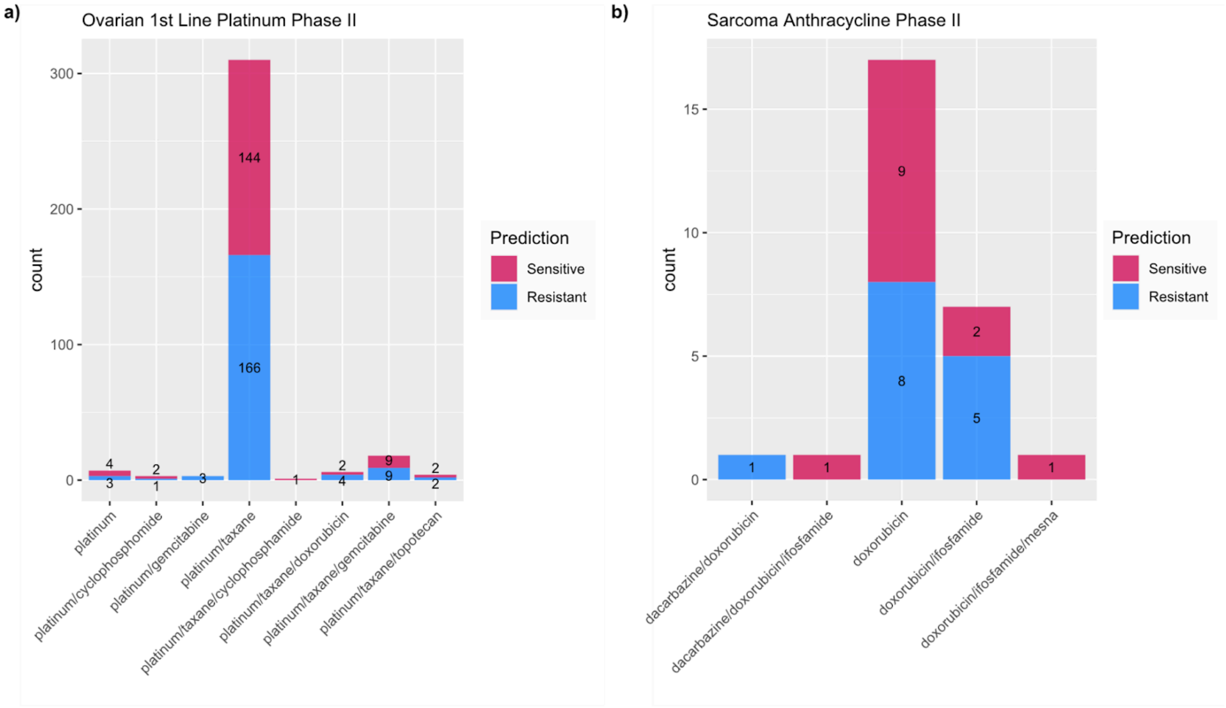

Supplementary Figure 75. Distribution of different treatment combinations. a) 1st-line platinum-based treatments in ovarian cancer patients. **b)** Anthracycline-based treatment in sarcoma patients. Bars indicate the number of patients for each treatment combination. Patients are classified as sensitive and resistant based on biomarker predictions.

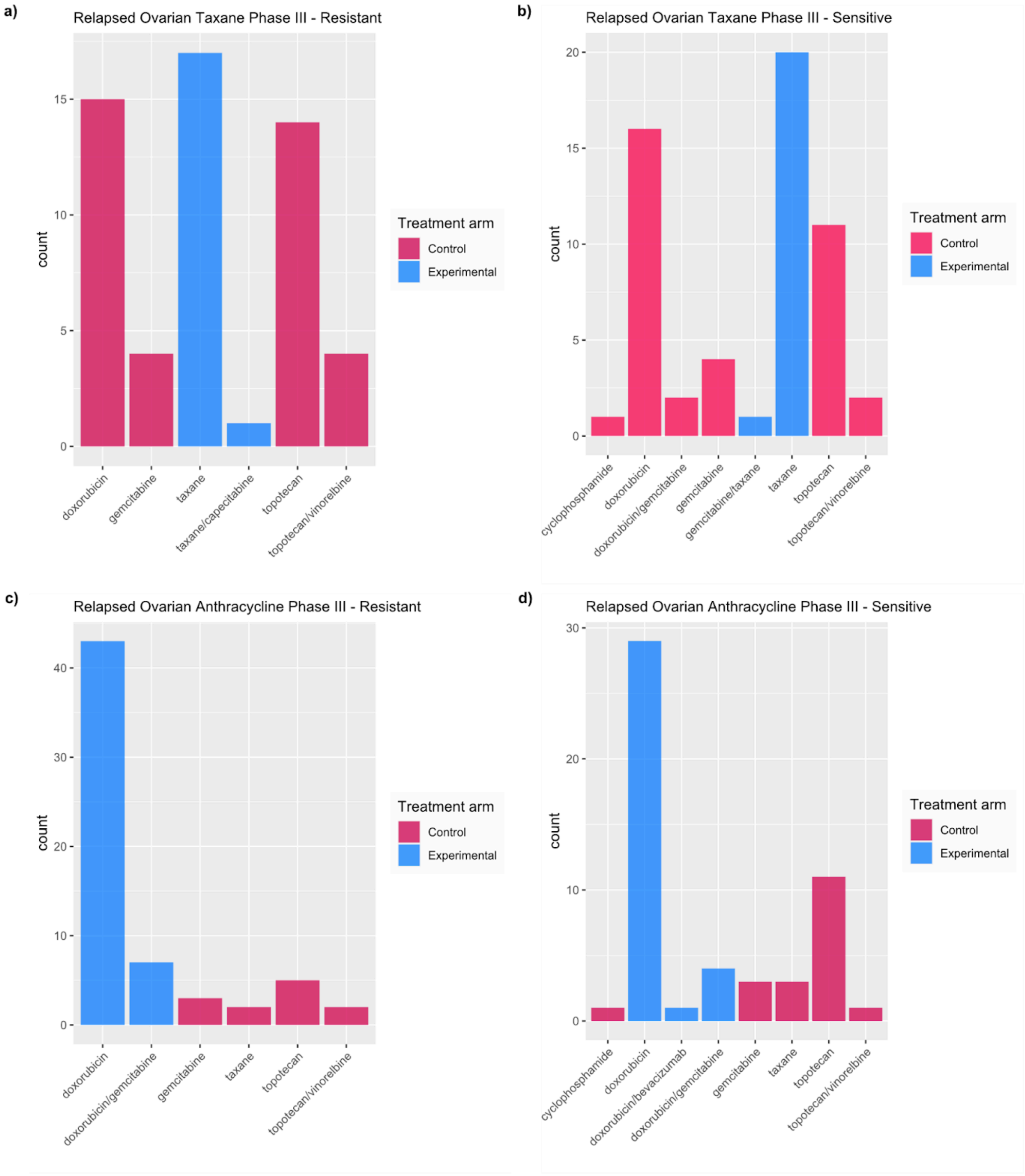

Supplementary Figure 76. Distribution of different treatment combinations in relapsed ovarian cancer. a-b) Taxane-based treatments in ovarian cancer patients predicted as a) resistant and b) sensitive. **c-d)** Anthracycline-based treatments in ovarian cancer patients predicted as c) resistant and d) sensitive. Bars indicate the number of patients for each treatment combination.

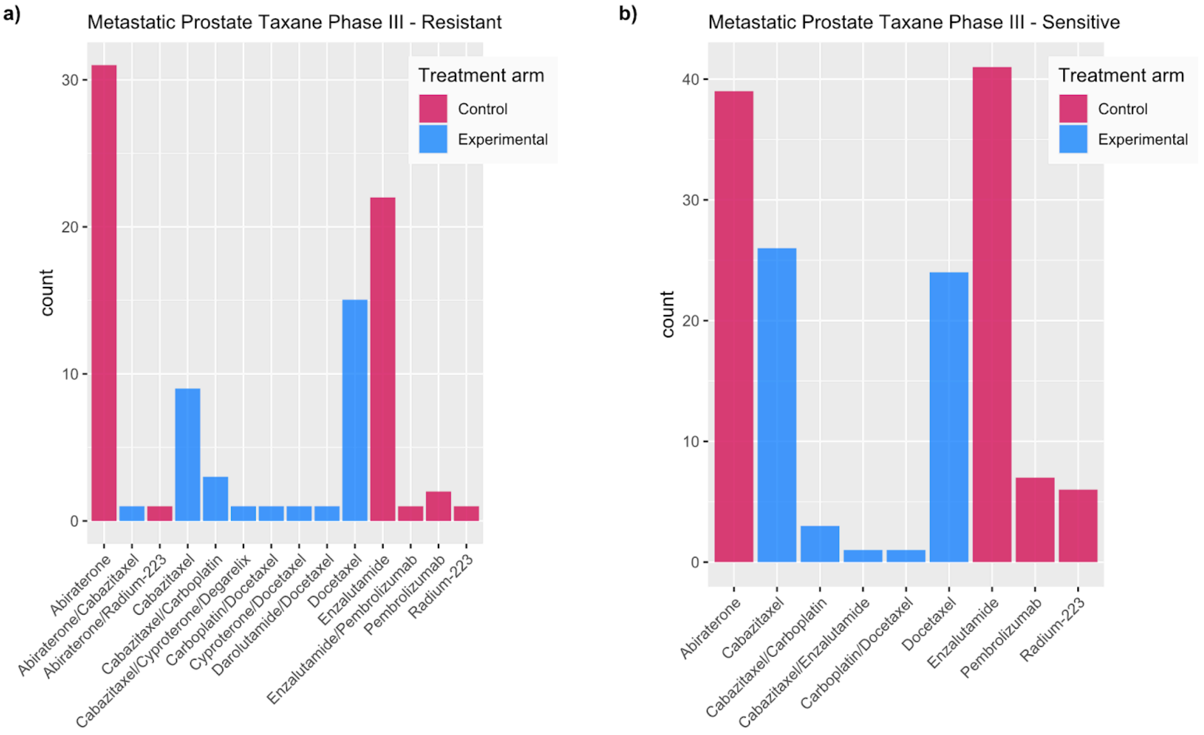

Supplementary Figure 77. Distribution of different taxane-based treatment combinations in metastatic prostate cancer. a) Metastatic prostate cancer patients predicted as resistant. **b)** Metastatic prostate cancer patients predicted as sensitive. Bars indicate the number of patients for each treatment combination.

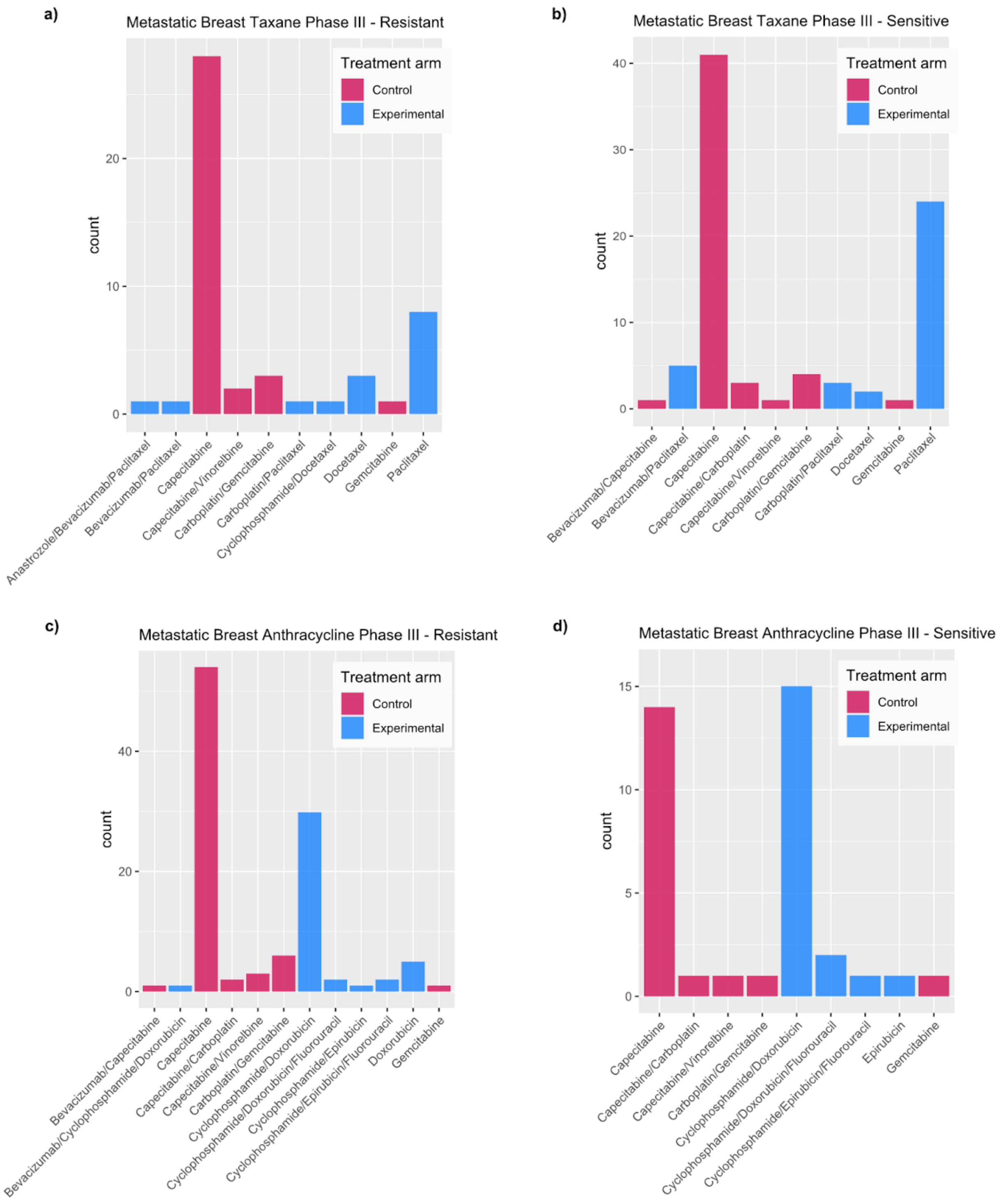

Supplementary Figure 78. Distribution of different treatment combinations in metastatic breast cancer. a-b) Taxane-based treatments in metastatic breast cancer patients predicted as a) resistant and b) sensitive. **c-d)** Anthracycline-based treatments in metastatic breast cancer patients predicted as c) resistant and d) sensitive. Bars indicate the number of patients for each treatment combination.

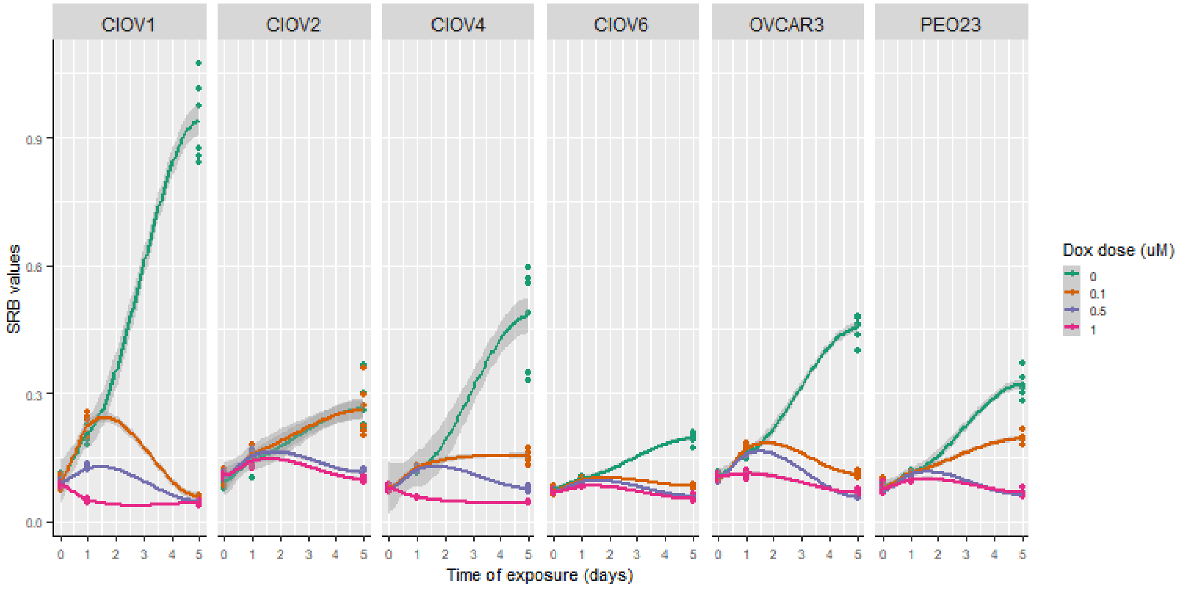

Supplementary Figure 79. Cell growth rates for ovarian cancer lines under doxorubicin treatment for 5 days. Sulforhodamine B (SRB) colorimetric assay used for quantifying cell number in culture.

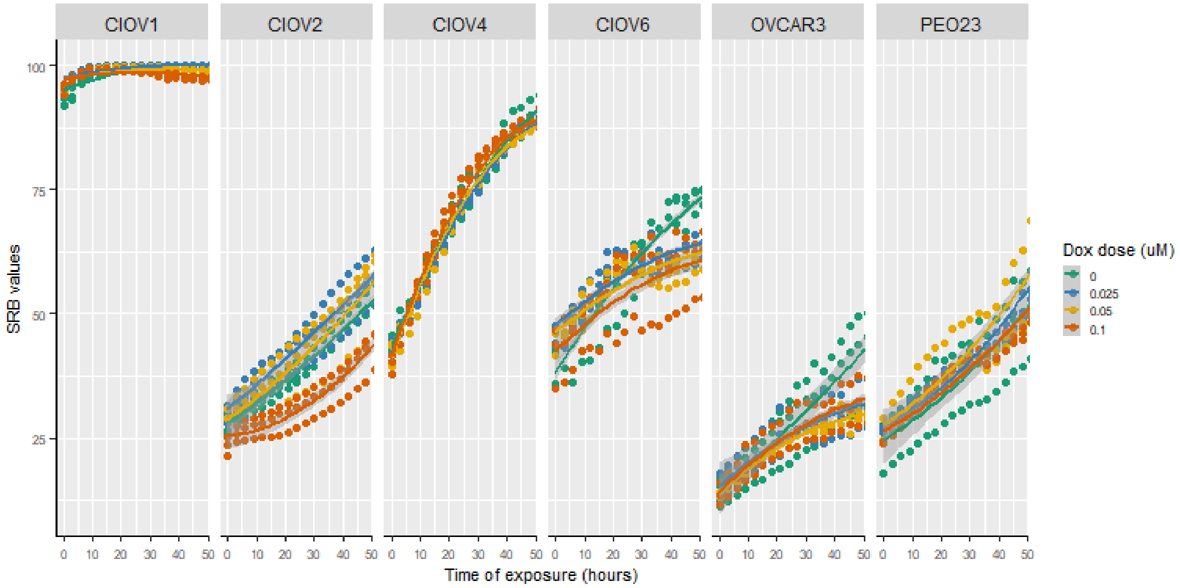

Supplementary Figure 80. Cell growth rates for ovarian cancer lines under low doses of doxorubicin for 48 hours. Sulforhodamine B (SRB) colorimetric assay used for quantifying cell number in culture.

Supplementary References

1. Bhuyan, B. K. *et al.* Comparative genotoxicity of adriamycin and menogarol, two anthracycline antitumor agents. *Cancer Res.* **43**, 5293–5297 (1983).
2. Reimann, H., Stopper, H. & Hintzsche, H. Long-term fate of etoposide-induced micronuclei and micronucleated cells in Hela-H2B-GFP cells. *Arch. Toxicol.* **94**, 3553–3561 (2020).
3. Budke, B. *et al.* Noncanonical NF-κB factor p100/p52 regulates homologous recombination and modulates sensitivity to DNA-damaging therapy. *Nucleic Acids Res.* **50**, 6251–6263 (2022).
4. Chen, M. *et al.* cGAS-STING pathway expression correlates with genomic instability and immune cell infiltration in breast cancer. *NPJ Breast Cancer* **10**, 1 (2024).
5. Hong, C. *et al.* cGAS-STING drives the IL-6-dependent survival of chromosomally instable cancers. *Nature* **607**, 366–373 (2022).
6. Blackledge, G., Lawton, F., Redman, C. & Kelly, K. Response of patients in phase II studies of chemotherapy in ovarian cancer: implications for patient treatment and the design of phase II trials. *Br. J. Cancer* **59**, 650–653 (1989).
7. Bellavia, A., Melloni, G. E. M., Park, J.-G., Discacciati, A. & Murphy, S. A. Estimating and presenting hazard ratios and absolute risks from a Cox model with complex nonlinear interactions. *Am. J. Epidemiol.* **193**, 1155–1160 (2024).
8. Davies, H. *et al.* HRDetect is a predictor of BRCA1 and BRCA2 deficiency based on mutational signatures. *Nat. Med.* **23**, 517–525 (2017).
9. Degasperi, A. *et al.* A practical framework and online tool for mutational signature analyses show inter-tissue variation and driver dependencies. *Nat Cancer* **1**, 249–263 (2020).
10. Telli, M. L. *et al.* Homologous Recombination Deficiency (HRD) Score Predicts Response to Platinum-Containing Neoadjuvant Chemotherapy in Patients with Triple-Negative Breast Cancer. *Clin. Cancer Res.* **22**, 3764–3773 (2016).
11. Marquard, A. M. *et al.* Pan-cancer analysis of genomic scar signatures associated with homologous recombination deficiency suggests novel indications for existing cancer drugs. *Biomark. Res.* **3**, 9 (2015).
12. Knijnenburg, T. A. *et al.* Genomic and molecular landscape of DNA damage repair deficiency across

the cancer genome atlas. *Cell Rep.* **23**, 239–254.e6 (2018).

13. Vias, M. *et al.* High-grade serous ovarian carcinoma organoids as models of chromosomal instability. *Elife* **12**, (2023).
14. Martins, F. C. *et al.* Clonal somatic copy number altered driver events inform drug sensitivity in high-grade serous ovarian cancer. *Nat. Commun.* **13**, 6360 (2022).
15. Macintyre, G. *et al.* Copy number signatures and mutational processes in ovarian carcinoma. *Nat. Genet.* **50**, 1262–1270 (2018).
16. Goranova, T. *et al.* Safety and utility of image-guided research biopsies in relapsed high-grade serous ovarian carcinoma-experience of the BriTROC consortium. *Br. J. Cancer* **116**, 1294–1301 (2017).
17. Villalobos, V. M., Wang, Y. C. & Sikic, B. I. Reannotation and Analysis of Clinical and Chemotherapy Outcomes in the Ovarian Data Set From The Cancer Genome Atlas. *JCO Clin Cancer Inform* **2**, 1–16 (2018).
18. Grossman, R. L. *et al.* Toward a shared vision for cancer genomic data. *N. Engl. J. Med.* **375**, 1109–1112 (2016).
19. Gianni, L. *et al.* Efficacy and safety of neoadjuvant pertuzumab and trastuzumab in women with locally advanced, inflammatory, or early HER2-positive breast cancer (NeoSphere): a randomised multicentre, open-label, phase 2 trial. *Lancet Oncol.* **13**, 25–32 (2012).
20. Buzdar, A. U. *et al.* Significantly higher pathologic complete remission rate after neoadjuvant therapy with trastuzumab, paclitaxel, and epirubicin chemotherapy: results of a randomized trial in human epidermal growth factor receptor 2-positive operable breast cancer. *J. Clin. Oncol.* **23**, 3676–3685 (2005).
21. Joensuu, H. *et al.* Adjuvant docetaxel or vinorelbine with or without trastuzumab for breast cancer. *N. Engl. J. Med.* **354**, 809–820 (2006).
22. Piccart-Gebhart, M. J. *et al.* Trastuzumab after adjuvant chemotherapy in HER2-positive breast cancer. *N. Engl. J. Med.* **353**, 1659–1672 (2005).
23. Romond, E. H. *et al.* Trastuzumab plus adjuvant chemotherapy for operable HER2-positive breast cancer. *N. Engl. J. Med.* **353**, 1673–1684 (2005).

24. de Azambuja, E. *et al.* Lapatinib with trastuzumab for HER2-positive early breast cancer (NeoALTTO): survival outcomes of a randomised, open-label, multicentre, phase 3 trial and their association with pathological complete response. *Lancet Oncol.* **15**, 1137–1146 (2014).

25. Lehmann, B. D. *et al.* Multi-omics analysis identifies therapeutic vulnerabilities in triple-negative breast cancer subtypes. *Nat. Commun.* **12**, 6276 (2021).

26. Lehmann, B. D. *et al.* Refinement of Triple-Negative Breast Cancer Molecular Subtypes: Implications for Neoadjuvant Chemotherapy Selection. *PLoS One* **11**, e0157368 (2016).

27. Thennavan, A. *et al.* Molecular analysis of TCGA breast cancer histologic types. *Cell Genom* **1**, (2021).

28. Kalecky, K., Modisette, R., Pena, S., Cho, Y.-R. & Taube, J. Integrative analysis of breast cancer profiles in TCGA by TNBC subgrouping reveals novel microRNA-specific clusters, including miR-17-92a, distinguishing basal-like 1 and basal-like 2 TNBC subtypes. *BMC Cancer* **20**, 141 (2020).

29. Drews, R. M. *et al.* A pan-cancer compendium of chromosomal instability. *Nature* (2022) doi:10.1038/s41586-022-04789-9.

30. Ghandi, M. *et al.* Next-generation characterization of the Cancer Cell Line Encyclopedia. *Nature* **569**, 503–508 (2019).

31. Corsello, S. M. *et al.* Discovering the anti-cancer potential of non-oncology drugs by systematic viability profiling. *Nat Cancer* **1**, 235–248 (2020).

32. Baert, T. *et al.* The systemic treatment of recurrent ovarian cancer revisited. *Ann. Oncol.* **32**, 710–725 (2021).

33. Ritz, C., Baty, F., Streibig, J. C. & Gerhard, D. Dose-Response Analysis Using R. *PLoS One* **10**, e0146021 (2015).

34. Mutch, D. G. *et al.* Randomized phase III trial of gemcitabine compared with pegylated liposomal doxorubicin in patients with platinum-resistant ovarian cancer. *J. Clin. Oncol.* **25**, 2811–2818 (2007).

35. O'Byrne, K. J. A phase III study of Doxil/Caelyx versus paclitaxel in platinum-treated, taxane-naive relapsed ovarian cancer. *Proc. Annu. Meet. Am. Assoc. Cancer Res.* **21**, 203a (2002).

36. Kaye, S. B. *et al.* Phase II, open-label, randomized, multicenter study comparing the efficacy and

safety of olaparib, a poly (ADP-ribose) polymerase inhibitor, and pegylated liposomal doxorubicin in patients with BRCA1 or BRCA2 mutations and recurrent ovarian cancer. *J. Clin. Oncol.* **30**, 372–379 (2012).

37. Ferrandina, G. *et al.* Phase III trial of gemcitabine compared with pegylated liposomal doxorubicin in progressive or recurrent ovarian cancer. *J. Clin. Oncol.* **26**, 890–896 (2008).
38. Gordon, A. N., Tonda, M., Sun, S., Rackoff, W. & Doxil Study 30-49 Investigators. Long-term survival advantage for women treated with pegylated liposomal doxorubicin compared with topotecan in a phase 3 randomized study of recurrent and refractory epithelial ovarian cancer. *Gynecol. Oncol.* **95**, 1–8 (2004).
39. Pujade-Lauraine, E. *et al.* AURELIA: A randomized phase III trial evaluating bevacizumab (BEV) plus chemotherapy (CT) for platinum (PT)-resistant recurrent ovarian cancer (OC). *J. Clin. Oncol.* **30**, LBA5002–LBA5002 (2012).
40. Rose, P. *et al.* Phase 3 Study: Canfosfamide (C, TLK286) plus carboplatin (P) vs liposomal doxorubicin (D) as 2nd line therapy of platinum (P) resistant ovarian cancer (OC). *J. Clin. Oncol.* **25**, LBA5529–LBA5529 (2007).
41. Gordon, A. N. *et al.* Recurrent epithelial ovarian carcinoma: a randomized phase III study of pegylated liposomal doxorubicin versus topotecan. *J. Clin. Oncol.* **19**, 3312–3322 (2001).
42. Sauer, C. M. *et al.* Molecular landscape and functional characterization of centrosome amplification in ovarian cancer. *Nat. Commun.* **14**, 6505 (2023).
43. Chondrou, V. *et al.* Combined study on clastogenic, aneugenic and apoptotic properties of doxorubicin in human cells in vitro. *J. Biol. Res.* **25**, 17 (2018).
44. Love, M. I., Huber, W. & Anders, S. Moderated estimation of fold change and dispersion for RNA-seq data with DESeq2. *Genome Biol.* **15**, 550 (2014).
45. Schulz, K. F., Altman, D. G., Moher, D. & CONSORT Group. CONSORT 2010 Statement: updated guidelines for reporting parallel group randomised trials. *BMC Med.* **8**, 18 (2010).
46. Hsieh, F. Y. & Lavori, P. W. Sample-size calculations for the Cox proportional hazards regression model with nonbinary covariates. *Control. Clin. Trials* **21**, 552–560 (2000).
47. Tajik, P., Zwinderman, A. H., Mol, B. W. & Bossuyt, P. M. Trial designs for personalizing cancer care:

a systematic review and classification. *Clin. Cancer Res.* **19**, 4578–4588 (2013).

48. Sargent, D. J., Conley, B. A., Allegra, C. & Collette, L. Clinical trial designs for predictive marker validation in cancer treatment trials. *J. Clin. Oncol.* **23**, 2020–2027 (2005).

49. Mandrekar, S. J., An, M.-W. & Sargent, D. J. A review of phase II trial designs for initial marker validation. *Contemp. Clin. Trials* **36**, 597–604 (2013).

50. Emilsson, L. *et al.* Examining bias in studies of statin treatment and survival in patients with cancer. *JAMA Oncol.* **4**, 63–70 (2018).

51. Austin, P. C. & Stuart, E. A. Moving towards best practice when using inverse probability of treatment weighting (IPTW) using the propensity score to estimate causal treatment effects in observational studies. *Stat. Med.* **34**, 3661–3679 (2015).

52. Noah Greifer. WeightIt: Weighting for Covariate Balance in Observational Studies. R package version 1.3.2. https://CRAN.R-project.org/package=WeightIt. (2024).
