# Supplementary material for: Predicting resistance to chemotherapy using chromosomal instability signatures
Source: Nat Genet. 2025 Jun 23;57(7):1708–17. doi: 10.1038/s41588-025-02233-y (PMC12283407; doi:10.1038/s41588-025-02233-y)
Supplement: Supplementary file 2 — Reporting Summary [file 41588_2025_2233_MOESM2_ESM.pdf]

nature portfolio

Corresponding author(s): Geoff Macintyre, Anna Piskorz

Last updated by author(s): 02/04/2025

Reporting Summary

Nature Portfolio wishes to improve the reproducibility of the work that we publish. This form provides structure for consistency and transparency in reporting. For further information on Nature Portfolio policies, see our Editorial Policies and the Editorial Policy Checklist.

Statistics

For all statistical analyses, confirm that the following items are present in the figure legend, table legend, main text, or Methods section.

n/a	Confirmed	
☐	☒	The exact sample size (*n*) for each experimental group/condition, given as a discrete number and unit of measurement
☐	☒	A statement on whether measurements were taken from distinct samples or whether the same sample was measured repeatedly
☐	☒	The statistical test(s) used AND whether they are one- or two-sided<br>*Only common tests should be described solely by name; describe more complex techniques in the Methods section.*
☐	☒	A description of all covariates tested
☐	☒	A description of any assumptions or corrections, such as tests of normality and adjustment for multiple comparisons
☐	☒	A full description of the statistical parameters including central tendency (e.g. means) or other basic estimates (e.g. regression coefficient) AND variation (e.g. standard deviation) or associated estimates of uncertainty (e.g. confidence intervals)
☐	☒	For null hypothesis testing, the test statistic (e.g. *F*, *t*, *r*) with confidence intervals, effect sizes, degrees of freedom and *P* value noted<br>*Give P values as exact values whenever suitable.*
☒	☐	For Bayesian analysis, information on the choice of priors and Markov chain Monte Carlo settings
☒	☐	For hierarchical and complex designs, identification of the appropriate level for tests and full reporting of outcomes
☒	☐	Estimates of effect sizes (e.g. Cohen's *d*, Pearson's *r*), indicating how they were calculated

Our web collection on statistics for biologists contains articles on many of the points above.

Software and code

Policy information about availability of computer code

Data collection

All software is currently open for review via the github repository:
For most analyses R (v4.2.2) free statistical software was used. The following packages were used:
AnnotationDbi 1.56.2
Biobase 2.54.0
BiocGenerics 0.40.0
broom 1.0.3
car 3.1.1
CNpare 0.99.0
cobalt 4.5.5
data.table 1.14.8
DESeq2 1.34.0
DiagrammeR 1.0.11
DiagrammeRsvg 0.1
doMC 1.3.8
doParallel 1.0.17
dplyr 1.1.0
drc 3.0-1
fgsea 1.20.0
foreach 1.5.2
freshr 1.0.2
GenomicDataCommons 1.18.0

GenomicRanges 1.46.1
GenomeInfoDb 1.30.1
ggplot2 3.5.1
ggpubr 0.6.0
ggthemes 4.2.4
grid 4.1.2
iterators 1.0.14
IRanges 2.28.0
interactionRCS 0.1.1
knitr 1.42
MatrixGenerics 1.6.0
matrixStats 0.63.0
mclust 6.1.1
org.Hs.eg.db 3.14.0
QDNAseq 1.30.0
QDNAseqmod 1.21.0
readr 2.1.4
readxl 1.4.2
reshape 0.8.9
reshape2 1.4.4
rlang 1.1.3
rstudioapi 0.14
rsvg 2.6.0
S4Vectors 0.32.4
stats4 4.1.2
stringr 1.5.0
SummarizedExperiment 1.24.0
survival 3.4.0
survminer 0.4.9
swimplot 1.2.0
TCGAbiolinks 2.29.6
this.path 1.2.0
UpSetR 1.4.0
WeightIt 1.3.2
xml2 1.3.3
YAPSA 1.32.0

Data analysis

Generating copy-number profiles
Reads were aligned using bwa-mem (v0.7.17)
Duplicate reads were identified and marked using MarkDuplicates from the GATK toolsuite (v4.1.8.1).
A modified version of the QDNAseq R package (v1.21.0, available here: https://github.com/gmaci/QDNAseqmod) was used to fit absolute copy-number profiles from mapped reads.

Computing copy number signatures
Pancancer copy number signatures were computed using the YAPSA package from R (v.1.32.0)

Chemotherapy response predictions
Code available in Github repository

Gene enrichment analysis
Differential expression analysis was performed using the DESeq2 R package (v1.34.0)

Survival analysis:
Kaplan Meier analysis: survfit function from survival R package (v3.5-5)
Cox proportional hazard models: coxph function from survival R package (v3.5-5)
Interpretation of interactions between covariates: intEST function from interactionRCS package (v0.1.1)
Inverse Probability Weighting: weightit function from the WeightIt package (v1.3.3)

Plasma-Tissue concordance
Absolute copy number profiles were compared using the getDifference function from the CNpare R package (https://github.com/macintyrelab/CNpare, accessed 2023-07-20)

Statistical analyses
Cosine similarity
Linear regression: lm function from base R (v4.2.2)
Wilcoxon rank test: wilcox.test function from stats4 (v4.1.2)

For manuscripts utilizing custom algorithms or software that are central to the research but not yet described in published literature, software must be made available to editors and reviewers. We strongly encourage code deposition in a community repository (e.g. GitHub). See the Nature Portfolio guidelines for submitting code & software for further information.

Data

Policy information about availability of data

All manuscripts must include a data availability statement. This statement should provide the following information, where applicable:

- Accession codes, unique identifiers, or web links for publicly available datasets
- A description of any restrictions on data availability
- For clinical datasets or third party data, please ensure that the statement adheres to our policy

All data used in this study is described in detail in Supplementary Table 8.
All the required input data to reproduce the code supporting this publication is available at the Github repository accompanying this submission. All the outputs from the code supporting this publication are available without restriction either at the Github repository or the accompanying figshare. Raw data generated in this study is available through the European Genome-phenome Archive (EGA) at the following link: .
OV04 clinical and raw data: Provided by the Brenton lab
OV04 copy number profiles: Generated in this project. Available via the accompanying Github repository.
Cell line, organoid, and spheroid doxorubicin dose-response data: Generated in this project. Available via the Github repository
TCGA copy number profiles: Drews et al 2022. Files hosted on http://github.com/VanLoo-lab/ascat
TCGA clinical data: Genomic Data Commons Data Portal: https://portal.gdc.cancer.gov/
Transcriptomics data for the TCGA ovarian cancer samples: Genomic Data Commons Data Portal: https://portal.gdc.cancer.gov/
TTF data for TCGA ovarian cancer samples: Villalobos, Wang, and Sikic 2018 JCO Clinical Cancer Informatics doi:10.1200/CCI.17.00096
Triple negative status for TCGA breast cancer samples: Lehmann et al 2021, accessed via https://github.com/TransBioInfoLab/TNBC_analysis
Triple negative status for TCGA breast cancer samples: Kalecky et al 2020 BMC Cancer doi:10.1186/s12885-020-6600-6.
Triple negative status for TCGA breast cancer samples: Thennavan et al 2021 Cell Genomics doi:10.1016/j.xgen.2021.100067.
ER/PR/HER2 status for TCGA breast cancer samples: Lehmann et al 2016 PloS One doi:10.1371/journal.pone.0157368
ER/PR/HER2 status for TCGA breast cancer samples: Thennavan et al 2021 Cell Genomics doi:10.1016/j.xgen.2021.100067.
Hallmark pathways for differential expression analysis: Human MSigDB Collections: https://www.gsea-msigdb.org/gsea/msigdb/index.jsp
HMF clinical and raw data: Provided by HMF as part of data access request DR-343
PRISM drug repurposing screen: Corsello et al. 2020. Files accessed via https://github.com/macintyrelab/CINSignatureBiomarkerAnalysis
Copy number profiles of cell lines from the CCLE project: Drews et al 2022. Files hosted on https://github.com/macintyrelab/CINSignatureBiomarkerAnalysis

Research involving human participants, their data, or biological material

Policy information about studies with human participants or human data. See also policy information about sex, gender (identity/presentation), and sexual orientation and race, ethnicity and racism.

Reporting on sex and gender	This study did not do any sex or gender-based analyses. This was because we expect our findings to equally apply to individuals of both sexes/genders.
Reporting on race, ethnicity, or other socially relevant groupings	This study does not report on race, ethnic groups or other socially relevant subgroupings. This was because we did not expect these factors to be significant covariates in any analyses.
Population characteristics	The only covariate-relevant population characteristics in this study were age at diagnosis, tumour stage and subtypes. These covariates were included as part of multivariate Cox proportional hazard models. Population characteristics for OV04 can be found in Supplementary Table 1 and characteristics for the TCGA and HMF cohorts can be found in Supplementary Table 5.
Recruitment	No patients were recruited as part of this project. Patient data used in this project had previously been recruited as part of the ongoing CTCROV04 observational study, and for that purpose patients provided written, informed consent for participation in this study and for the use of their donated tissue for the laboratory studies carried out in this work.<br>All the remaining data was collected from published sources, of which we did not have control over the recruitment process
Ethics oversight	Ethics approval for the CTCROV04 study was given by the Institutional Ethics Committee (REC07/Q0106/63, REC08/H0306/61 and REC07/Q0106/63).

Note that full information on the approval of the study protocol must also be provided in the manuscript.

Field-specific reporting

Please select the one below that is the best fit for your research. If you are not sure, read the appropriate sections before making your selection.

☒ Life sciences ☐ Behavioural & social sciences ☐ Ecological, evolutionary & environmental sciences

For a reference copy of the document with all sections, see nature.com/documents/nr-reporting-summary-flat.pdf

Life sciences study design

All studies must disclose on these points even when the disclosure is negative.

Sample size	(Numbers represented below are after filtering data)<br>Ovarian cancer cell lines: 4 cell lines

OV04 organoids: 8 organoids
OV04 spheroids: 15 spheroids
OV04 tissue: 50 patients
OV04 plasma: 9 patients
TCGA: 7,105 patients
TCGA-OV Platinum Phase II Single-arm study: 352 patients
TCGA-OV Taxane Phase III Randomised Control Study: 112 patients
TCGA-OV Anhtracycline Phase III Randomised Control Study: 115 patients
TCGA-SARC Anhtracycline Phase II Single-arm study: 27 patients
HMF: 7,437 patients
HMF-Prostate Taxane Phase III Randomised Control Study: 238 patients
HMF-Breast Taxane Phase III Randomised Control Study: 134 patients
HMF-Breast Anthracycline Enriched Arm Trial Study: 108 patients

Sample sizes for TCGA and HMF cohorts were checked against power calculations following the approach in 'Methods - Power Analysis'. OV04 and the cancer cell line analyses were considered initial pilot studies and as such were not subject to sample size requirements. However a literature search of published phase II biomarker trials showed that the sample sizes used in the OV04 analyses were within a similar range.

Data exclusions

This information is also provided as part of the Methods section of the manuscript.
Cell lines that showed 100% confluence after 48 hours of being exposed to low doses of doxorubicin were discarded from further analyses due to signal saturation.
Organoids and spheroids were removed from analysis if they showed more then 20% standard deviation across more than 3 dose concentrations when treated with doxorubicin.
The copy number profiles of tissue samples from OV04 were manually curated according to a 3-star rating system, and 1-star samples were removed from downstream analysis.
The copy number profiles of plasma samples from OV04 were manually curated to identify proportions of ctDNA for each sample. Those with low levels of ctDNA were removed from downstream analysis.
For OV04 paclitaxel analysis, only patients that had been treated with post-1st line single-agent paclitaxel were kept. Additionally patients were removed if the single-agent paclitaxel treatment involved <3 or >25 cycles of paclitaxel.
For OV04 doxorubicin analysis, patients who received a combination of doxorubicin and platinum at 1st line were removed. Additionally patients were removed if treatment involved <3 cycles of doxorubicin.
TCGA and HMF patients were also removed if their clinical drug treatment data was too sparse for TTF calculation, or clinical characteristics were not available. In metastatic cases, only treatment lines that occurred immediately following a biopsy were used for the survival analyses.

Replication

The platinum response predictor was initially developed using data from the TCGA and the Pan-Cancer Analysis of Whole Genomes (PCAWG) Dataset in Drews et al 2022. In this study, this classifier is reformulated to be applied across multiple tumour types. Then, we tested the pan-cancer applicability in the TCGA-ESCA cohort, and validated in the OV04 and TCGA-OV cohorts via Phase II single-arm trial emulation.
The taxane response predictor is optimised using cell lines from the DepMap project, tested in the OV04 cohort using a Phase II single-arm trial emulation, and validated in the TCGA-OV, HMF-Prostate and HMF-Breast cohorts via Phase III randomised controlled study emulation.
The doxorubicin response predictor is developed in a combination of cell lines, patient-derived organoids and spheroids, tested in the OV04 cohort using a Phase II single-arm trial emulation, and validated in the TCGA-OV and HMF-Breast cohorts via Phase III randomised controlled study emulation and in the TCGA-SARC cohort via Phase II single-arm trial emulation.

Randomization

No randomization was performed - this was a descriptive study, not an experimental study.

Blinding

No blinding was undertaken - this was a descriptive study, not an experimental study.

Behavioural & social sciences study design

All studies must disclose on these points even when the disclosure is negative.

Study description

Briefly describe the study type including whether data are quantitative, qualitative, or mixed-methods (e.g. qualitative cross-sectional, quantitative experimental, mixed-methods case study).

Research sample

State the research sample (e.g. Harvard university undergraduates, villagers in rural India) and provide relevant demographic information (e.g. age, sex) and indicate whether the sample is representative. Provide a rationale for the study sample chosen. For studies involving existing datasets, please describe the dataset and source.

Sampling strategy

Describe the sampling procedure (e.g. random, snowball, stratified, convenience). Describe the statistical methods that were used to predetermine sample size OR if no sample-size calculation was performed, describe how sample sizes were chosen and provide a rationale for why these sample sizes are sufficient. For qualitative data, please indicate whether data saturation was considered, and what criteria were used to decide that no further sampling was needed.

Data collection

Provide details about the data collection procedure, including the instruments or devices used to record the data (e.g. pen and paper, computer, eye tracker, video or audio equipment) whether anyone was present besides the participant(s) and the researcher, and whether the researcher was blind to experimental condition and/or the study hypothesis during data collection.

Timing

Indicate the start and stop dates of data collection. If there is a gap between collection periods, state the dates for each sample cohort.

Data exclusions

If no data were excluded from the analyses, state so OR if data were excluded, provide the exact number of exclusions and the rationale behind them, indicating whether exclusion criteria were pre-established.

Non-participation	*State how many participants dropped out/declined participation and the reason(s) given OR provide response rate OR state that no participants dropped out/declined participation.*
Randomization	*If participants were not allocated into experimental groups, state so OR describe how participants were allocated to groups, and if allocation was not random, describe how covariates were controlled.*

Ecological, evolutionary & environmental sciences study design

All studies must disclose on these points even when the disclosure is negative.

Study description	*Briefly describe the study. For quantitative data include treatment factors and interactions, design structure (e.g. factorial, nested, hierarchical), nature and number of experimental units and replicates.*
Research sample	*Describe the research sample (e.g. a group of tagged Passer domesticus, all Stenocereus thurberi within Organ Pipe Cactus National Monument), and provide a rationale for the sample choice. When relevant, describe the organism taxa, source, sex, age range and any manipulations. State what population the sample is meant to represent when applicable. For studies involving existing datasets, describe the data and its source.*
Sampling strategy	*Note the sampling procedure. Describe the statistical methods that were used to predetermine sample size OR if no sample-size calculation was performed, describe how sample sizes were chosen and provide a rationale for why these sample sizes are sufficient.*
Data collection	*Describe the data collection procedure, including who recorded the data and how.*
Timing and spatial scale	*Indicate the start and stop dates of data collection, noting the frequency and periodicity of sampling and providing a rationale for these choices. If there is a gap between collection periods, state the dates for each sample cohort. Specify the spatial scale from which the data are taken*
Data exclusions	*If no data were excluded from the analyses, state so OR if data were excluded, describe the exclusions and the rationale behind them, indicating whether exclusion criteria were pre-established.*
Reproducibility	*Describe the measures taken to verify the reproducibility of experimental findings. For each experiment, note whether any attempts to repeat the experiment failed OR state that all attempts to repeat the experiment were successful.*
Randomization	*Describe how samples/organisms/participants were allocated into groups. If allocation was not random, describe how covariates were controlled. If this is not relevant to your study, explain why.*
Blinding	*Describe the extent of blinding used during data acquisition and analysis. If blinding was not possible, describe why OR explain why blinding was not relevant to your study.*

Did the study involve field work? ☐ Yes ☐ No

Field work, collection and transport

Field conditions	*Describe the study conditions for field work, providing relevant parameters (e.g. temperature, rainfall).*
Location	*State the location of the sampling or experiment, providing relevant parameters (e.g. latitude and longitude, elevation, water depth).*
Access & import/export	*Describe the efforts you have made to access habitats and to collect and import/export your samples in a responsible manner and in compliance with local, national and international laws, noting any permits that were obtained (give the name of the issuing authority, the date of issue, and any identifying information).*
Disturbance	*Describe any disturbance caused by the study and how it was minimized.*

Reporting for specific materials, systems and methods

We require information from authors about some types of materials, experimental systems and methods used in many studies. Here, indicate whether each material, system or method listed is relevant to your study. If you are not sure if a list item applies to your research, read the appropriate section before selecting a response.

Materials & experimental systems

n/a	Involved in the study	
☐	☒	Antibodies
☐	☒	Eukaryotic cell lines
☒	☐	Palaeontology and archaeology
☒	☐	Animals and other organisms
☒	☐	Clinical data
☒	☐	Dual use research of concern
☒	☐	Plants

Methods

n/a	Involved in the study	
☒	☐	ChIP-seq
☒	☐	Flow cytometry
☒	☐	MRI-based neuroimaging

Antibodies

Antibodies used: phospho-histo H3 (pHH3; 06-570 Merck) and secondary antibody (Alexa Fluor 555, 1µg/ml; A-21429 Invitrogen) were used as part of the micronuclei counting described in the Methods.

Validation: Antibody validation information is available from the manufacturers (antibodies are pre-validated by manufacturer).

Eukaryotic cell lines

Policy information about cell lines and Sex and Gender in Research

Cell line source(s): Two of the 4 ovarian cancer cell lines (CIOV2 and CIOV4) were derived in-house from OV04 patients, OVCAR3 was obtained from the American Type Culture Collection, and PEO23 was a kind gift from Langdon lab.

Authentication: Cell line identities were confirmed by STR profiling.

Mycoplasma contamination: Cells were regularly screened for mycoplasma using a MycoAlert Mycoplasma Detection Kit (Lonza). We confirm that cells tested always negative in the Mycoplasma assays.

Commonly misidentified lines (See ICLAC register): No misidentified lines were used as part of this study

Palaeontology and Archaeology

Specimen provenance: *Provide provenance information for specimens and describe permits that were obtained for the work (including the name of the issuing authority, the date of issue, and any identifying information). Permits should encompass collection and, where applicable, export.*

Specimen deposition: *Indicate where the specimens have been deposited to permit free access by other researchers.*

Dating methods: *If new dates are provided, describe how they were obtained (e.g. collection, storage, sample pretreatment and measurement), where they were obtained (i.e. lab name), the calibration program and the protocol for quality assurance OR state that no new dates are provided.*

☐ Tick this box to confirm that the raw and calibrated dates are available in the paper or in Supplementary Information.

Ethics oversight: *Identify the organization(s) that approved or provided guidance on the study protocol, OR state that no ethical approval or guidance was required and explain why not.*

Note that full information on the approval of the study protocol must also be provided in the manuscript.

Animals and other research organisms

Policy information about studies involving animals; ARRIVE guidelines recommended for reporting animal research, and Sex and Gender in Research

Laboratory animals: *For laboratory animals, report species, strain and age OR state that the study did not involve laboratory animals.*

Wild animals: *Provide details on animals observed in or captured in the field; report species and age where possible. Describe how animals were caught and transported and what happened to captive animals after the study (if killed, explain why and describe method; if released, say where and when) OR state that the study did not involve wild animals.*

Reporting on sex: *Indicate if findings apply to only one sex; describe whether sex was considered in study design, methods used for assigning sex. Provide data disaggregated for sex where this information has been collected in the source data as appropriate; provide overall*

	numbers in this Reporting Summary. Please state if this information has not been collected. Report sex-based analyses where performed, justify reasons for lack of sex-based analysis.
Field-collected samples	*For laboratory work with field-collected samples, describe all relevant parameters such as housing, maintenance, temperature, photoperiod and end-of-experiment protocol OR state that the study did not involve samples collected from the field.*
Ethics oversight	*Identify the organization(s) that approved or provided guidance on the study protocol, OR state that no ethical approval or guidance was required and explain why not.*

Note that full information on the approval of the study protocol must also be provided in the manuscript.

Clinical data

Policy information about clinical studies

All manuscripts should comply with the ICMJE guidelines for publication of clinical research and a completed CONSORT checklist must be included with all submissions.

Clinical trial registration	*Provide the trial registration number from ClinicalTrials.gov or an equivalent agency.*
Study protocol	*Note where the full trial protocol can be accessed OR if not available, explain why.*
Data collection	*Describe the settings and locales of data collection, noting the time periods of recruitment and data collection.*
Outcomes	*Describe how you pre-defined primary and secondary outcome measures and how you assessed these measures.*

Dual use research of concern

Policy information about dual use research of concern

Hazards

Could the accidental, deliberate or reckless misuse of agents or technologies generated in the work, or the application of information presented in the manuscript, pose a threat to:

No	Yes	
☐	☐	Public health
☐	☐	National security
☐	☐	Crops and/or livestock
☐	☐	Ecosystems
☐	☐	Any other significant area

Experiments of concern

Does the work involve any of these experiments of concern:

No	Yes	
☐	☐	Demonstrate how to render a vaccine ineffective
☐	☐	Confer resistance to therapeutically useful antibiotics or antiviral agents
☐	☐	Enhance the virulence of a pathogen or render a nonpathogen virulent
☐	☐	Increase transmissibility of a pathogen
☐	☐	Alter the host range of a pathogen
☐	☐	Enable evasion of diagnostic/detection modalities
☐	☐	Enable the weaponization of a biological agent or toxin
☐	☐	Any other potentially harmful combination of experiments and agents

Plants

Seed stocks

Report on the source of all seed stocks or other plant material used. If applicable, state the seed stock centre and catalogue number. If plant specimens were collected from the field, describe the collection location, date and sampling procedures.

Novel plant genotypes

Describe the methods by which all novel plant genotypes were produced. This includes those generated by transgenic approaches, gene editing, chemical/radiation-based mutagenesis and hybridization. For transgenic lines, describe the transformation method, the number of independent lines analyzed and the generation upon which experiments were performed. For gene-edited lines, describe the editor used, the endogenous sequence targeted for editing, the targeting guide RNA sequence (if applicable) and how the editor was applied.

Authentication

Describe any authentication procedures for each seed stock used or novel genotype generated. Describe any experiments used to assess the effect of a mutation and, where applicable, how potential secondary effects (e.g. second site T-DNA insertions, mosiacism, off-target gene editing) were examined.

ChIP-seq

Data deposition

☐ Confirm that both raw and final processed data have been deposited in a public database such as GEO.

☐ Confirm that you have deposited or provided access to graph files (e.g. BED files) for the called peaks.

Data access links
May remain private before publication.

For "Initial submission" or "Revised version" documents, provide reviewer access links. For your "Final submission" document, provide a link to the deposited data.

Files in database submission

Provide a list of all files available in the database submission.

Genome browser session
(e.g. UCSC)

Provide a link to an anonymized genome browser session for "Initial submission" and "Revised version" documents only, to enable peer review. Write "no longer applicable" for "Final submission" documents.

Methodology

Replicates

Describe the experimental replicates, specifying number, type and replicate agreement.

Sequencing depth

Describe the sequencing depth for each experiment, providing the total number of reads, uniquely mapped reads, length of reads and whether they were paired- or single-end.

Antibodies

Describe the antibodies used for the ChIP-seq experiments; as applicable, provide supplier name, catalog number, clone name, and lot number.

Peak calling parameters

Specify the command line program and parameters used for read mapping and peak calling, including the ChIP, control and index files used.

Data quality

Describe the methods used to ensure data quality in full detail, including how many peaks are at FDR 5% and above 5-fold enrichment.

Software

Describe the software used to collect and analyze the ChIP-seq data. For custom code that has been deposited into a community repository, provide accession details.

Flow Cytometry

Plots

Confirm that:

☐ The axis labels state the marker and fluorochrome used (e.g. CD4-FITC).

☐ The axis scales are clearly visible. Include numbers along axes only for bottom left plot of group (a 'group' is an analysis of identical markers).

☐ All plots are contour plots with outliers or pseudocolor plots.

☐ A numerical value for number of cells or percentage (with statistics) is provided.

Methodology

Sample preparation

Describe the sample preparation, detailing the biological source of the cells and any tissue processing steps used.

Instrument

Identify the instrument used for data collection, specifying make and model number.

Software

Describe the software used to collect and analyze the flow cytometry data. For custom code that has been deposited into a community repository, provide accession details.

Cell population abundance

Describe the abundance of the relevant cell populations within post-sort fractions, providing details on the purity of the samples and how it was determined.

Gating strategy

Describe the gating strategy used for all relevant experiments, specifying the preliminary FSC/SSC gates of the starting cell population, indicating where boundaries between "positive" and "negative" staining cell populations are defined.

☐ Tick this box to confirm that a figure exemplifying the gating strategy is provided in the Supplementary Information.

Magnetic resonance imaging

Experimental design

Design type	*Indicate task or resting state; event-related or block design.*
Design specifications	*Specify the number of blocks, trials or experimental units per session and/or subject, and specify the length of each trial or block (if trials are blocked) and interval between trials.*
Behavioral performance measures	*State number and/or type of variables recorded (e.g. correct button press, response time) and what statistics were used to establish that the subjects were performing the task as expected (e.g. mean, range, and/or standard deviation across subjects).*

Acquisition

Imaging type(s)	*Specify: functional, structural, diffusion, perfusion.*
Field strength	*Specify in Tesla*
Sequence & imaging parameters	*Specify the pulse sequence type (gradient echo, spin echo, etc.), imaging type (EPI, spiral, etc.), field of view, matrix size, slice thickness, orientation and TE/TR/flip angle.*
Area of acquisition	*State whether a whole brain scan was used OR define the area of acquisition, describing how the region was determined.*

Diffusion MRI ☐ Used ☐ Not used

Preprocessing

Preprocessing software	*Provide detail on software version and revision number and on specific parameters (model/functions, brain extraction, segmentation, smoothing kernel size, etc.).*
Normalization	*If data were normalized/standardized, describe the approach(es): specify linear or non-linear and define image types used for transformation OR indicate that data were not normalized and explain rationale for lack of normalization.*
Normalization template	*Describe the template used for normalization/transformation, specifying subject space or group standardized space (e.g. original Talairach, MNI305, ICBM152) OR indicate that the data were not normalized.*
Noise and artifact removal	*Describe your procedure(s) for artifact and structured noise removal, specifying motion parameters, tissue signals and physiological signals (heart rate, respiration).*
Volume censoring	*Define your software and/or method and criteria for volume censoring, and state the extent of such censoring.*

Statistical modeling & inference

Model type and settings	*Specify type (mass univariate, multivariate, RSA, predictive, etc.) and describe essential details of the model at the first and second levels (e.g. fixed, random or mixed effects; drift or auto-correlation).*
Effect(s) tested	*Define precise effect in terms of the task or stimulus conditions instead of psychological concepts and indicate whether ANOVA or factorial designs were used.*

Specify type of analysis: ☐ Whole brain ☐ ROI-based ☐ Both

Statistic type for inference (See Eklund et al. 2016)	*Specify voxel-wise or cluster-wise and report all relevant parameters for cluster-wise methods.*
Correction	*Describe the type of correction and how it is obtained for multiple comparisons (e.g. FWE, FDR, permutation or Monte Carlo).*

Models & analysis

n/a	Involved in the study
☐	☐ Functional and/or effective connectivity
☐	☐ Graph analysis
☐	☐ Multivariate modeling or predictive analysis

Functional and/or effective connectivity	*Report the measures of dependence used and the model details (e.g. Pearson correlation, partial correlation, mutual information).*
Graph analysis	*Report the dependent variable and connectivity measure, specifying weighted graph or binarized graph, subject- or group-level, and the global and/or node summaries used (e.g. clustering coefficient, efficiency, etc.).*
Multivariate modeling and predictive analysis	*Specify independent variables, features extraction and dimension reduction, model, training and evaluation metrics.*
